# Supplementary figures and images for: Pleiotropic effects of BAFF on the senescence-associated secretome and growth arrest
Source: eLife. 2023 Apr 21;12:e84238. doi: 10.7554/eLife.84238 (PMC10121226; doi:10.7554/eLife.84238)

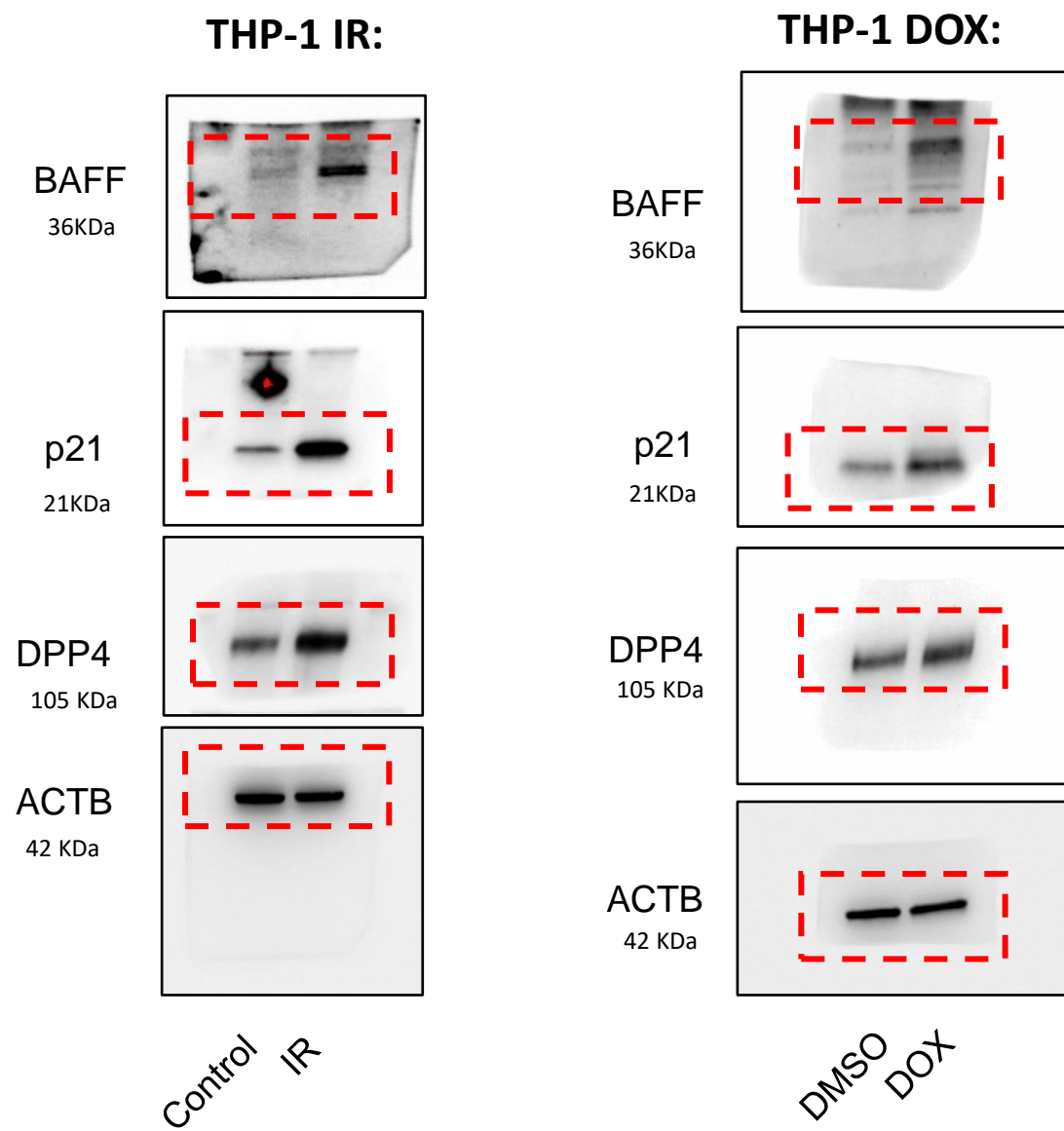**WI-38 IR**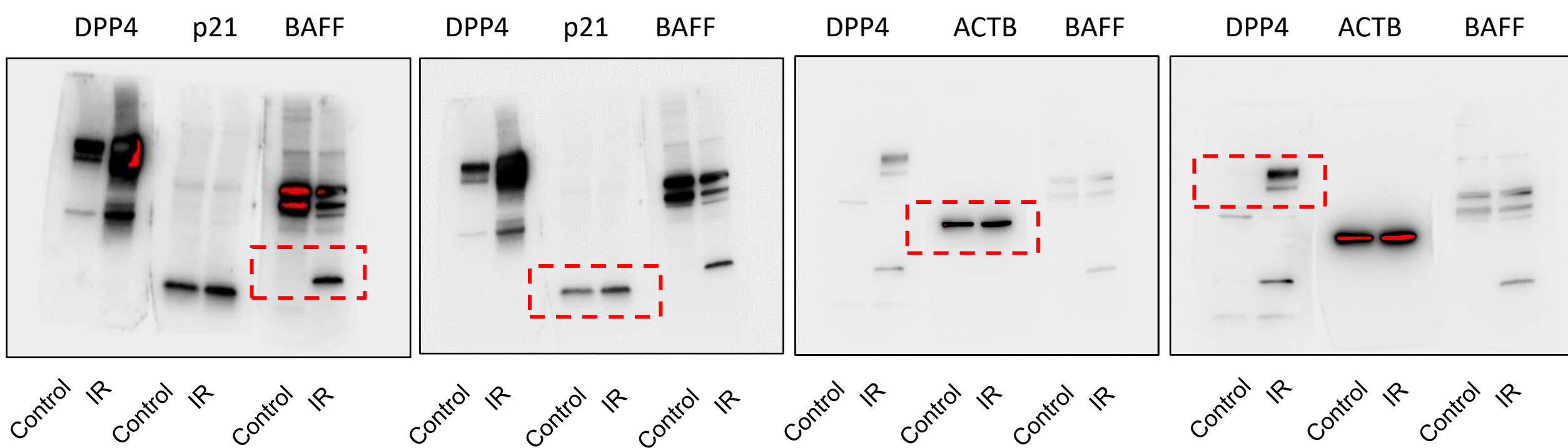**WI-38 OIS RASG12V**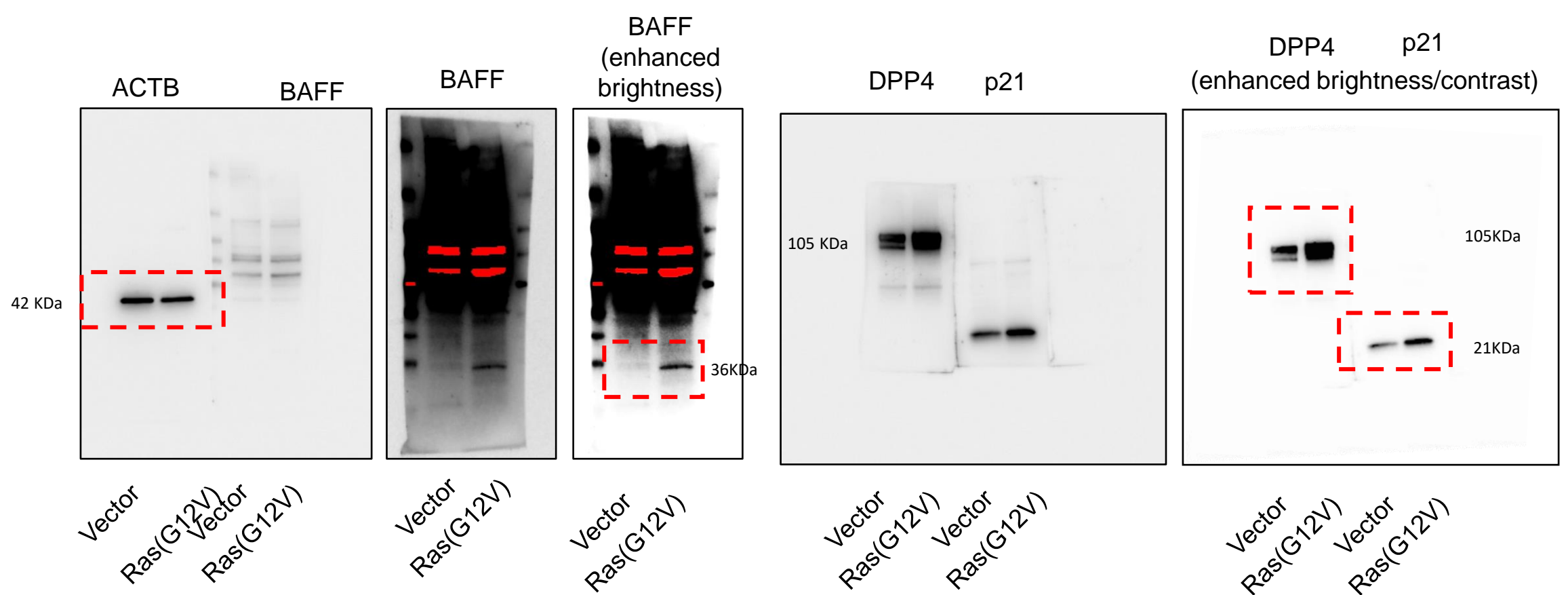

Spleen

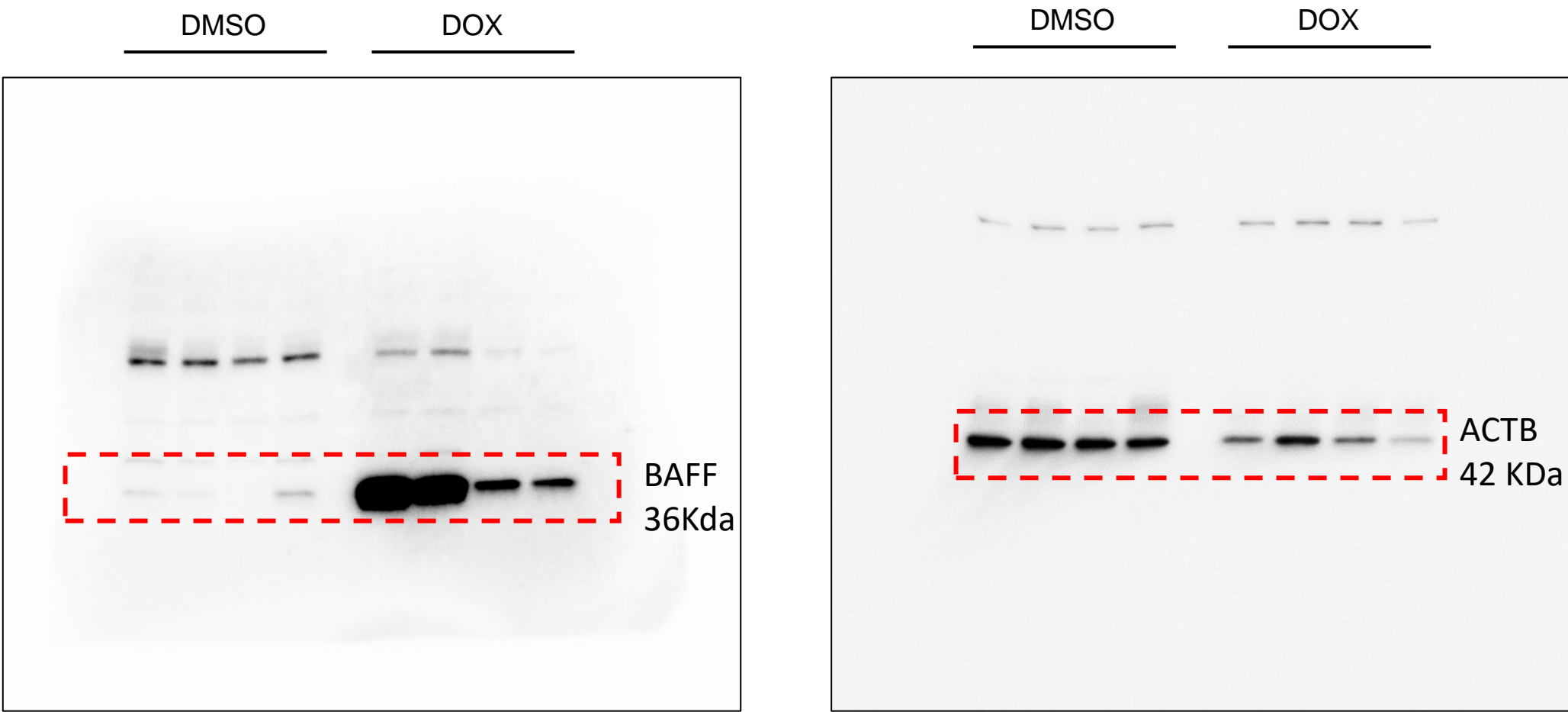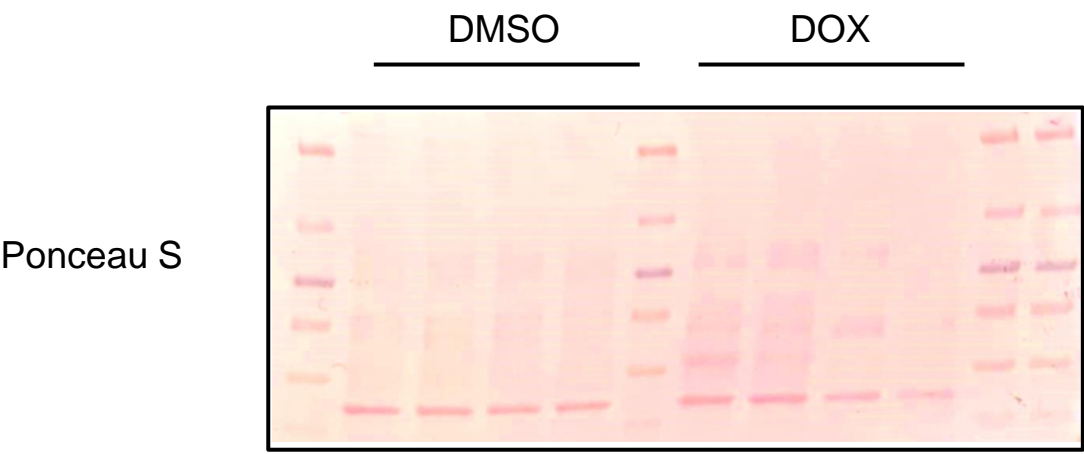

Supplement: Figure 1—source data 1. [file elife-84238-fig1-data1.zip › z Figure 1-Source Data 1/Figure 1-Source Data 1/Figure1-uncropped blots labeled.pdf]

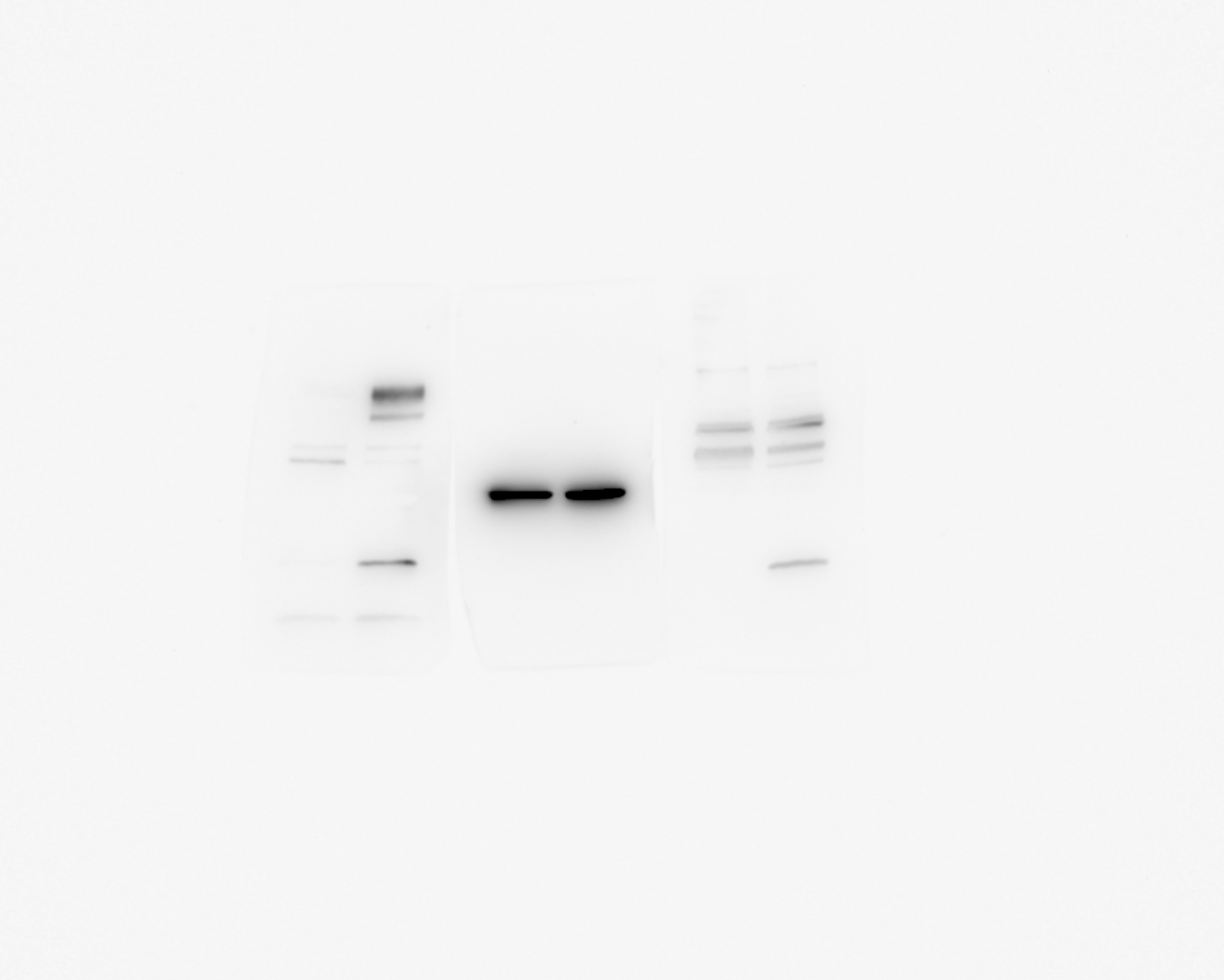

Supplement: Figure 1—source data 1. [file elife-84238-fig1-data1.zip › z Figure 1-Source Data 1/Figure 1-Source Data 1/original files/Fig 1C WI-38 IR/ACTB.tif]

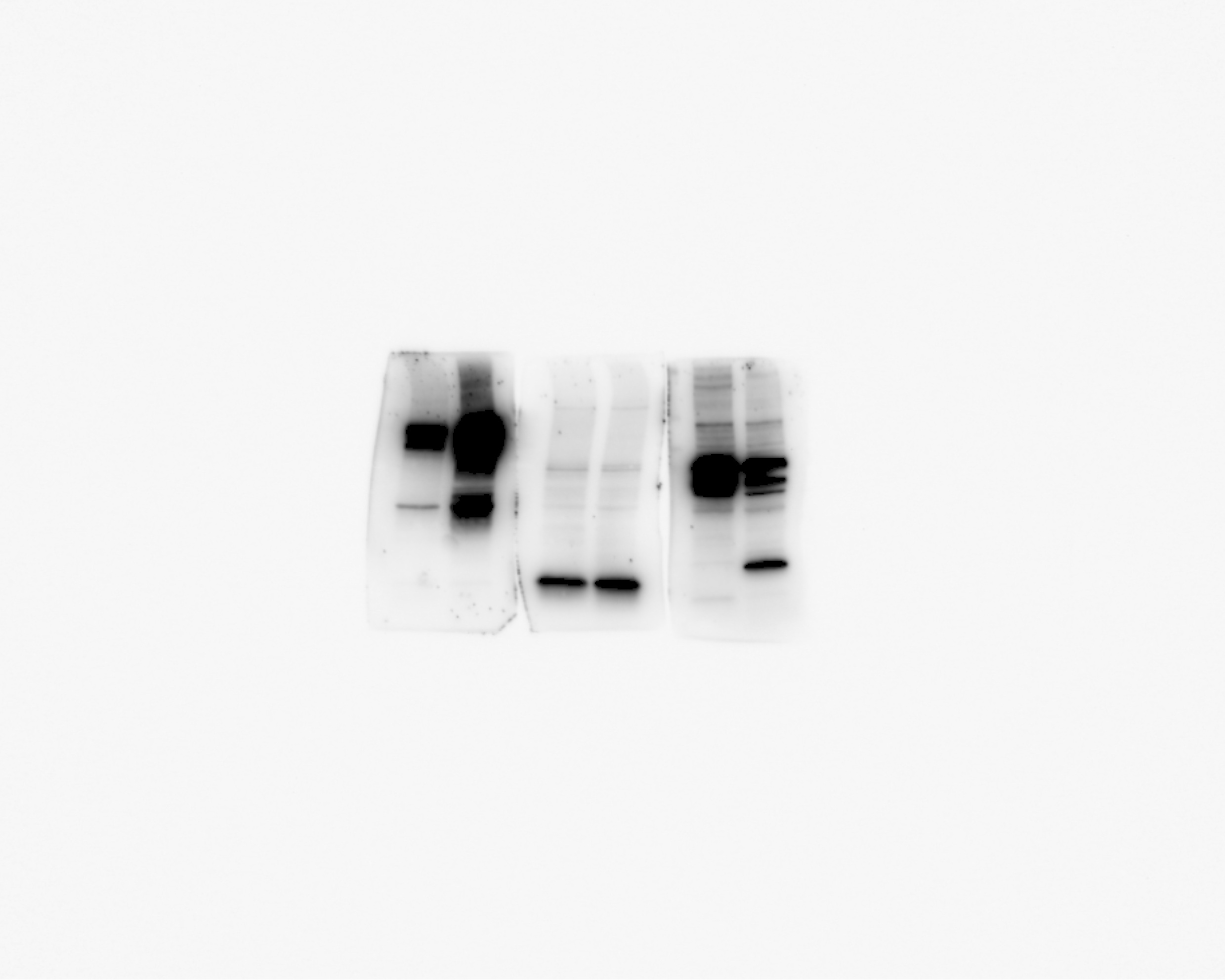

Supplement: Figure 1—source data 1. [file elife-84238-fig1-data1.zip › z Figure 1-Source Data 1/Figure 1-Source Data 1/original files/Fig 1C WI-38 IR/BAFF.jpg]

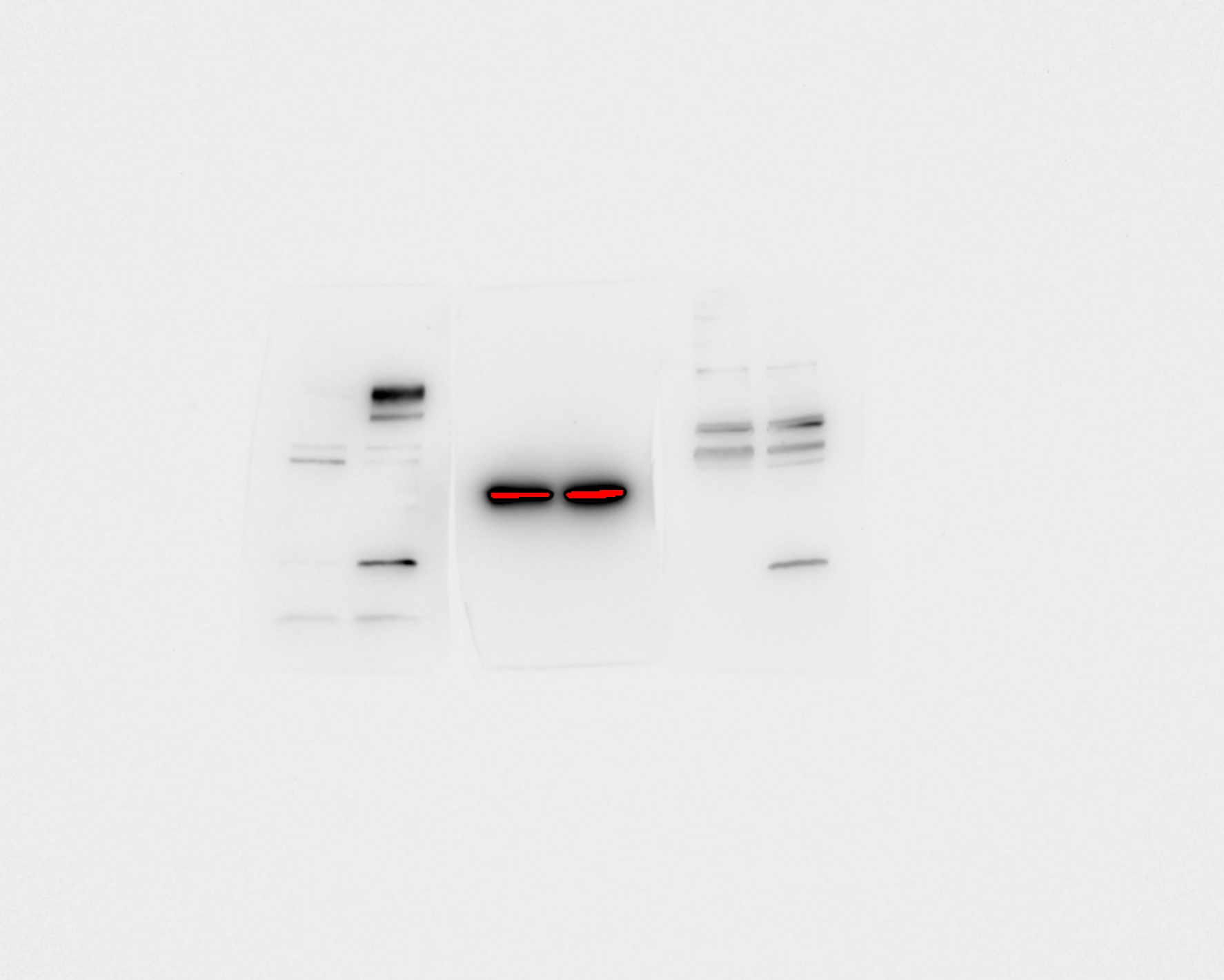

Supplement: Figure 1—source data 1. [file elife-84238-fig1-data1.zip › z Figure 1-Source Data 1/Figure 1-Source Data 1/original files/Fig 1C WI-38 IR/DPP4.jpg]

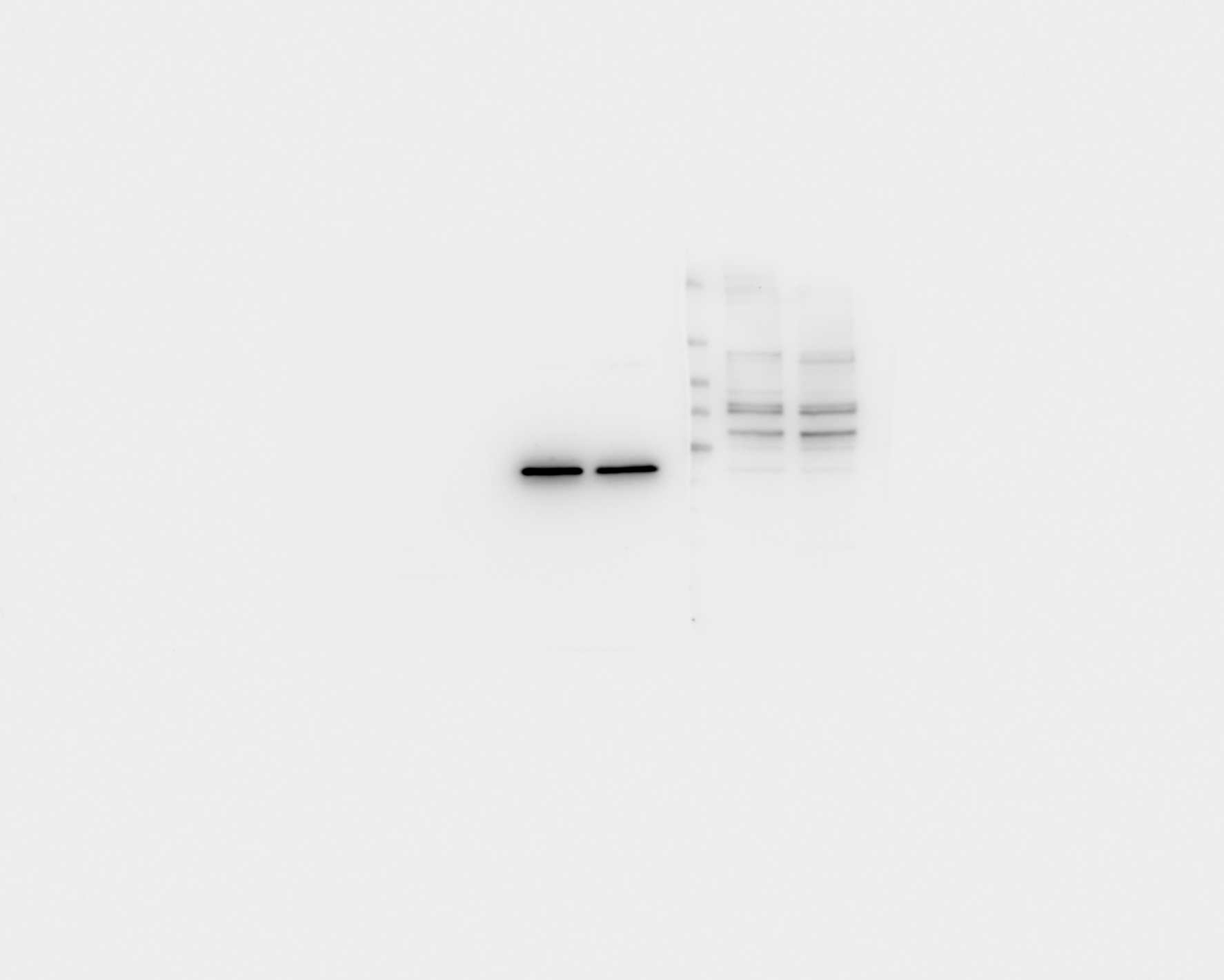

Supplement: Figure 1—source data 1. [file elife-84238-fig1-data1.zip › z Figure 1-Source Data 1/Figure 1-Source Data 1/original files/Fig 1C WI-38 OIS/ACTB.jpg]

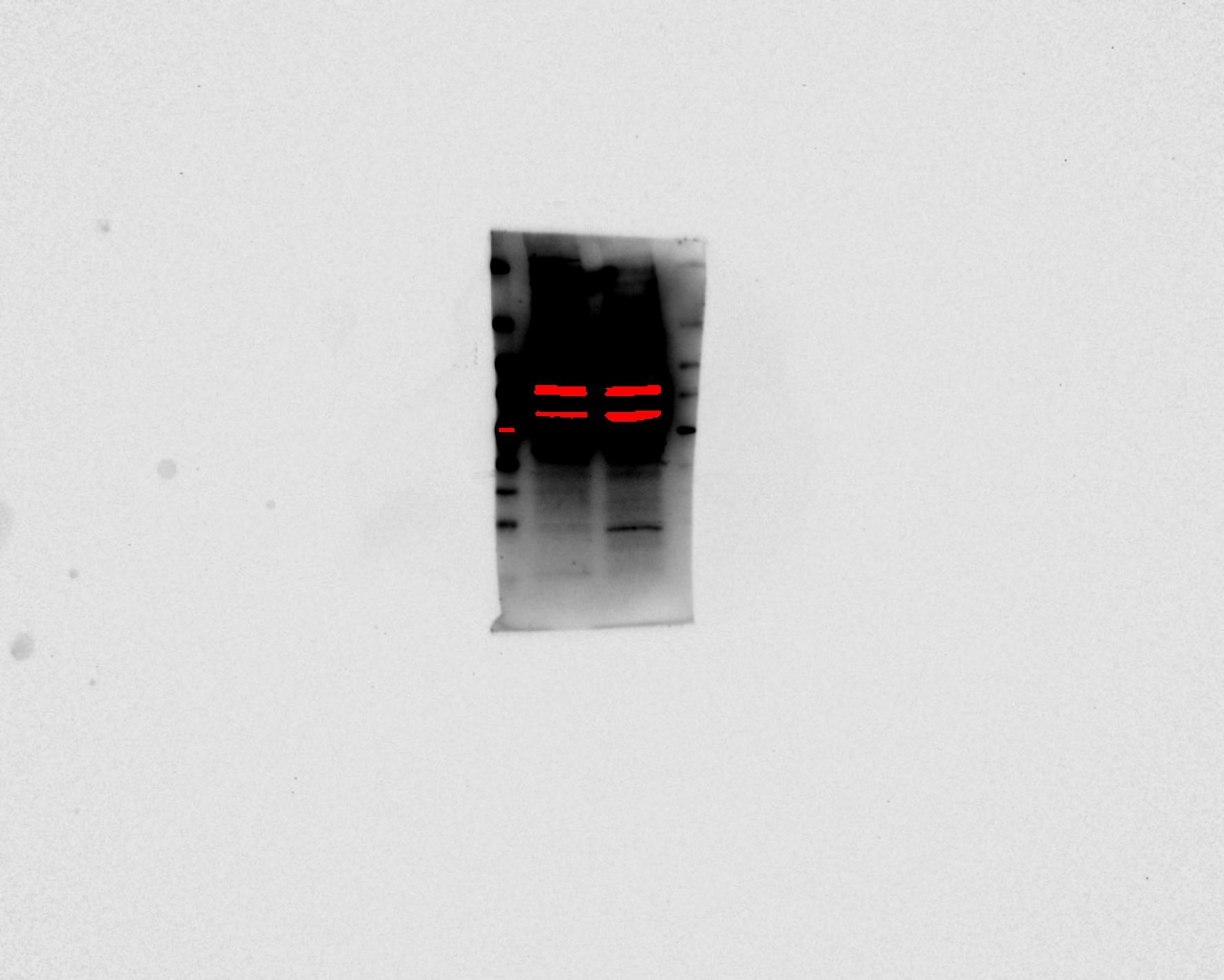

Supplement: Figure 1—source data 1. [file elife-84238-fig1-data1.zip › z Figure 1-Source Data 1/Figure 1-Source Data 1/original files/Fig 1C WI-38 OIS/BAFF.jpg]

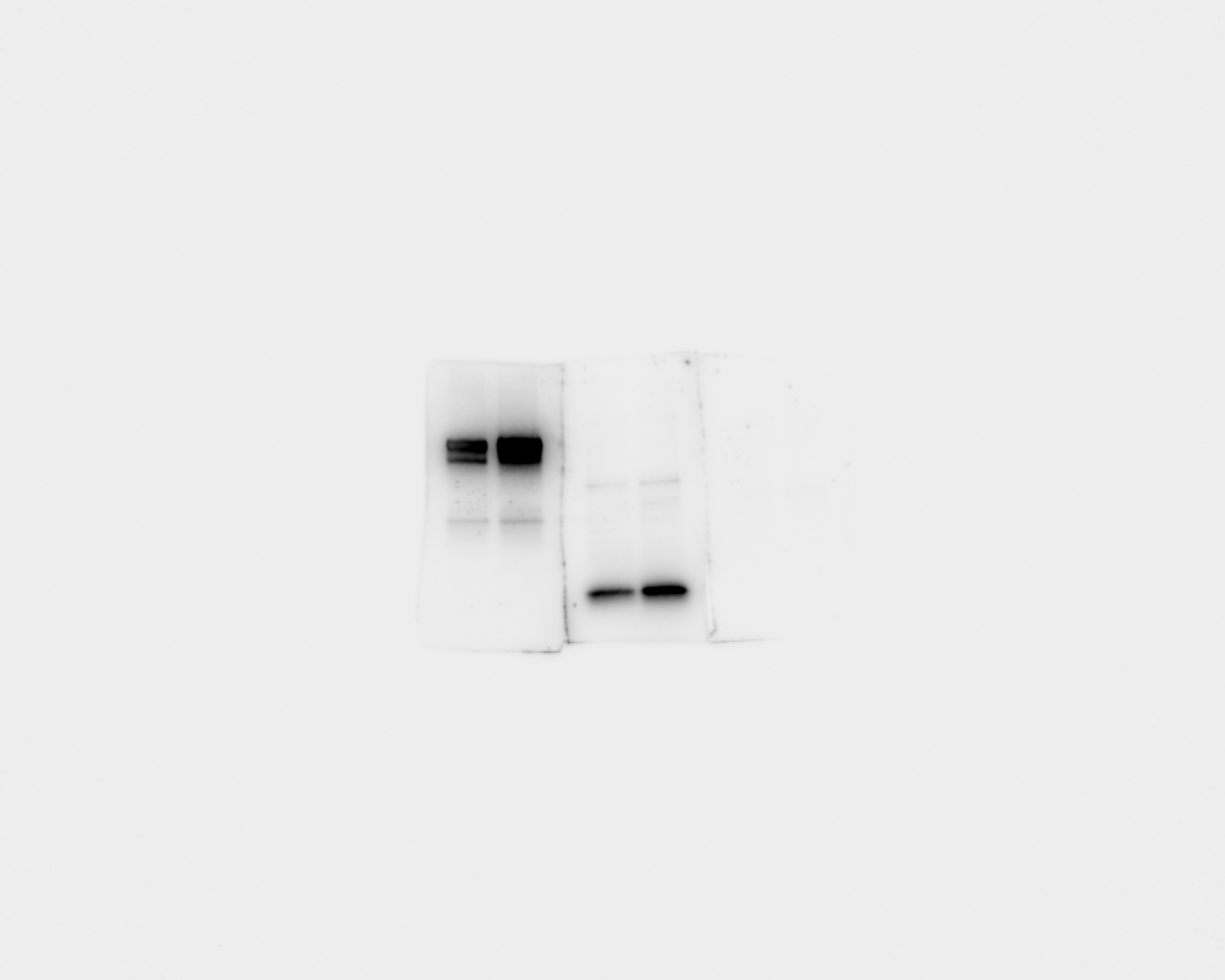

Supplement: Figure 1—source data 1. [file elife-84238-fig1-data1.zip › z Figure 1-Source Data 1/Figure 1-Source Data 1/original files/Fig 1C WI-38 OIS/p21&DPP4 I.jpg]

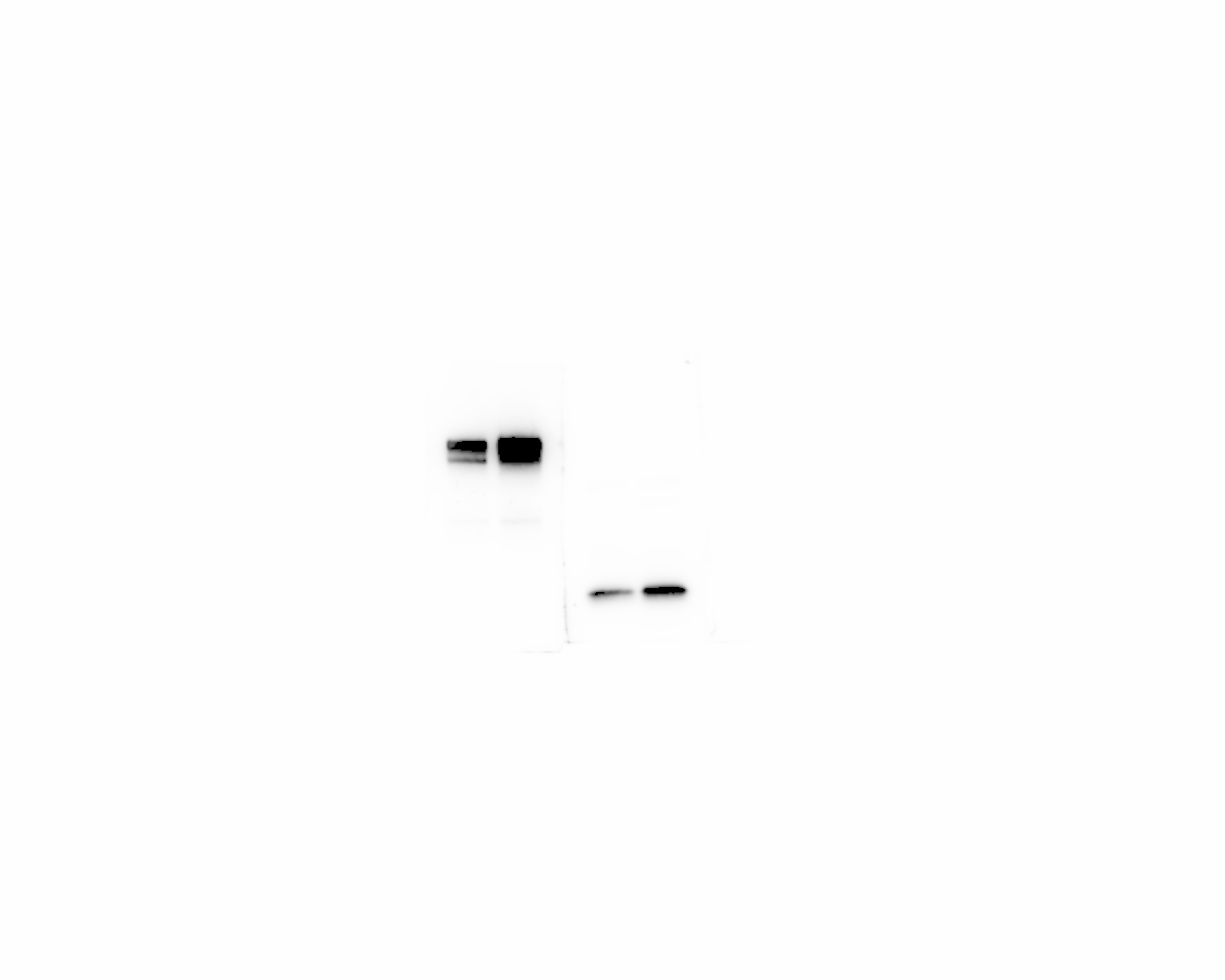

Supplement: Figure 1—source data 1. [file elife-84238-fig1-data1.zip › z Figure 1-Source Data 1/Figure 1-Source Data 1/original files/Fig 1C WI-38 OIS/p21&DPP4 II.jpg]

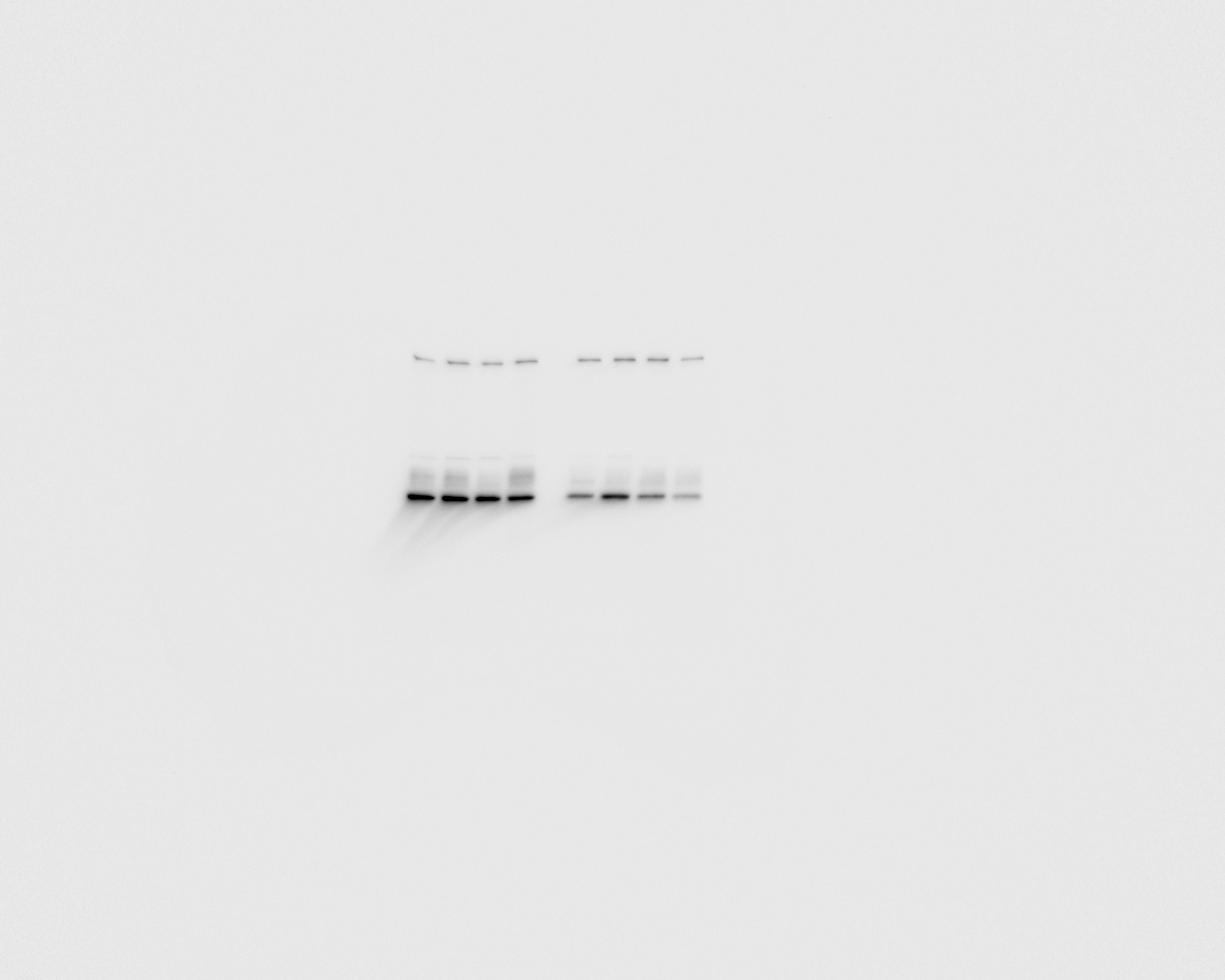

Supplement: Figure 1—source data 1. [file elife-84238-fig1-data1.zip › z Figure 1-Source Data 1/Figure 1-Source Data 1/original files/Fig 1H/ACTB.jpg]

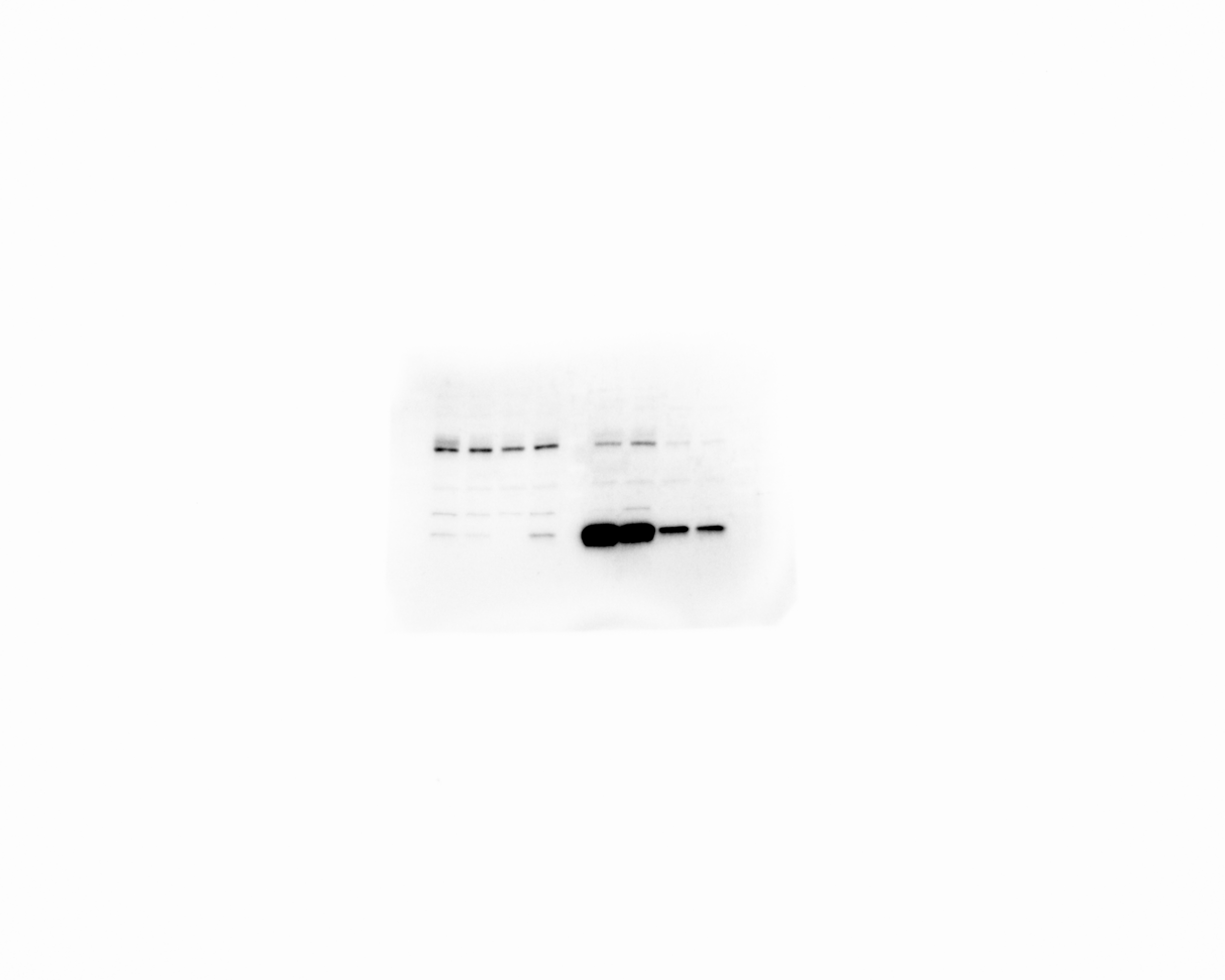

Supplement: Figure 1—source data 1. [file elife-84238-fig1-data1.zip › z Figure 1-Source Data 1/Figure 1-Source Data 1/original files/Fig 1H/BAFF.jpg]

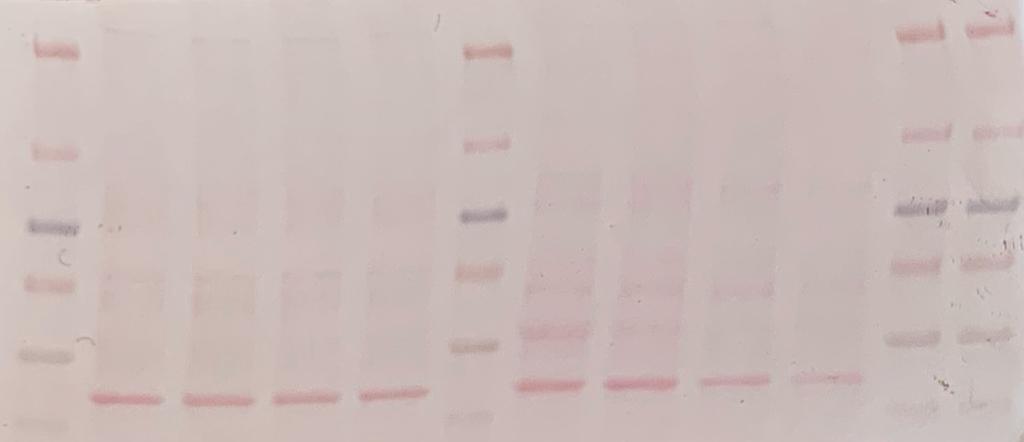

Supplement: Figure 1—source data 1. [file elife-84238-fig1-data1.zip › z Figure 1-Source Data 1/Figure 1-Source Data 1/original files/Fig 1H/Ponceau S.jpg]

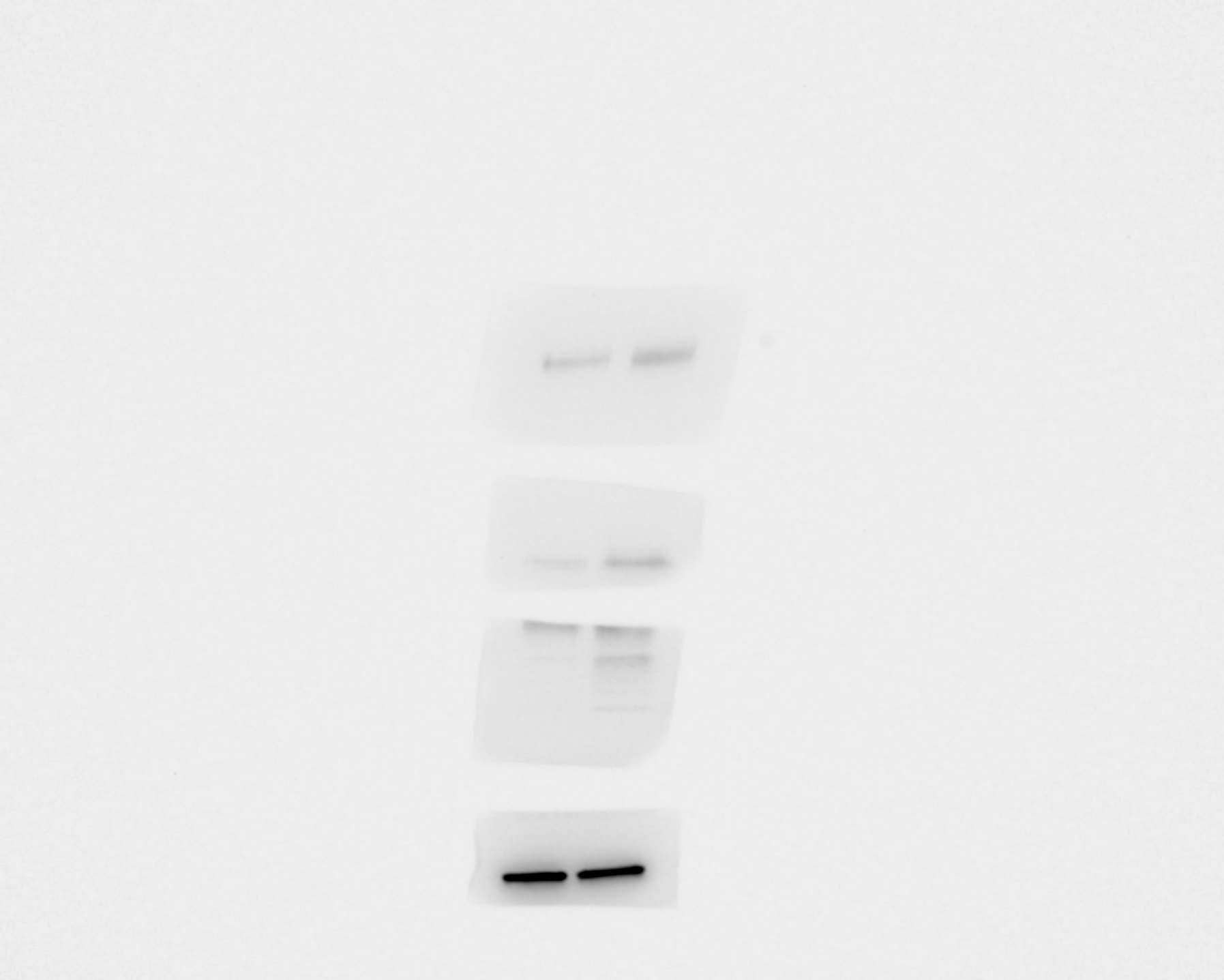

Supplement: Figure 1—source data 1. [file elife-84238-fig1-data1.zip › z Figure 1-Source Data 1/Figure 1-Source Data 1/original files/Fig1C THP-1 DOX/ACTB unlabeled.jpg]

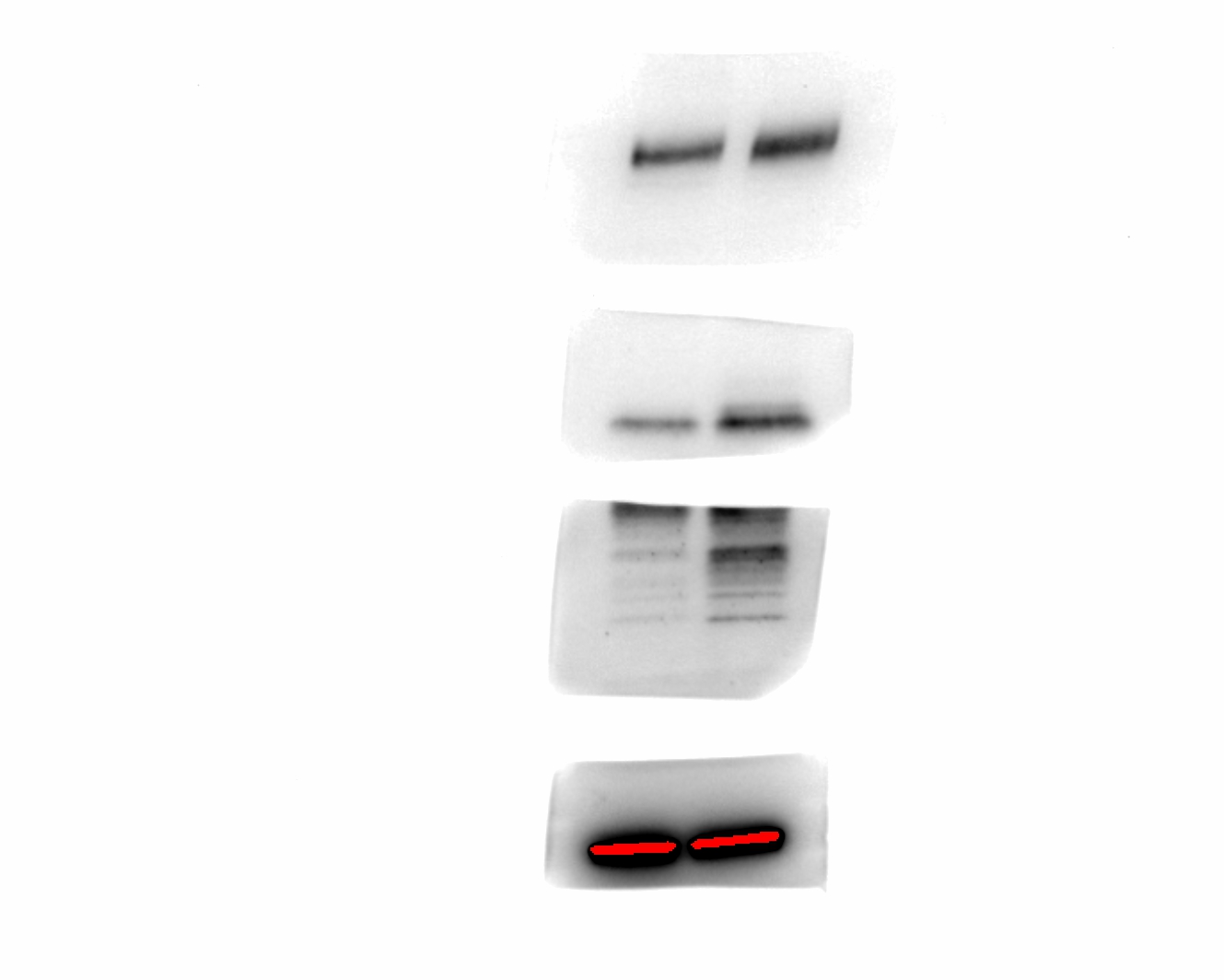

Supplement: Figure 1—source data 1. [file elife-84238-fig1-data1.zip › z Figure 1-Source Data 1/Figure 1-Source Data 1/original files/Fig1C THP-1 DOX/BAFF,p21,DPP4 unlabeled.jpg]

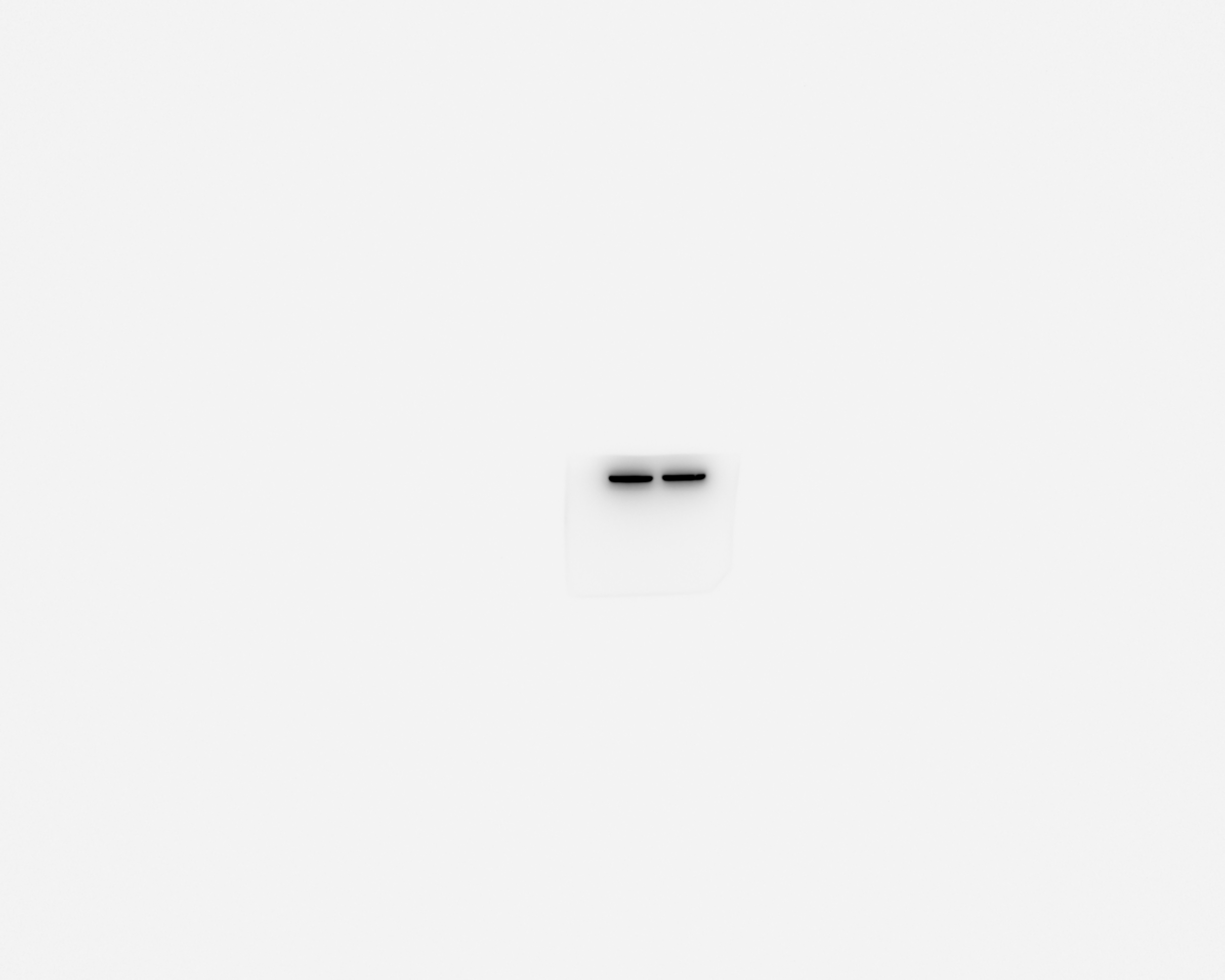

Supplement: Figure 1—source data 1. [file elife-84238-fig1-data1.zip › z Figure 1-Source Data 1/Figure 1-Source Data 1/original files/Fig1C THP-1 IR/ACTB.jpg]

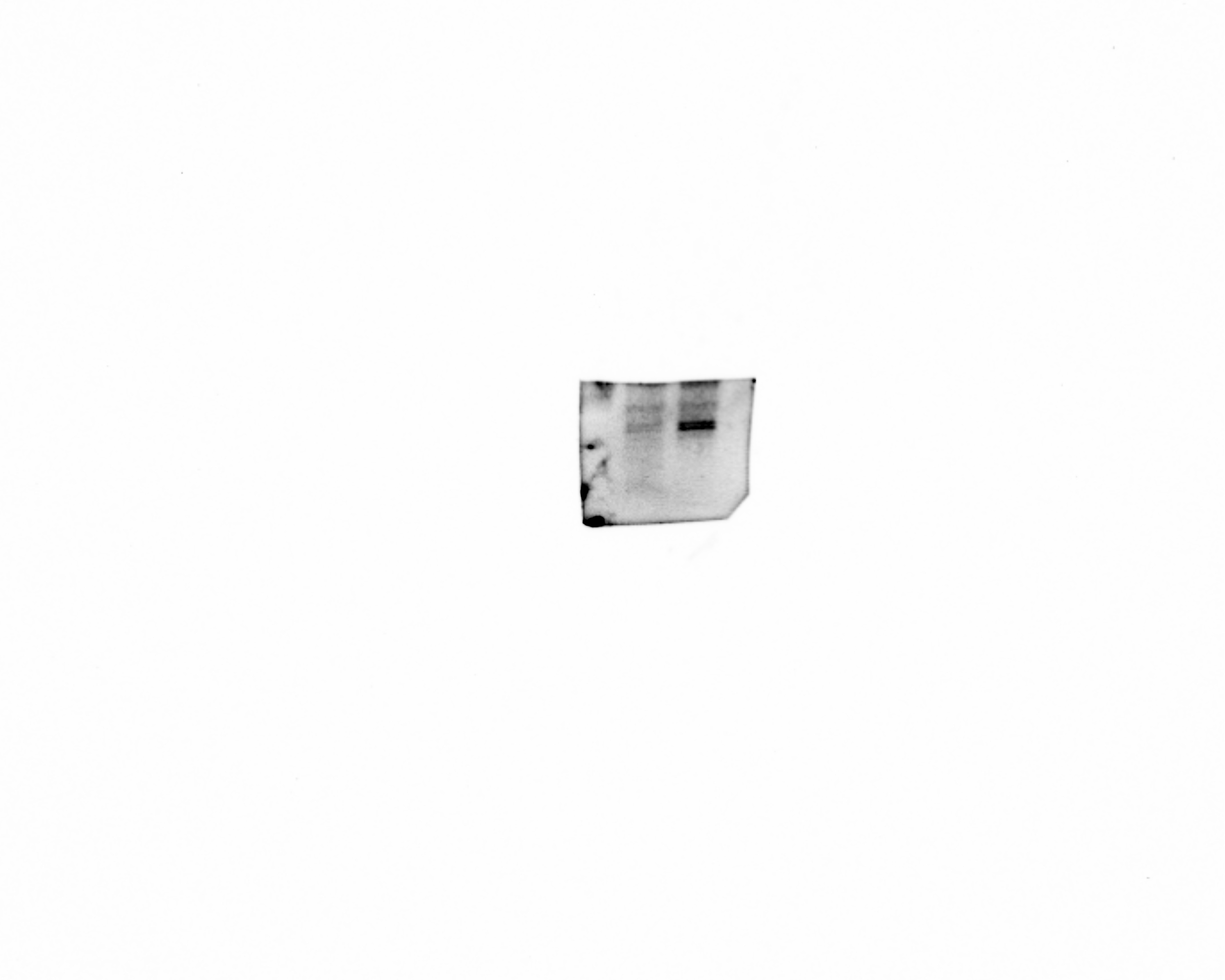

Supplement: Figure 1—source data 1. [file elife-84238-fig1-data1.zip › z Figure 1-Source Data 1/Figure 1-Source Data 1/original files/Fig1C THP-1 IR/BAFF.jpg]

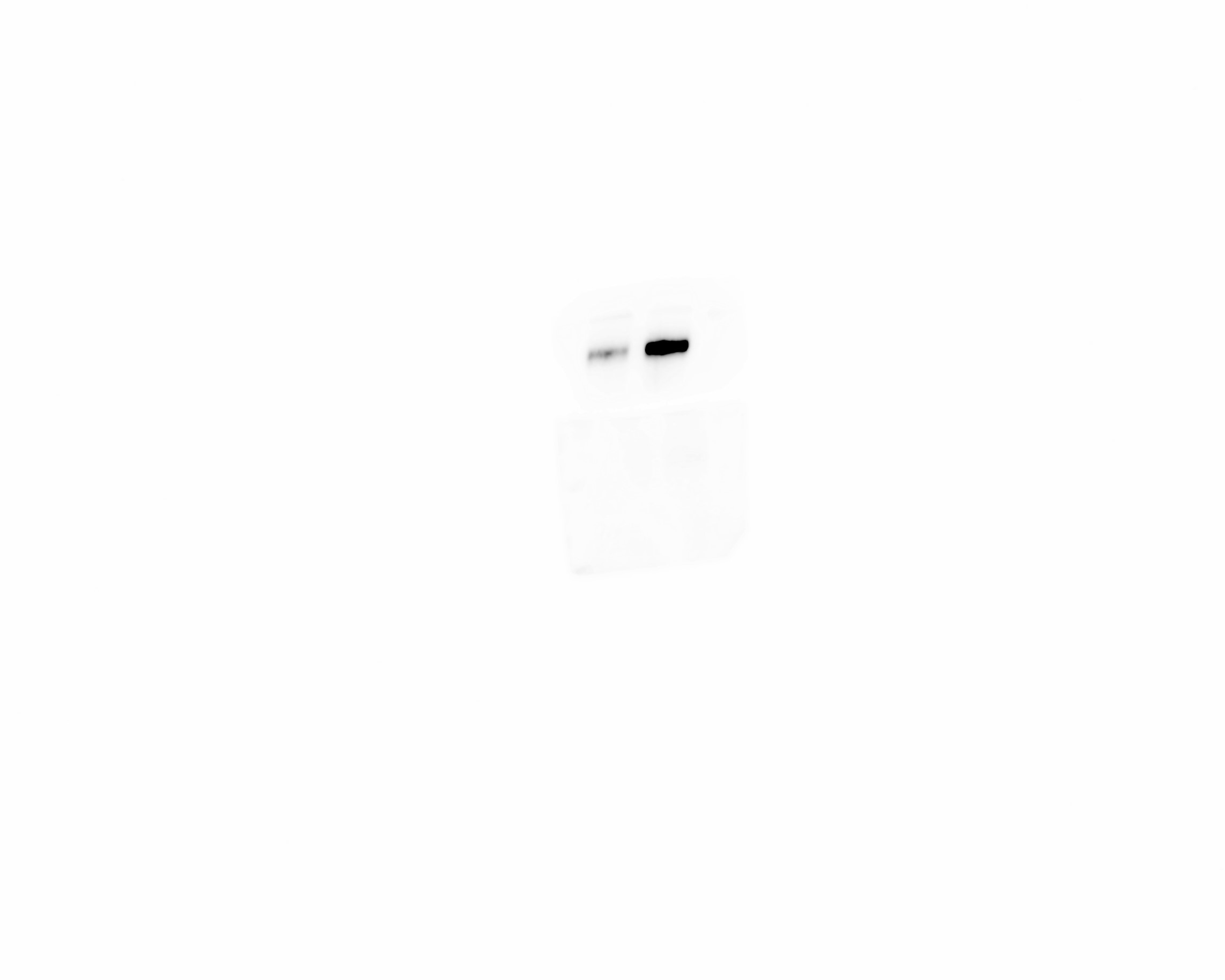

Supplement: Figure 1—source data 1. [file elife-84238-fig1-data1.zip › z Figure 1-Source Data 1/Figure 1-Source Data 1/original files/Fig1C THP-1 IR/DPP4.jpg]

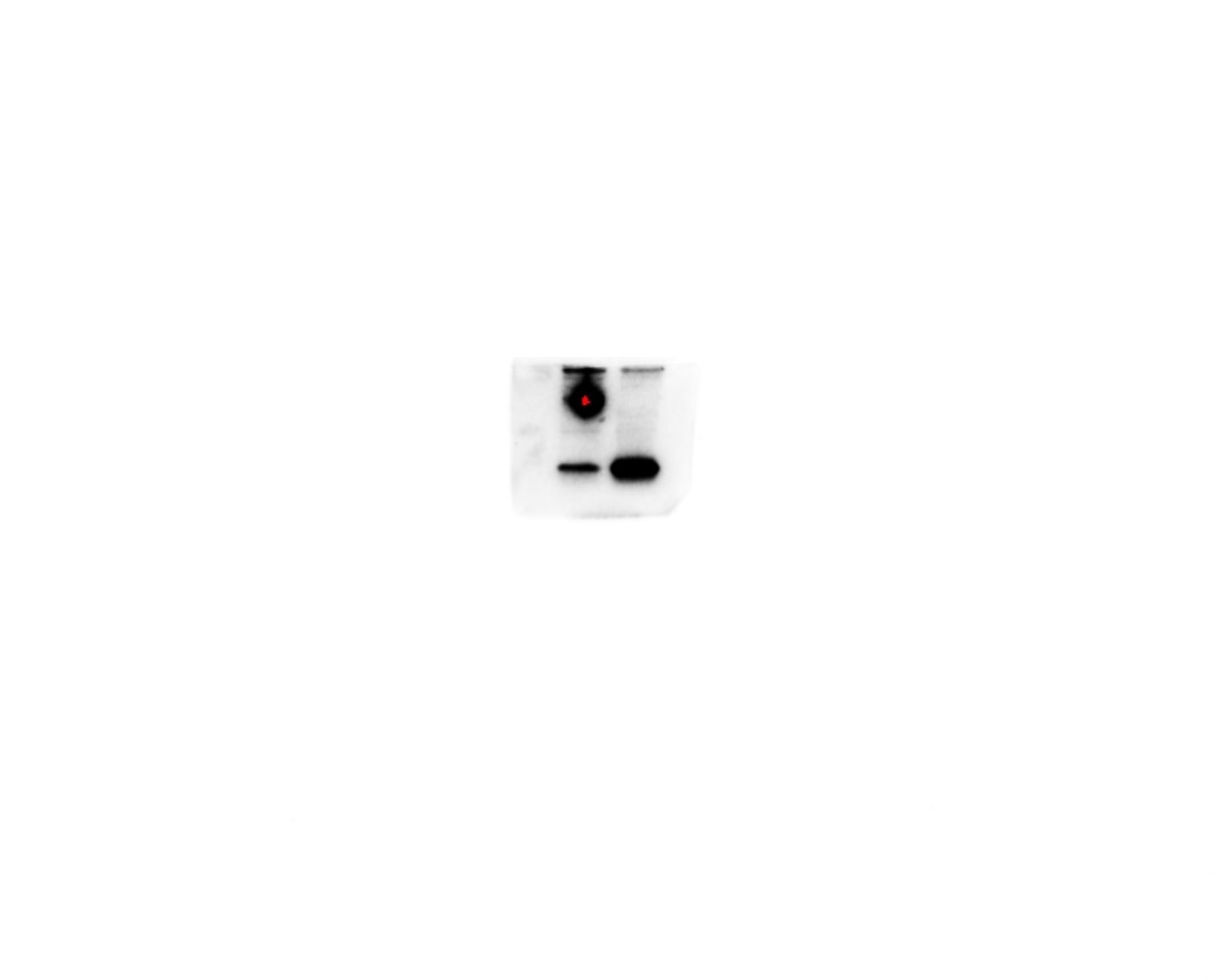

Supplement: Figure 1—source data 1. [file elife-84238-fig1-data1.zip › z Figure 1-Source Data 1/Figure 1-Source Data 1/original files/Fig1C THP-1 IR/p21.jpg]

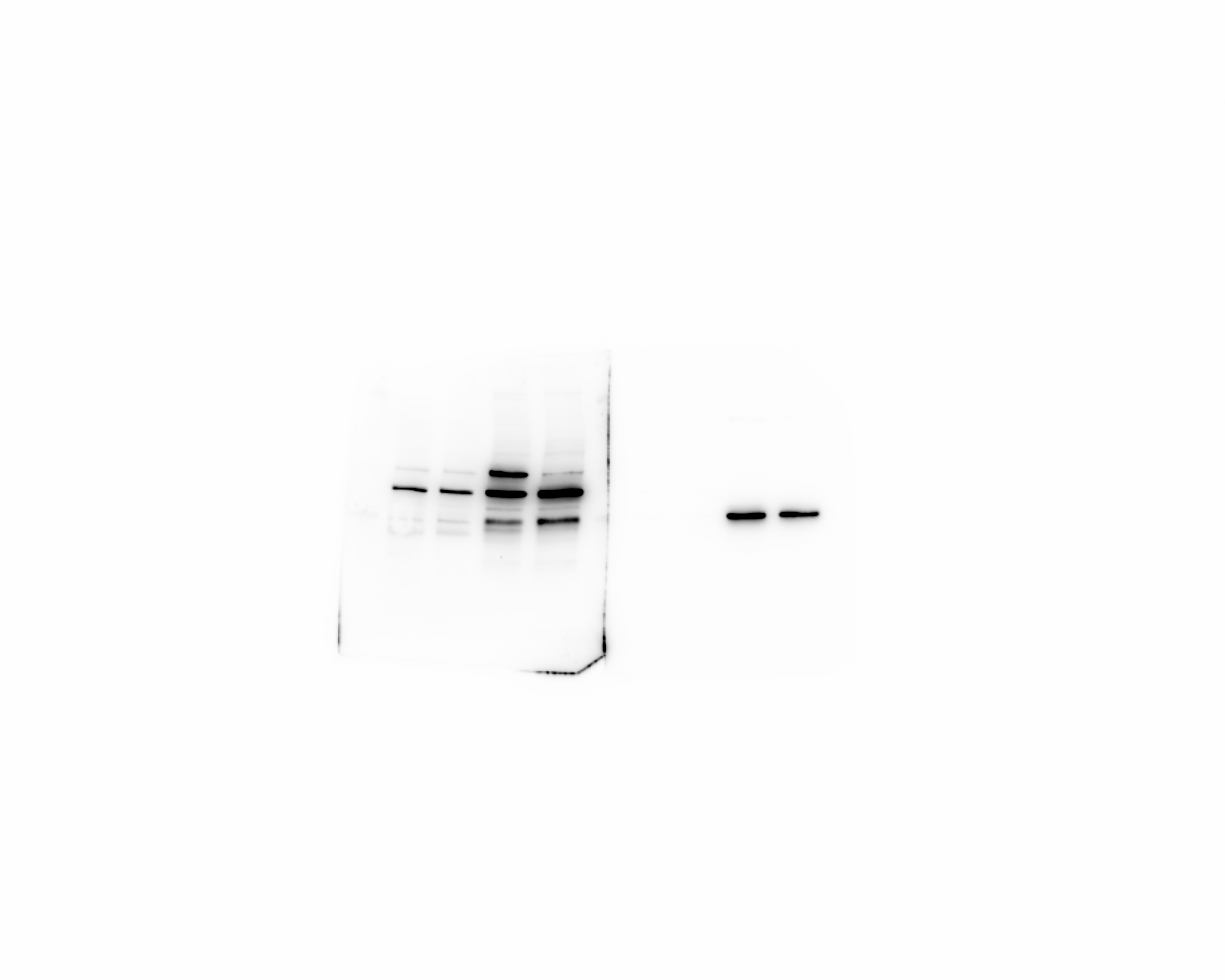

Supplement: Figure 2—source data 2. [file elife-84238-fig2-data2.zip › z Figure 2-Source Data 2/Figure 2-Source Data 2/original files/IRF1&IRF2.jpg]

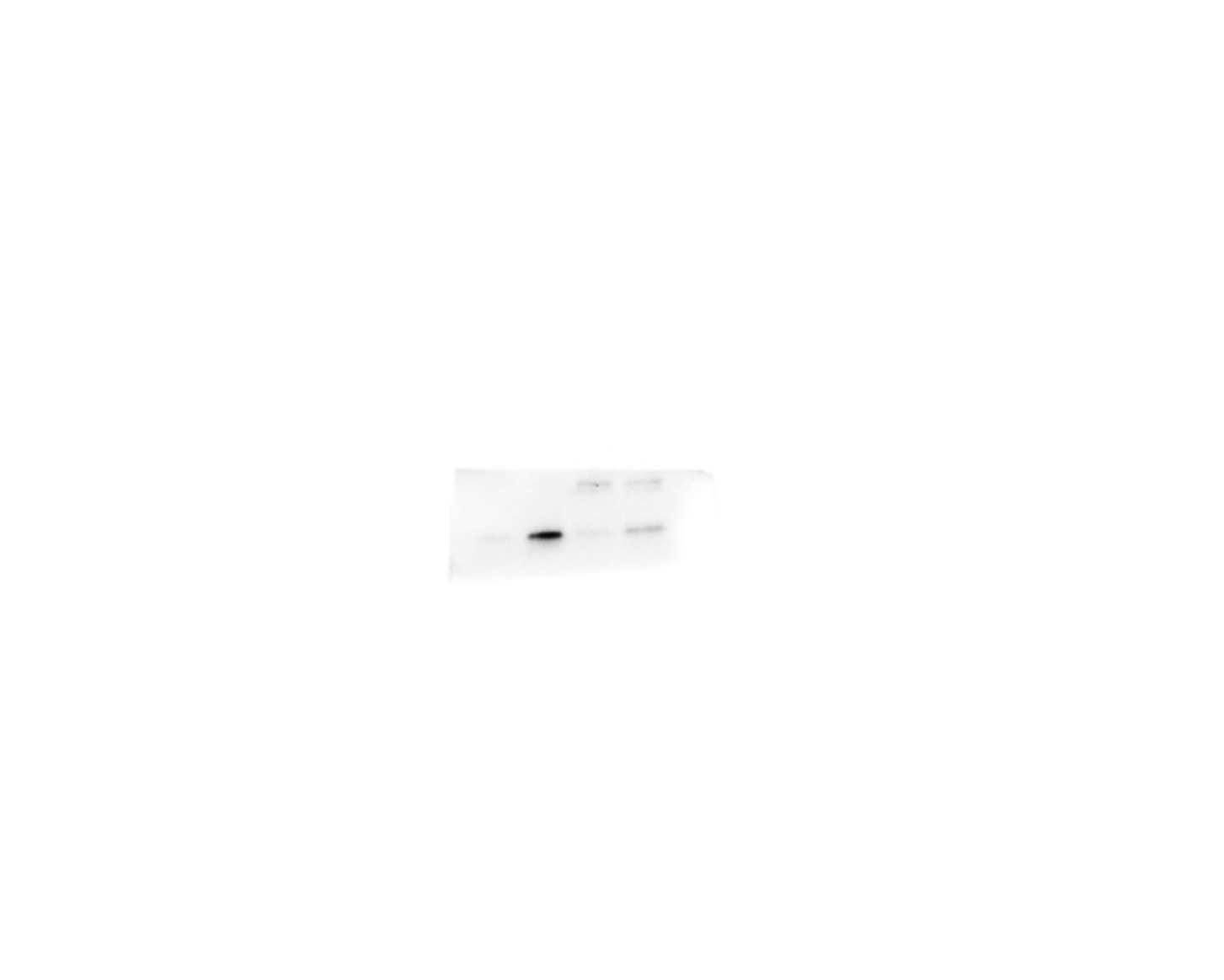

Supplement: Figure 2—source data 2. [file elife-84238-fig2-data2.zip › z Figure 2-Source Data 2/Figure 2-Source Data 2/original files/p21.jpg]

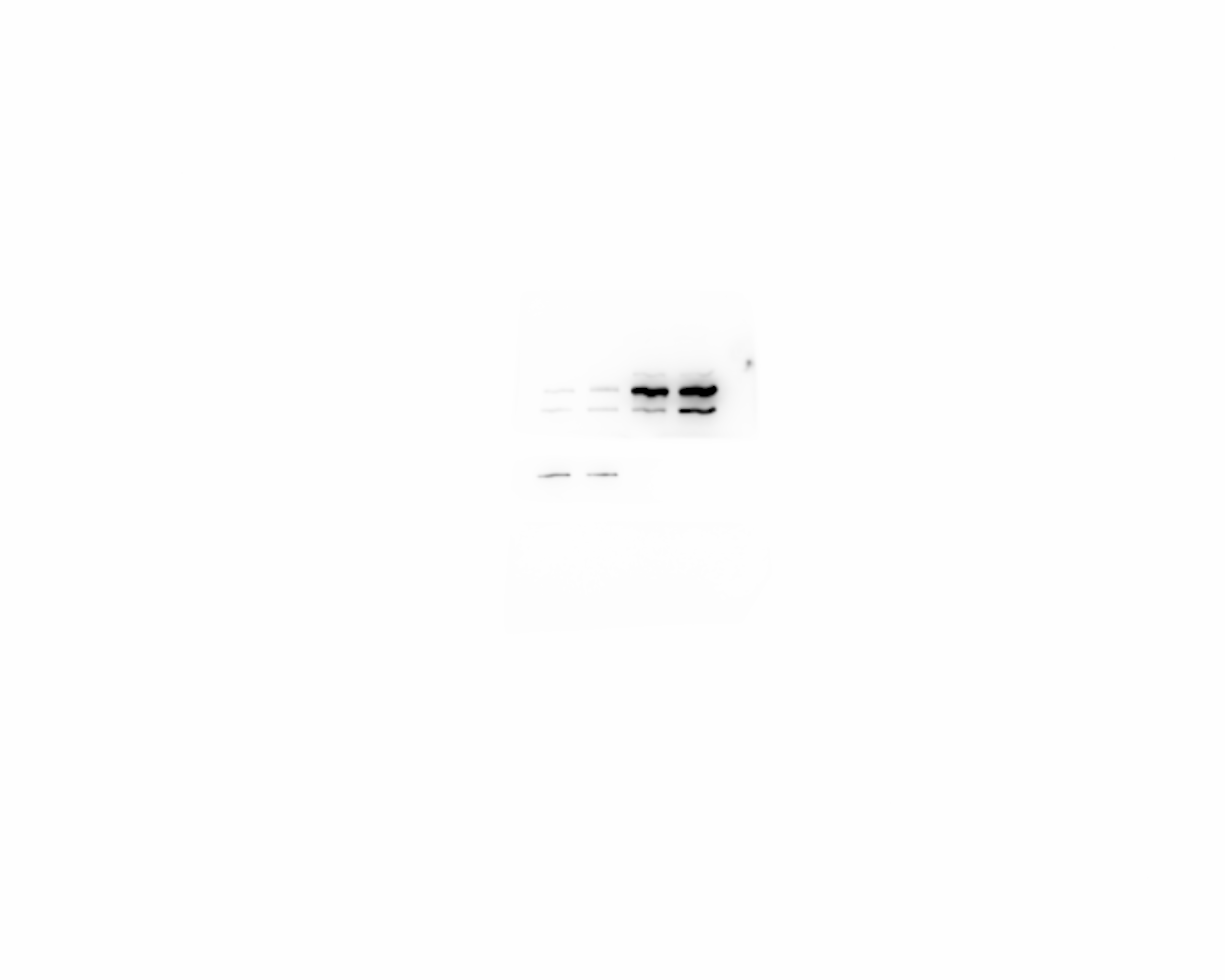

Supplement: Figure 2—source data 2. [file elife-84238-fig2-data2.zip › z Figure 2-Source Data 2/Figure 2-Source Data 2/original files/PARP1.jpg]

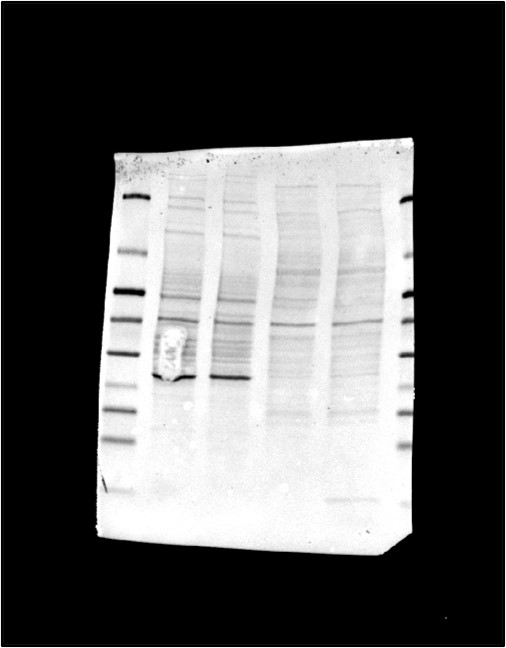

Supplement: Figure 2—source data 2. [file elife-84238-fig2-data2.zip › z Figure 2-Source Data 2/Figure 2-Source Data 2/original files/Ponceau S.jpg]

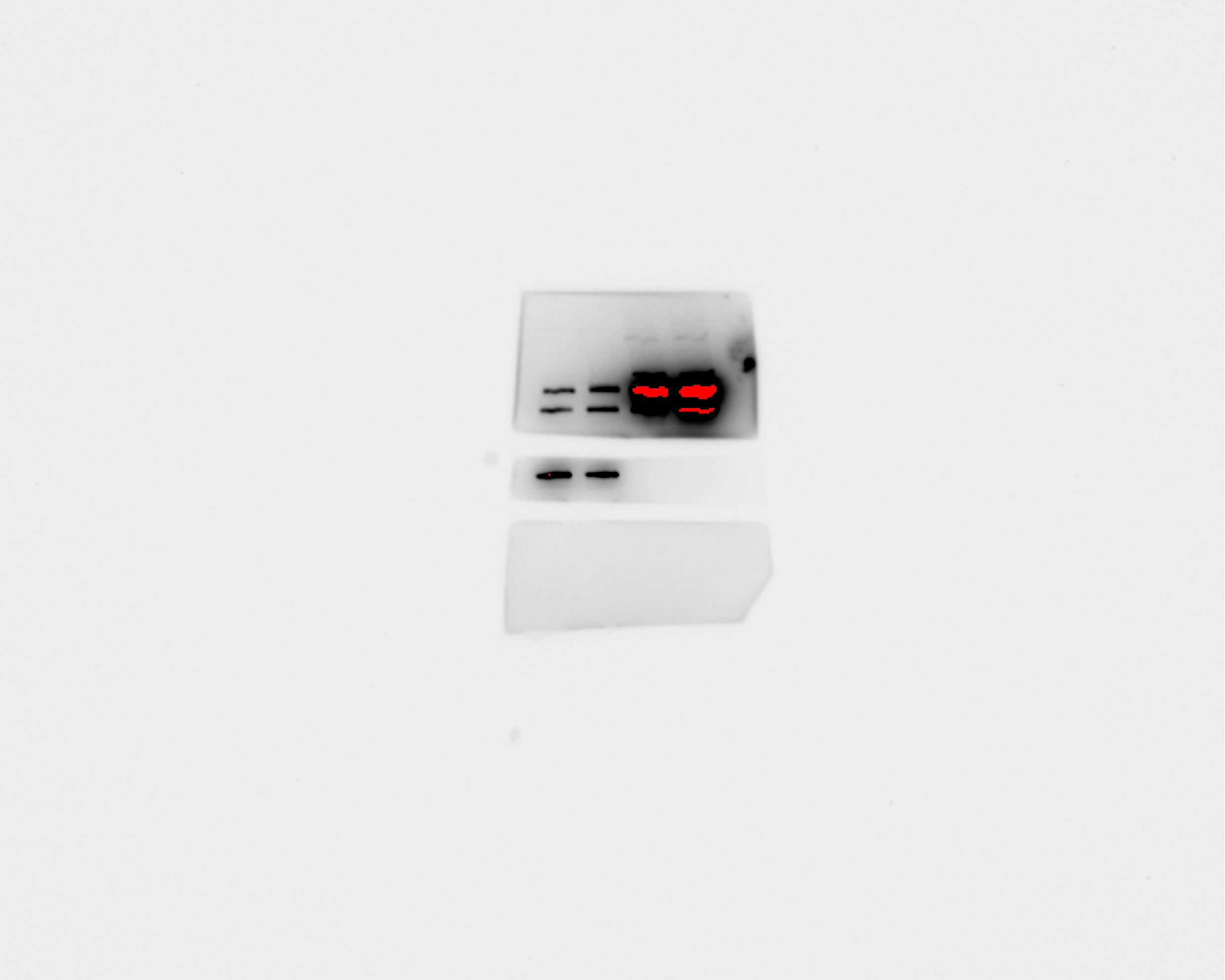

Supplement: Figure 2—source data 2. [file elife-84238-fig2-data2.zip › z Figure 2-Source Data 2/Figure 2-Source Data 2/original files/Tubulin.jpg]

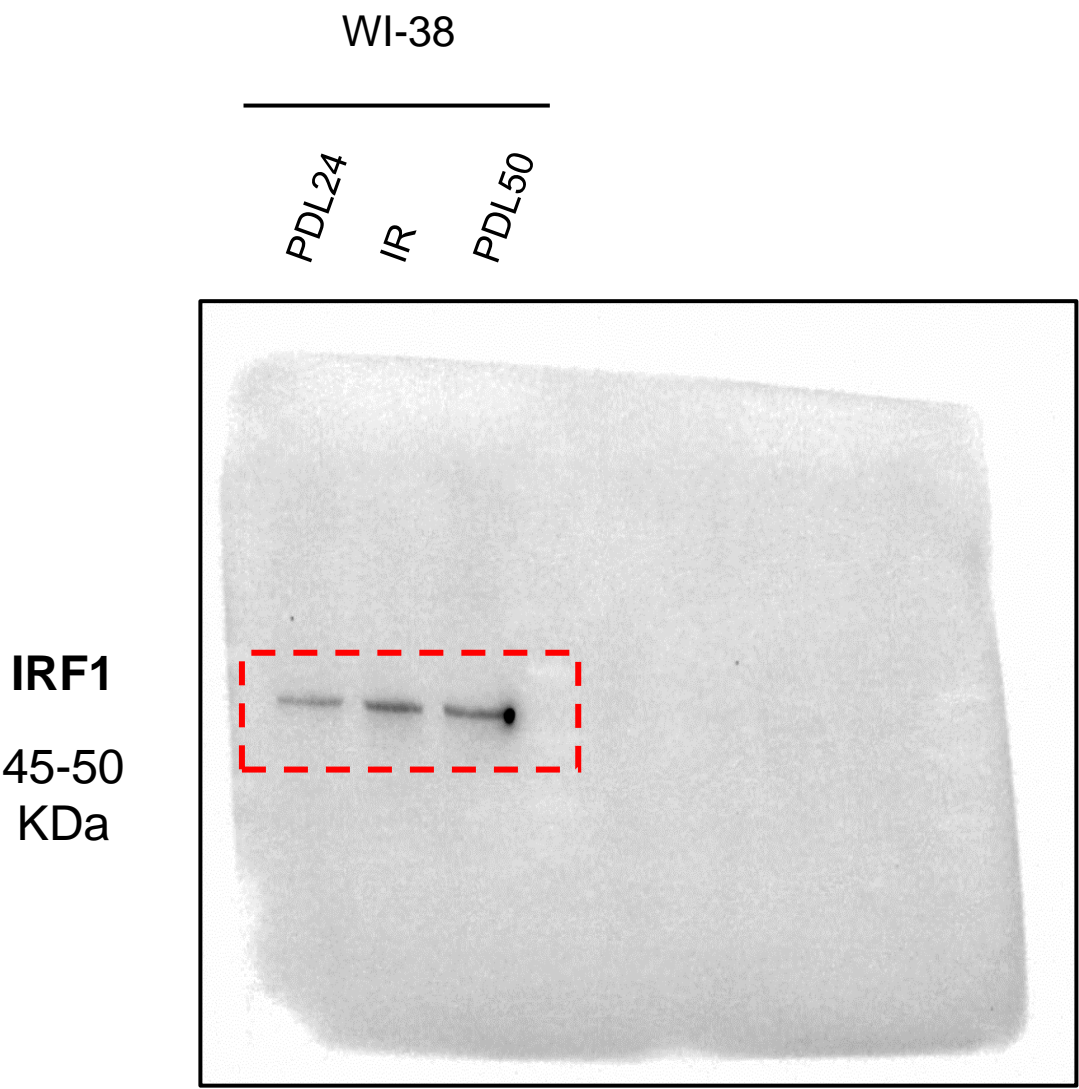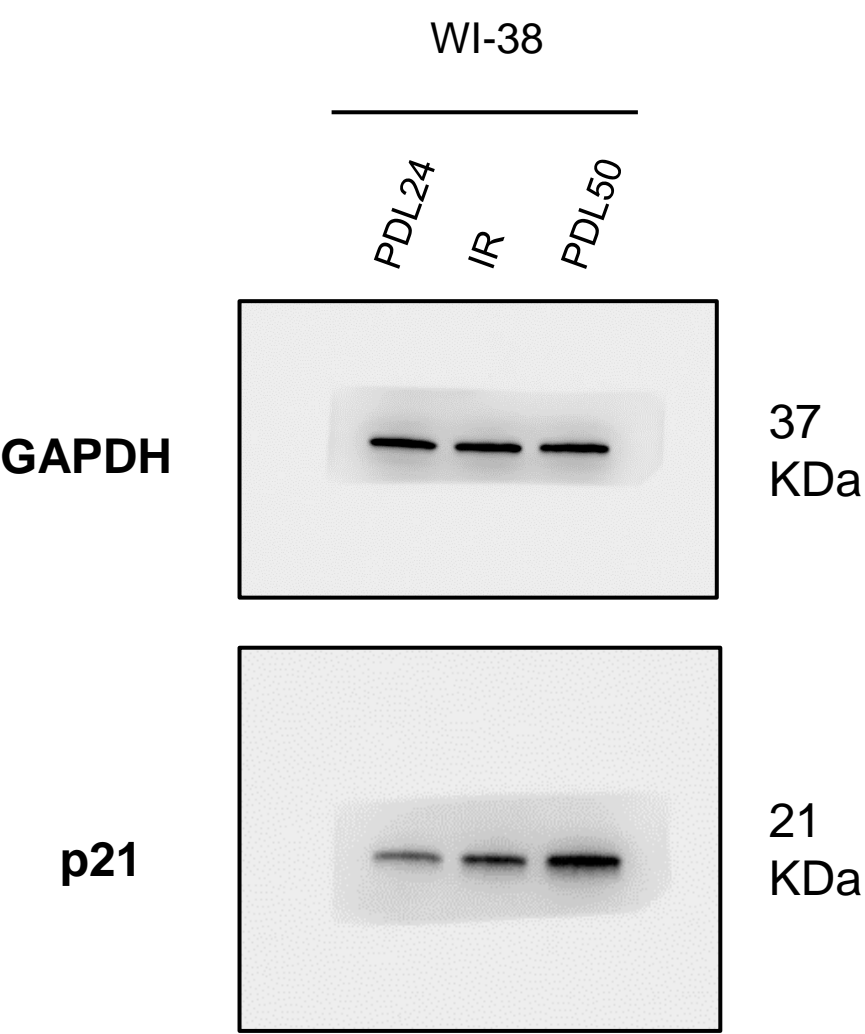

Supplement: Figure 2—figure supplement 1—source data 1. [file elife-84238-fig2-figsupp1-data1.zip › z Figure 2- Figure Supplement 1- Source Data 1/Figure 2- Figure Supplement 1- Source Data 1/Figure S2 uncropped blots labeled.pdf]

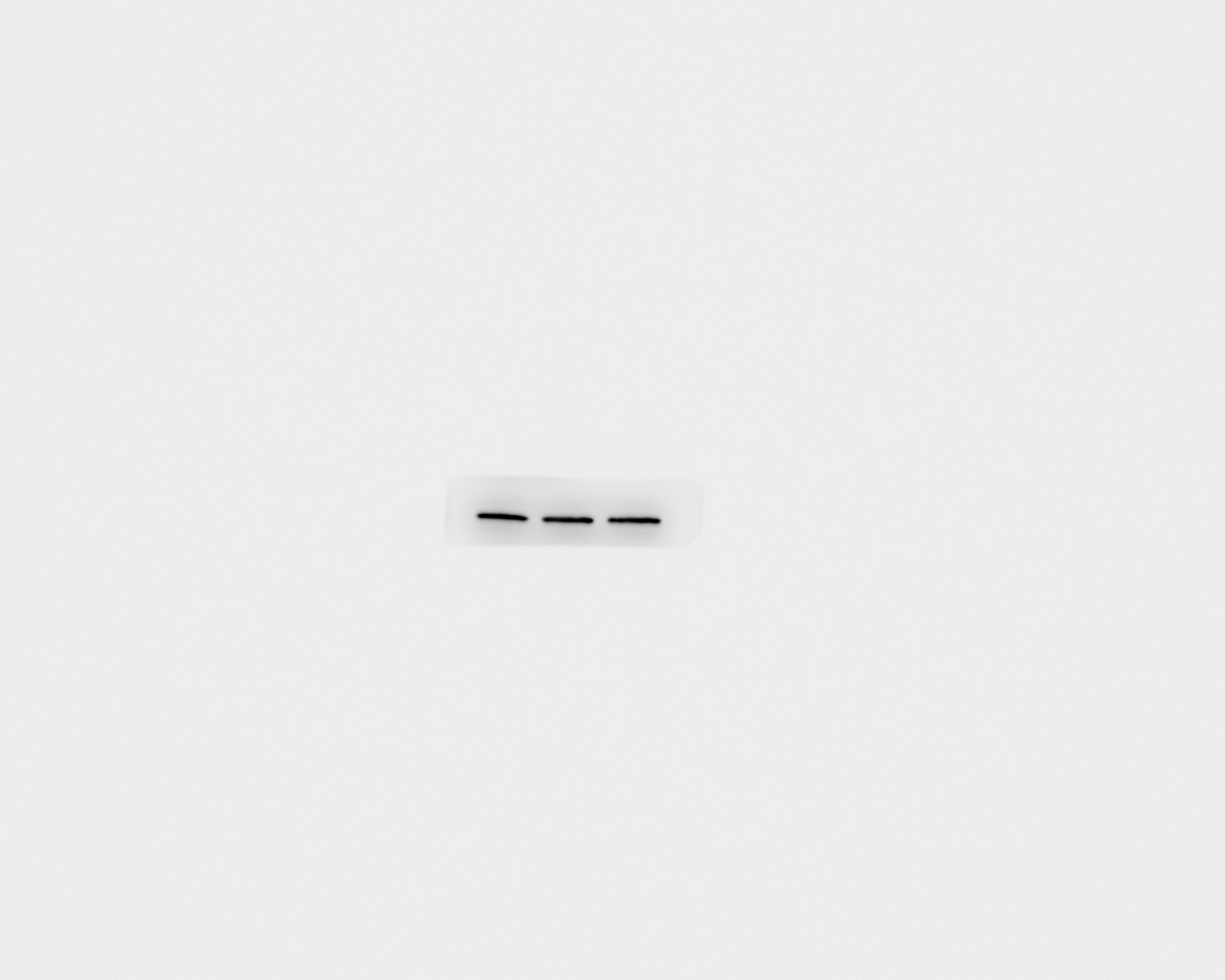

Supplement: Figure 2—figure supplement 1—source data 1. [file elife-84238-fig2-figsupp1-data1.zip › z Figure 2- Figure Supplement 1- Source Data 1/Figure 2- Figure Supplement 1- Source Data 1/original files/GAPDH.jpg]

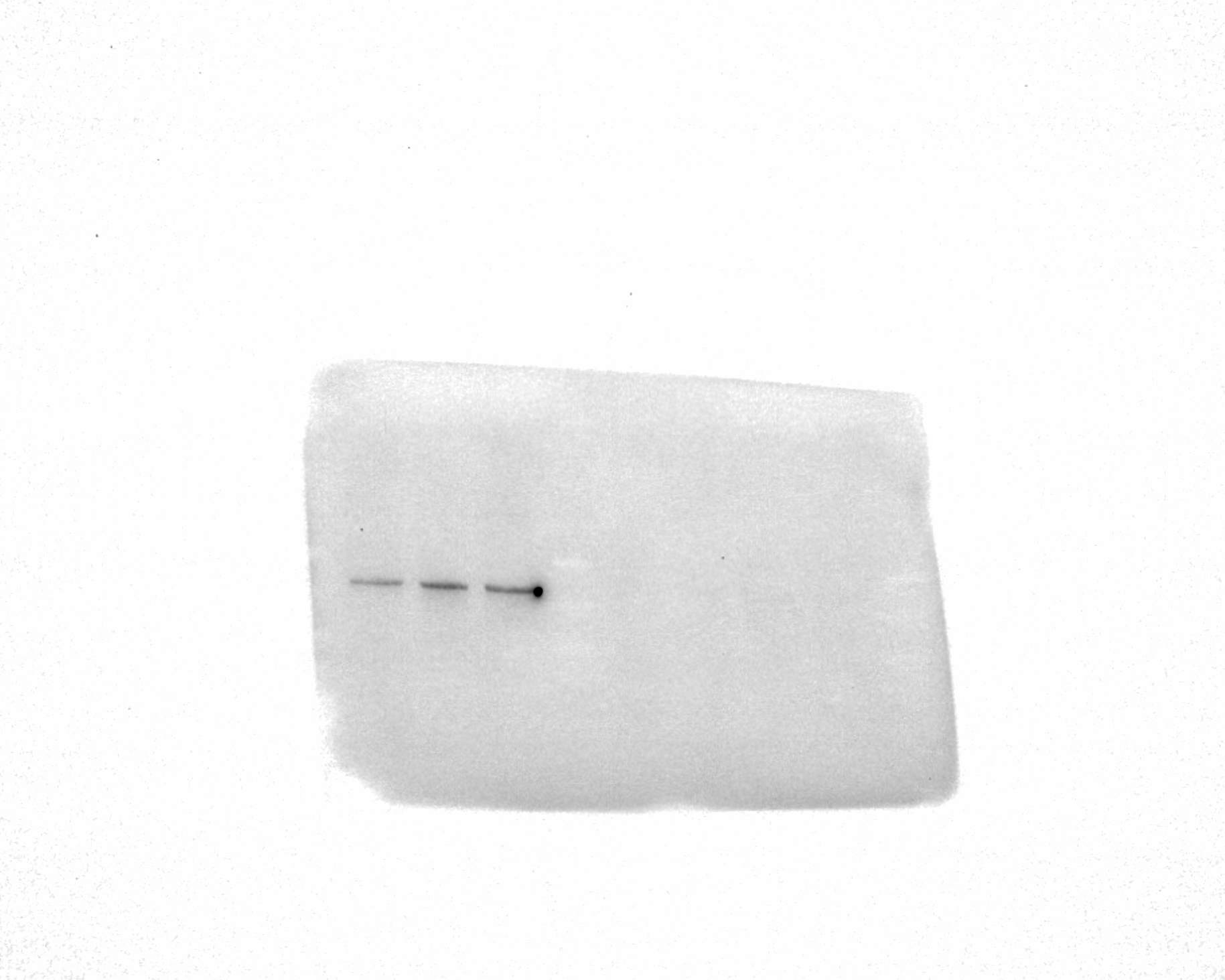

Supplement: Figure 2—figure supplement 1—source data 1. [file elife-84238-fig2-figsupp1-data1.zip › z Figure 2- Figure Supplement 1- Source Data 1/Figure 2- Figure Supplement 1- Source Data 1/original files/IRF1 .jpg]

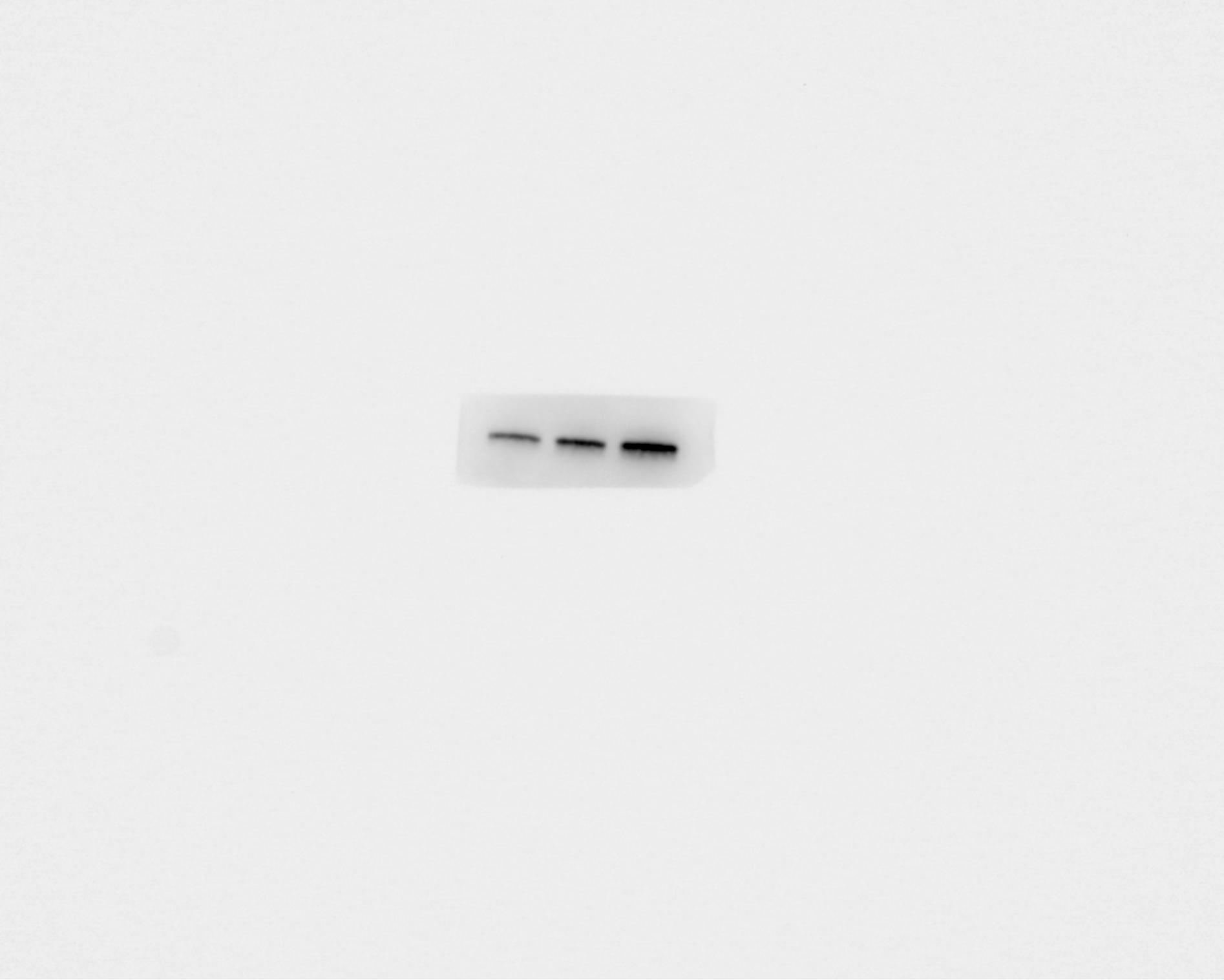

Supplement: Figure 2—figure supplement 1—source data 1. [file elife-84238-fig2-figsupp1-data1.zip › z Figure 2- Figure Supplement 1- Source Data 1/Figure 2- Figure Supplement 1- Source Data 1/original files/p21.jpg]

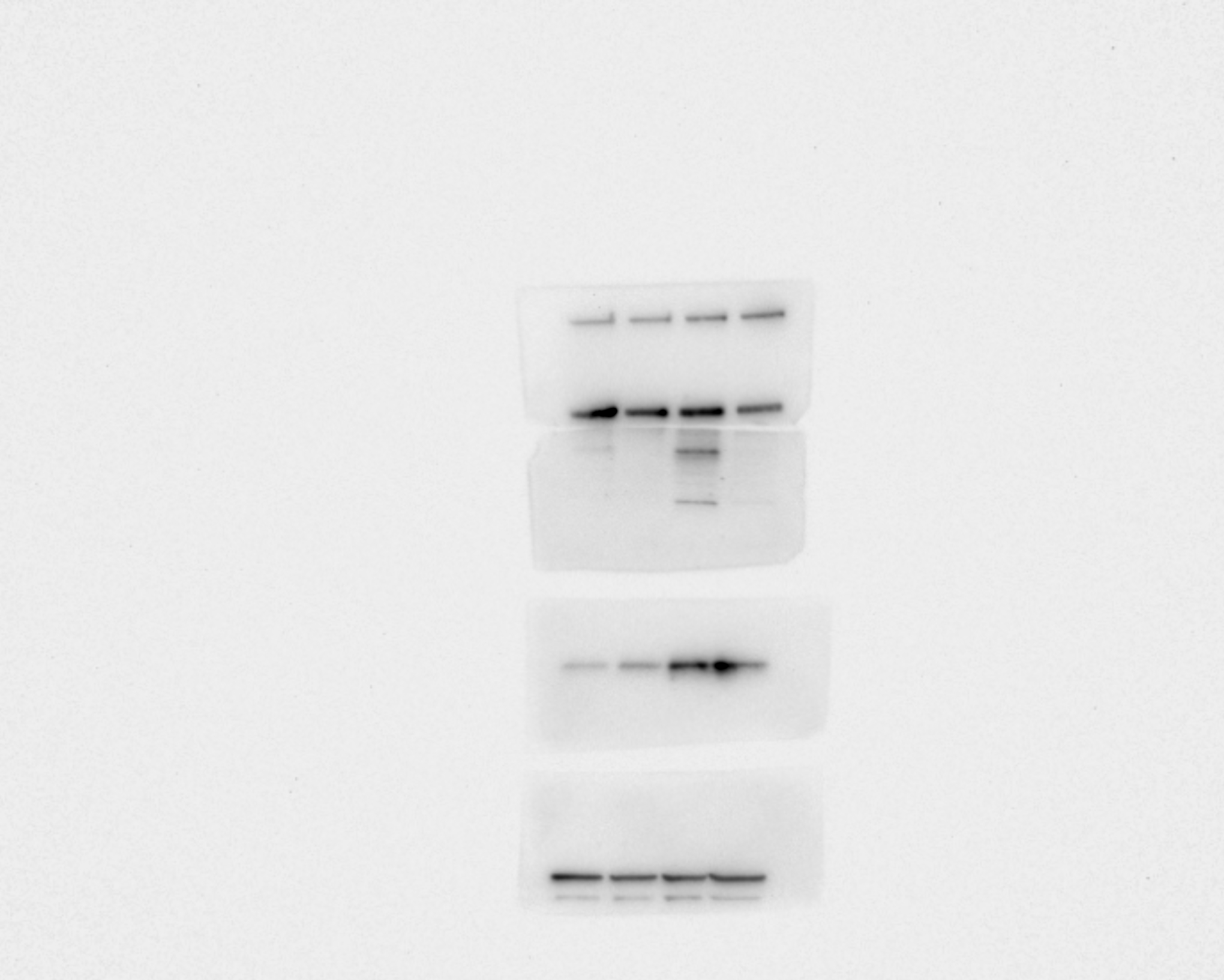

Supplement: Figure 3—source data 3. [file elife-84238-fig3-data3.zip › z Figure 3-Source Data 3/Figure 3-Source Data 3/BAFF,ACTB.jpg]

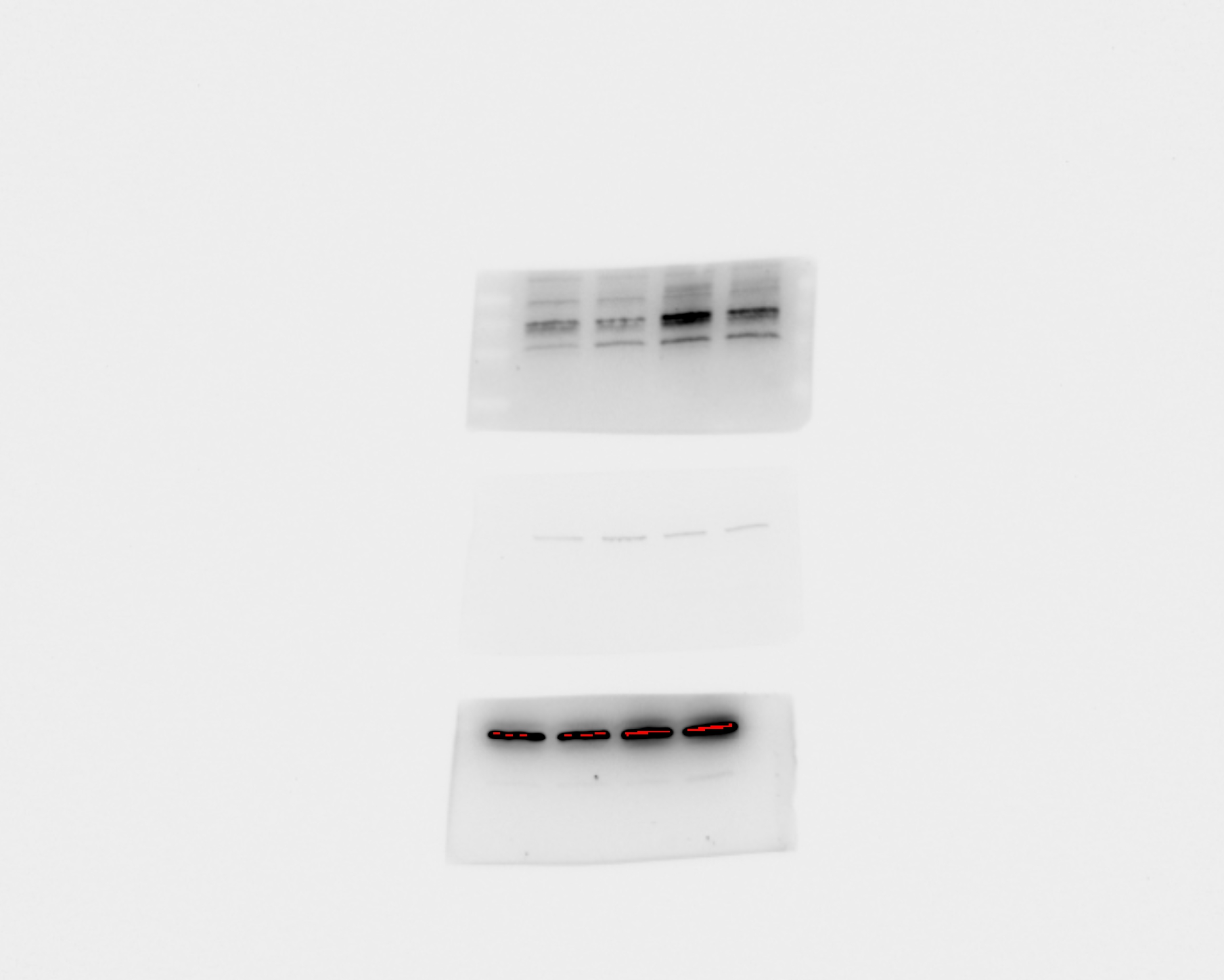

Supplement: Figure 3—source data 3. [file elife-84238-fig3-data3.zip › z Figure 3-Source Data 3/Figure 3-Source Data 3/CATB.jpg]

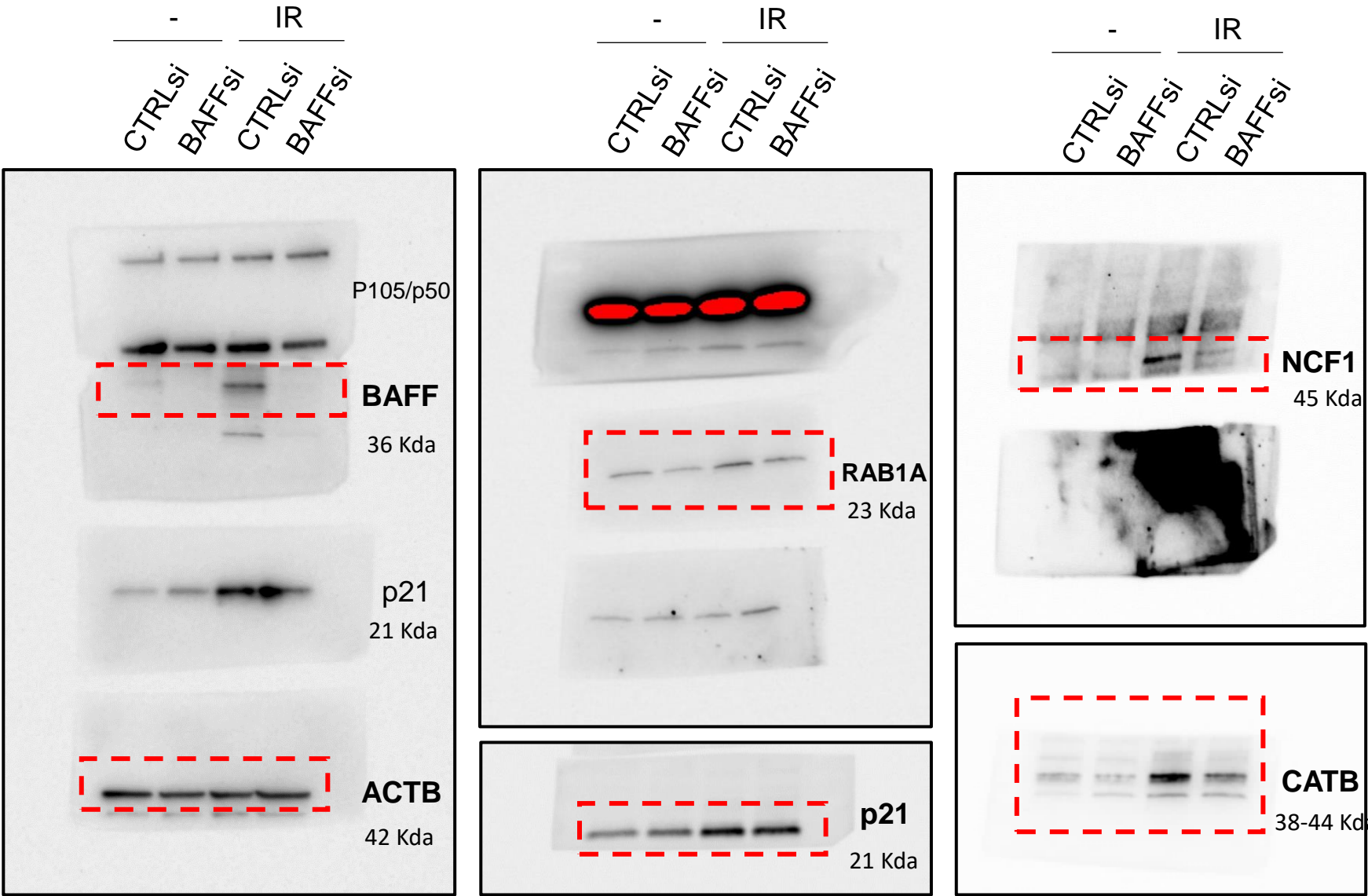

Supplement: Figure 3—source data 3. [file elife-84238-fig3-data3.zip › z Figure 3-Source Data 3/Figure 3-Source Data 3/Figure 3 uncropped blots labeled.pdf]

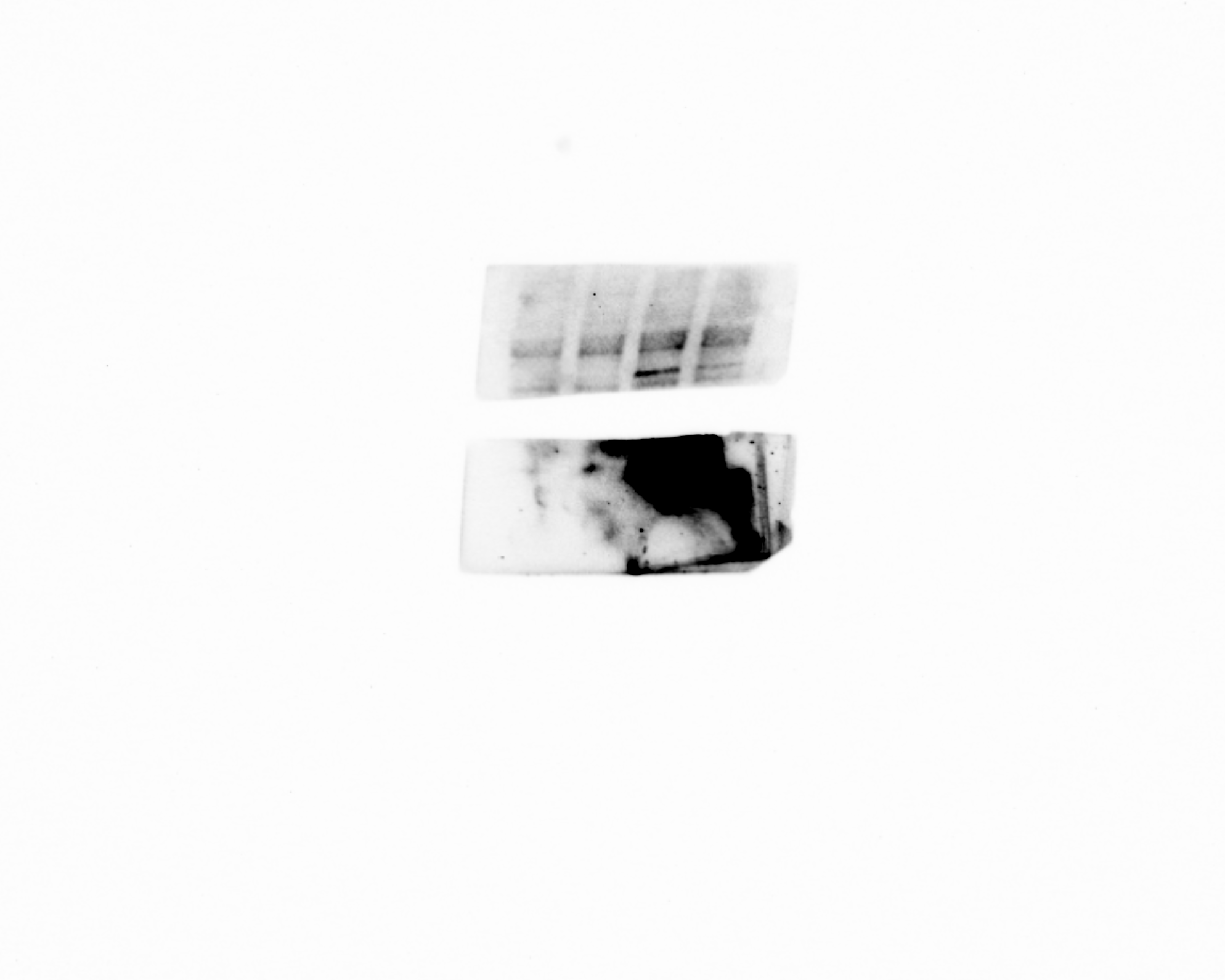

Supplement: Figure 3—source data 3. [file elife-84238-fig3-data3.zip › z Figure 3-Source Data 3/Figure 3-Source Data 3/NCF1.jpg]

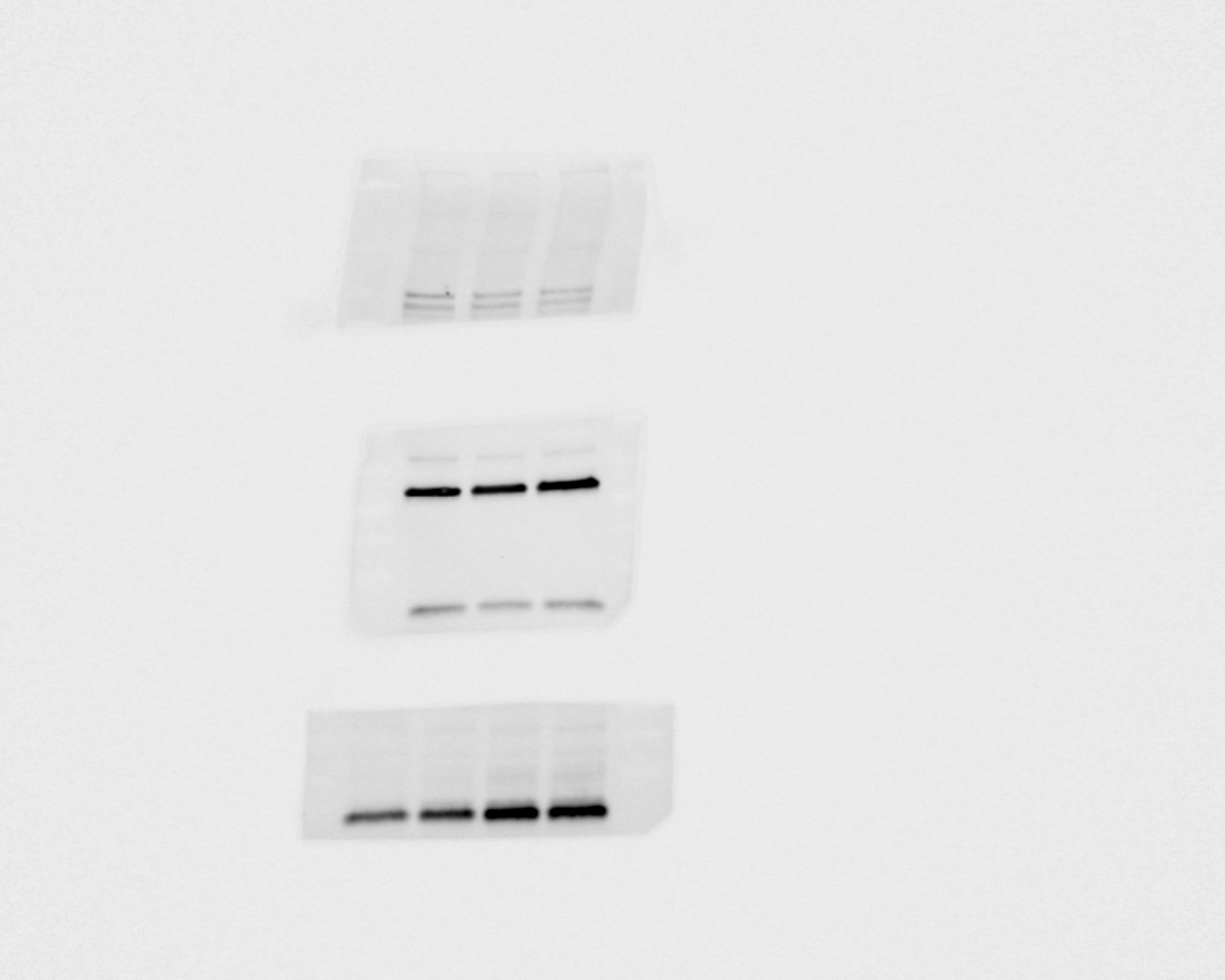

Supplement: Figure 3—source data 3. [file elife-84238-fig3-data3.zip › z Figure 3-Source Data 3/Figure 3-Source Data 3/p21.jpg]

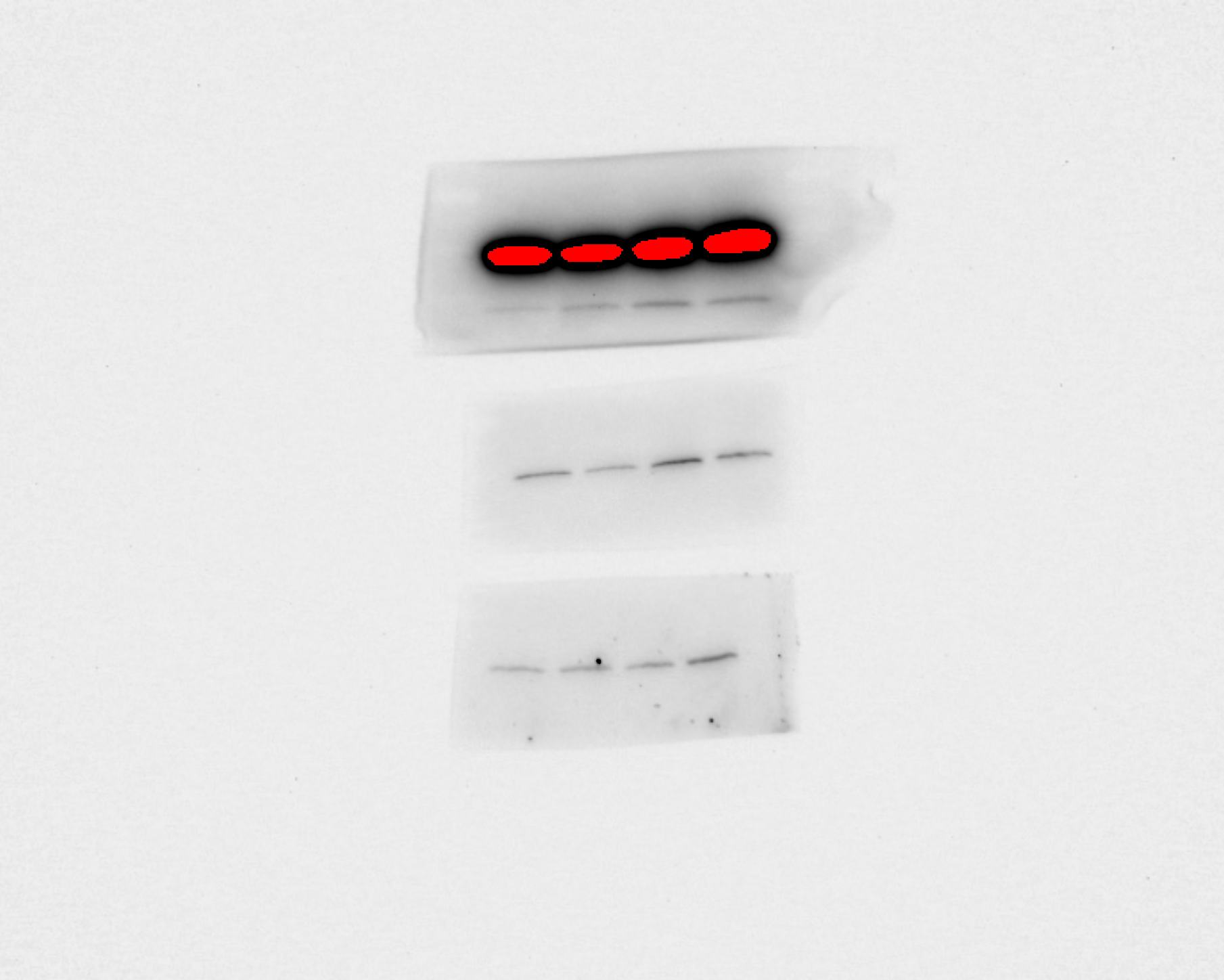

Supplement: Figure 3—source data 3. [file elife-84238-fig3-data3.zip › z Figure 3-Source Data 3/Figure 3-Source Data 3/RAB1A.jpg]

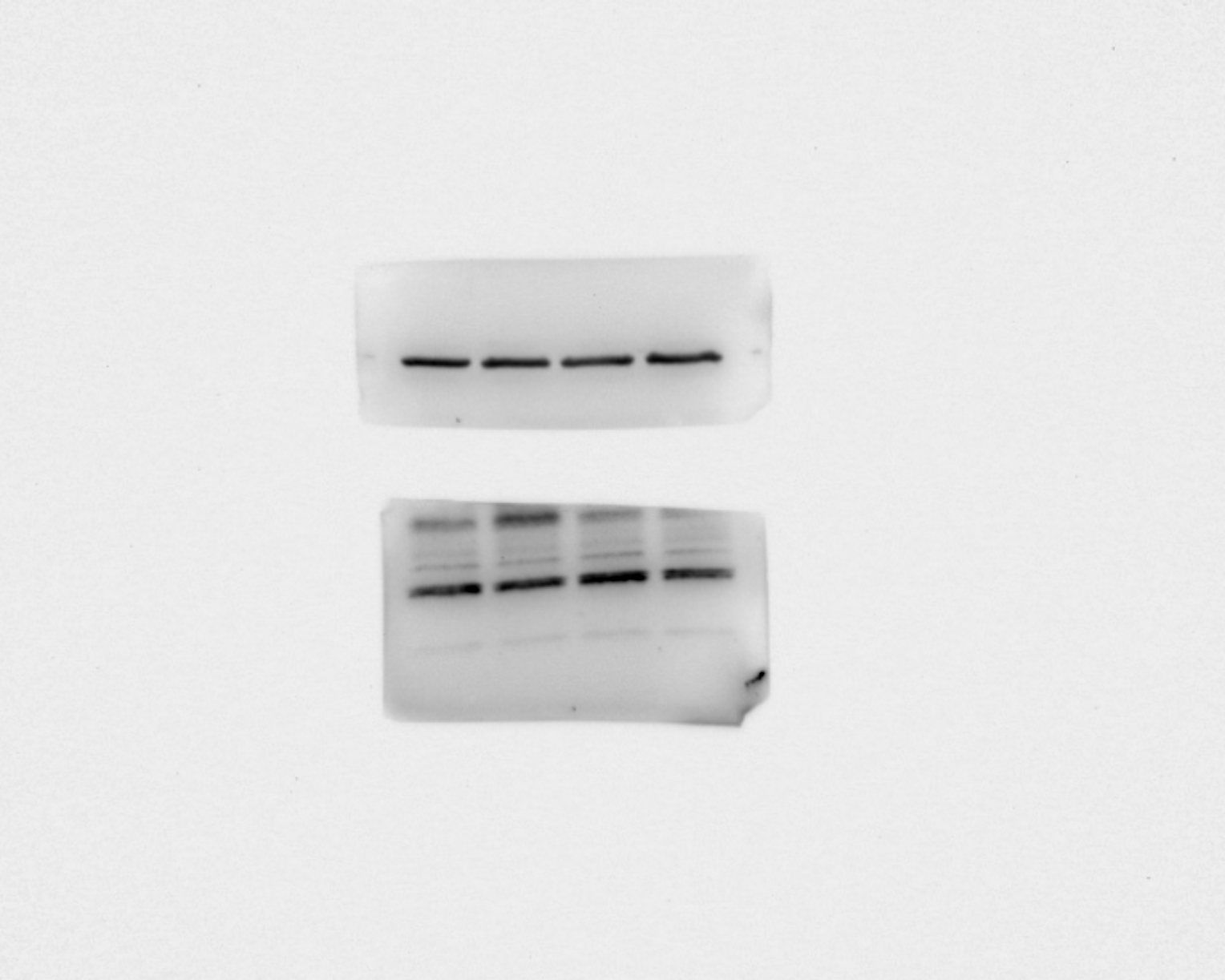

Supplement: Figure 4—source data 1. [file elife-84238-fig4-data1.zip › z Figure 4- Source Data 1/Figure 4- Source Data 1/4D/ACTB.jpg]

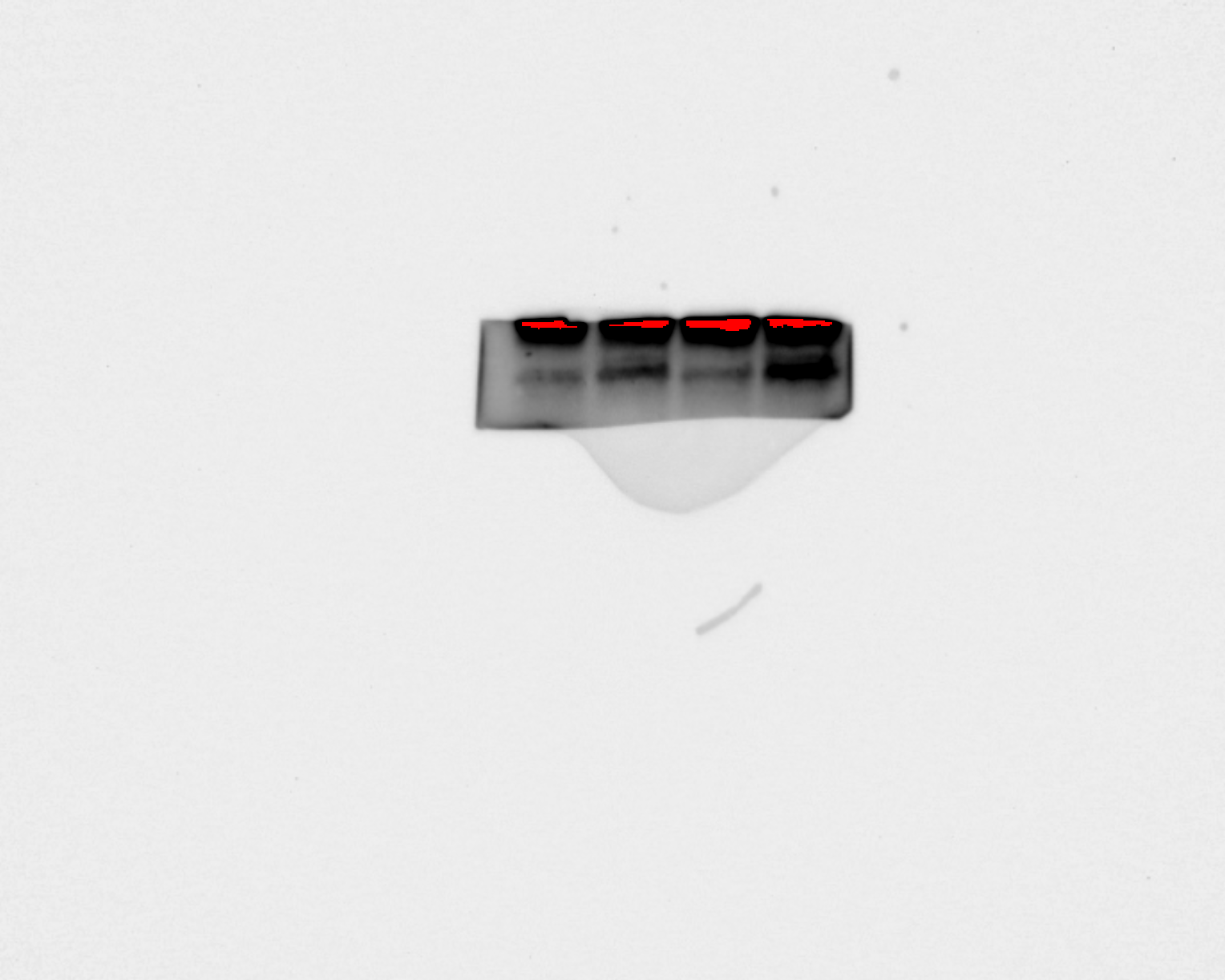

Supplement: Figure 4—source data 1. [file elife-84238-fig4-data1.zip › z Figure 4- Source Data 1/Figure 4- Source Data 1/4D/BAFF.jpg]

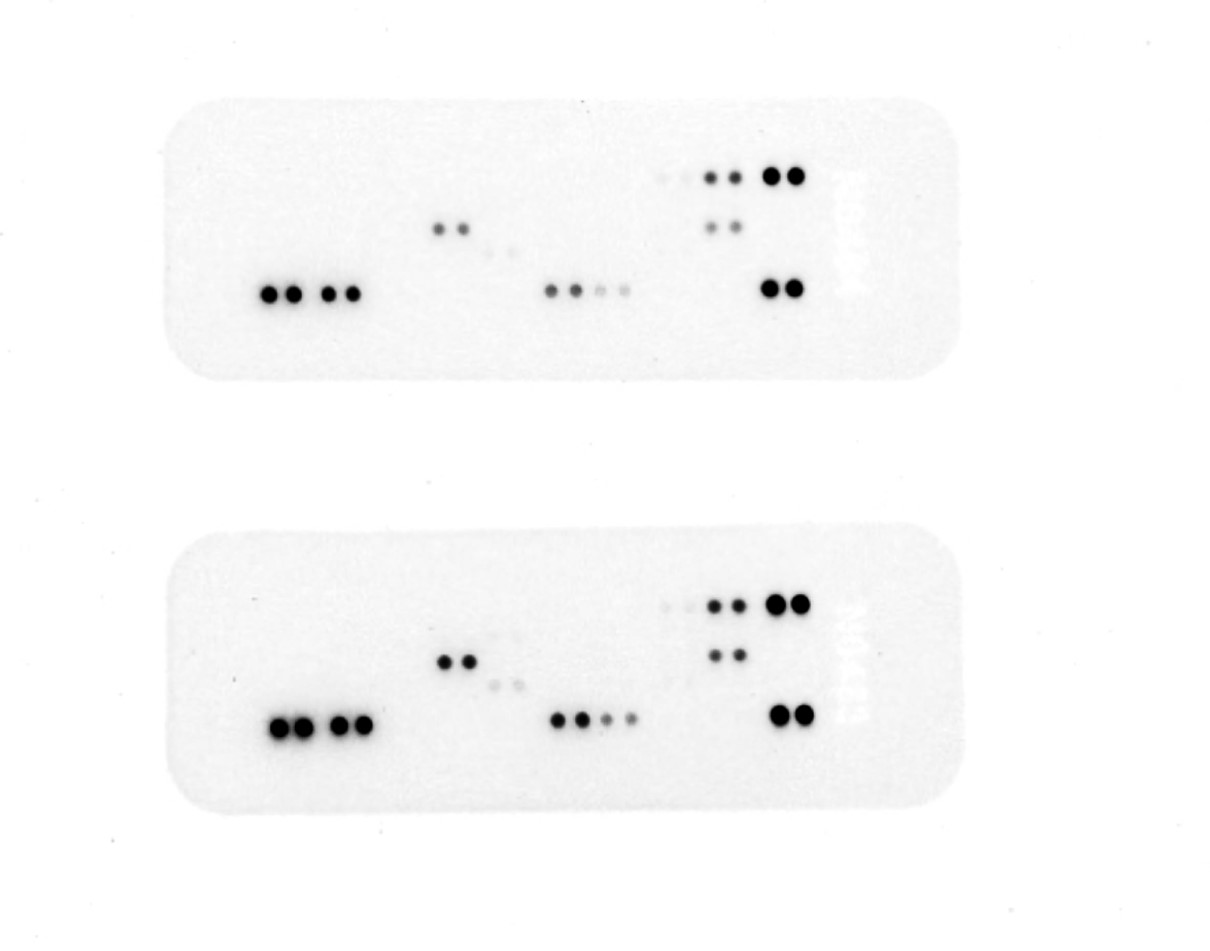

Supplement: Figure 4—source data 1. [file elife-84238-fig4-data1.zip › z Figure 4- Source Data 1/Figure 4- Source Data 1/4F/array unlabeled.jpg]

uncropped blots relative to Fig. 4D

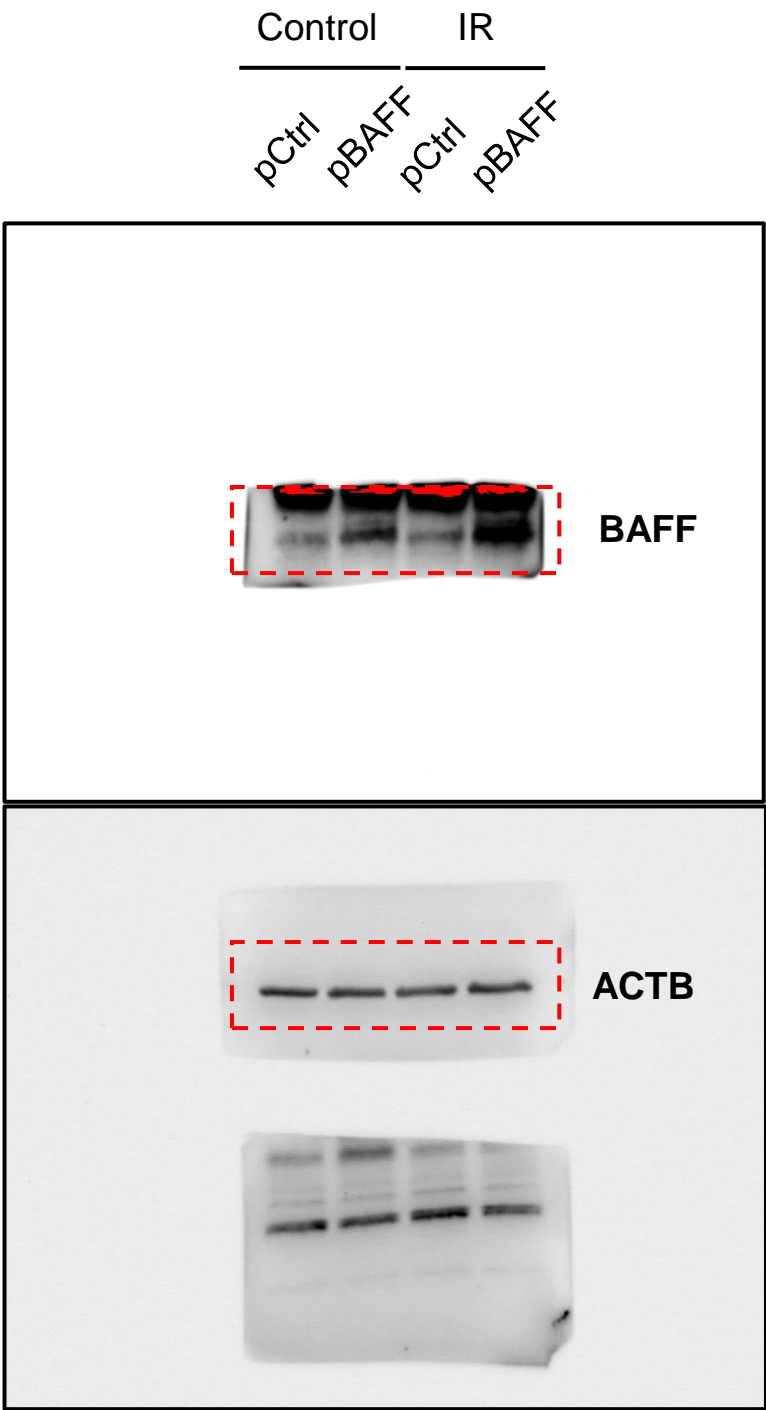

uncropped blots relative to Fig. 4F

Cytokine array in BAFF OE

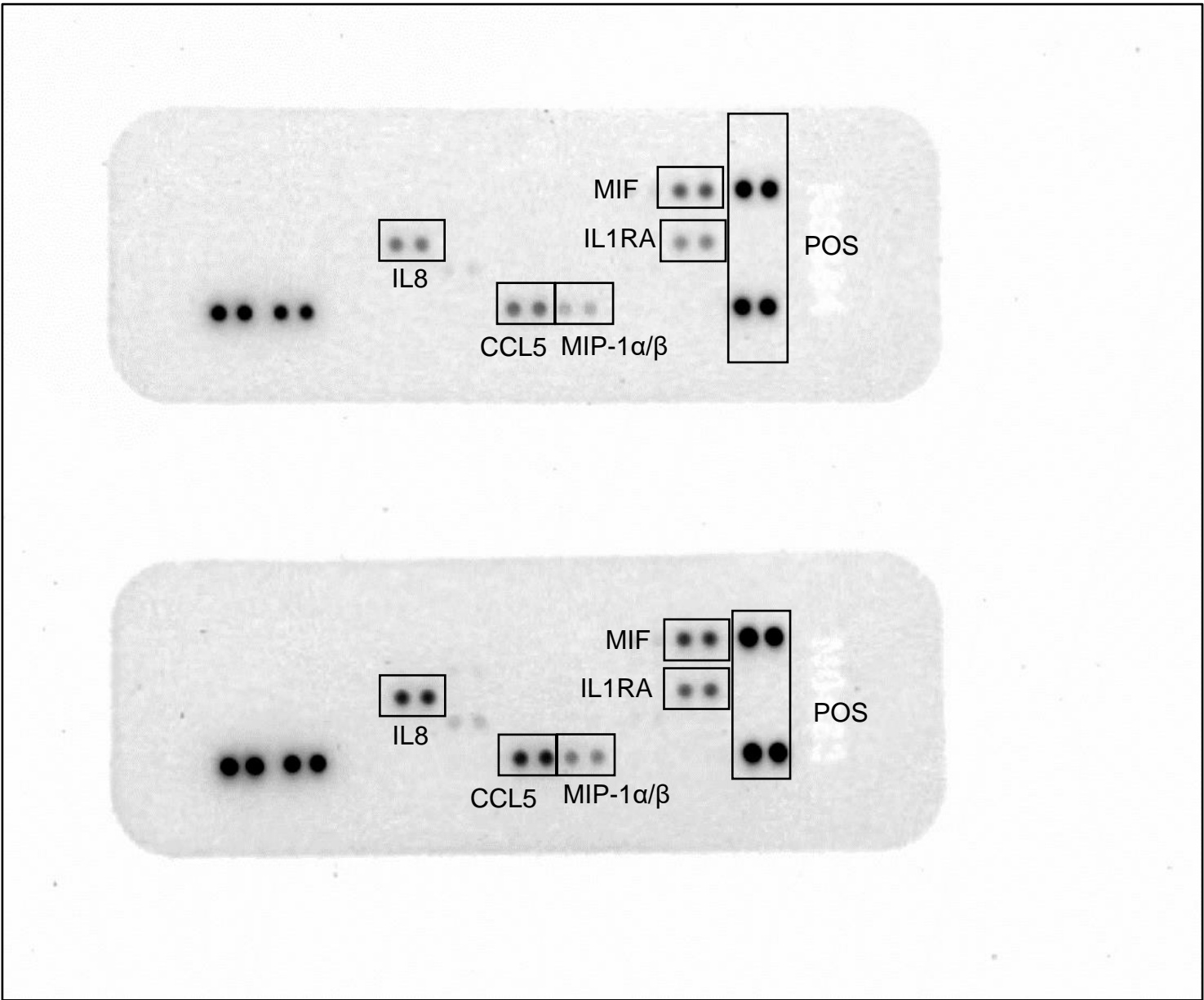

Supplement: Figure 4—source data 1. [file elife-84238-fig4-data1.zip › z Figure 4- Source Data 1/Figure 4- Source Data 1/Figure 4 uncropped blots labeled.pdf]

(High exposure)

Cytokine array in BAFFsi

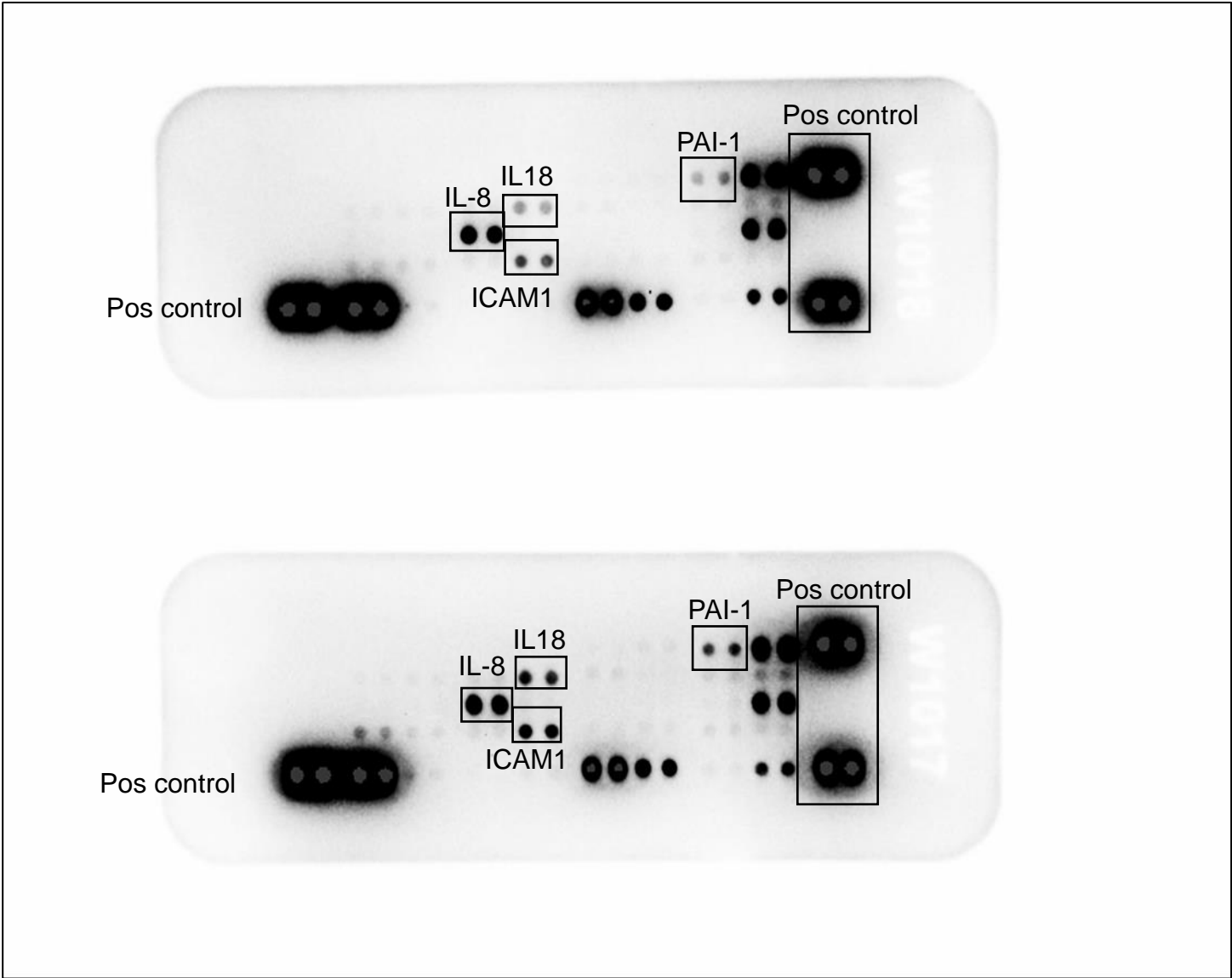

(Low exposure)

Cytokine array in BAFFsi

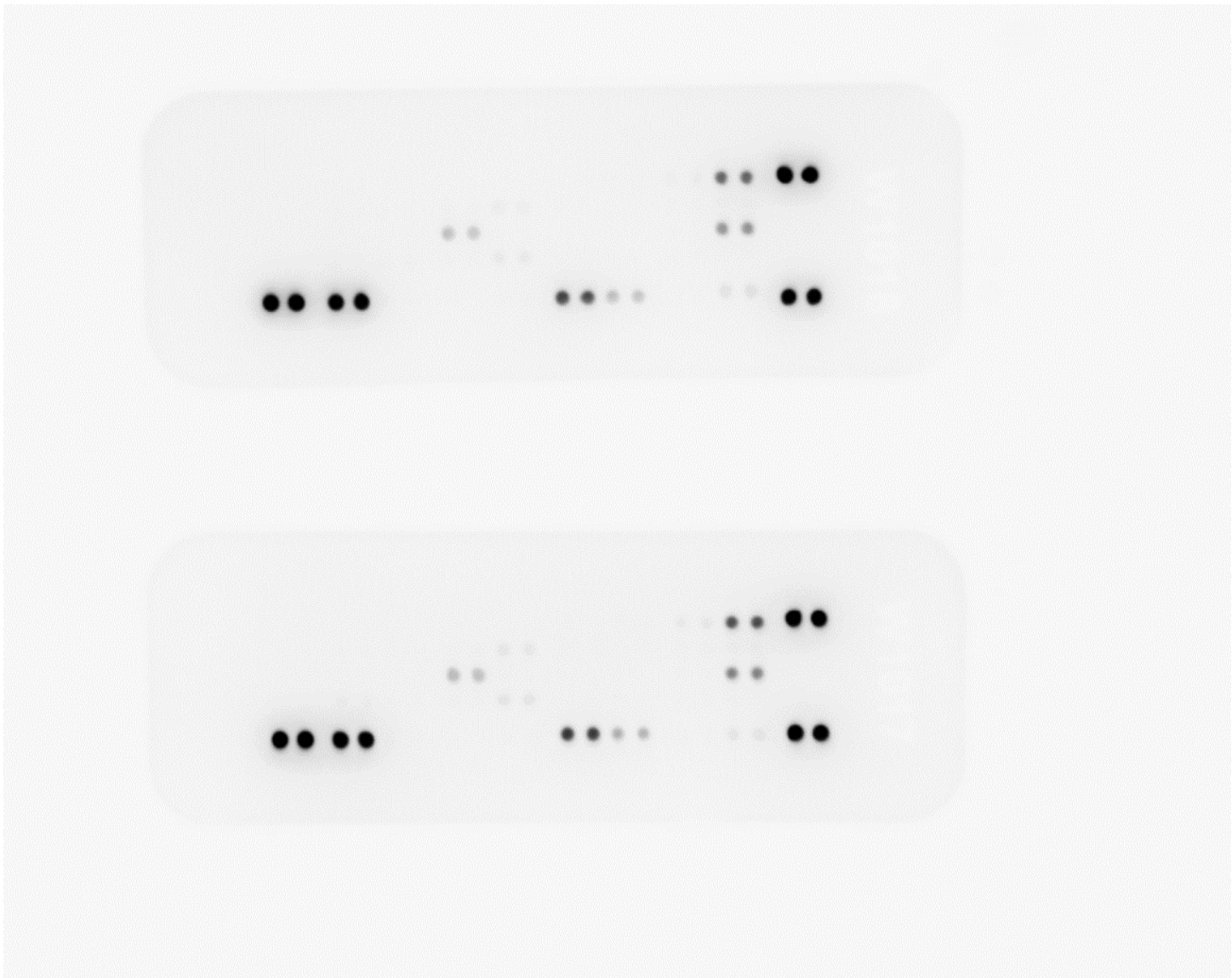

Supplement: Figure 4—figure supplement 1—source data 1. [file elife-84238-fig4-figsupp1-data1.zip › z Figure 4-Figure Supplement 1-Source Data 1/Figure 4-Figure Supplement 1-Source Data 1/Figure S4 uncropped blots.pdf]

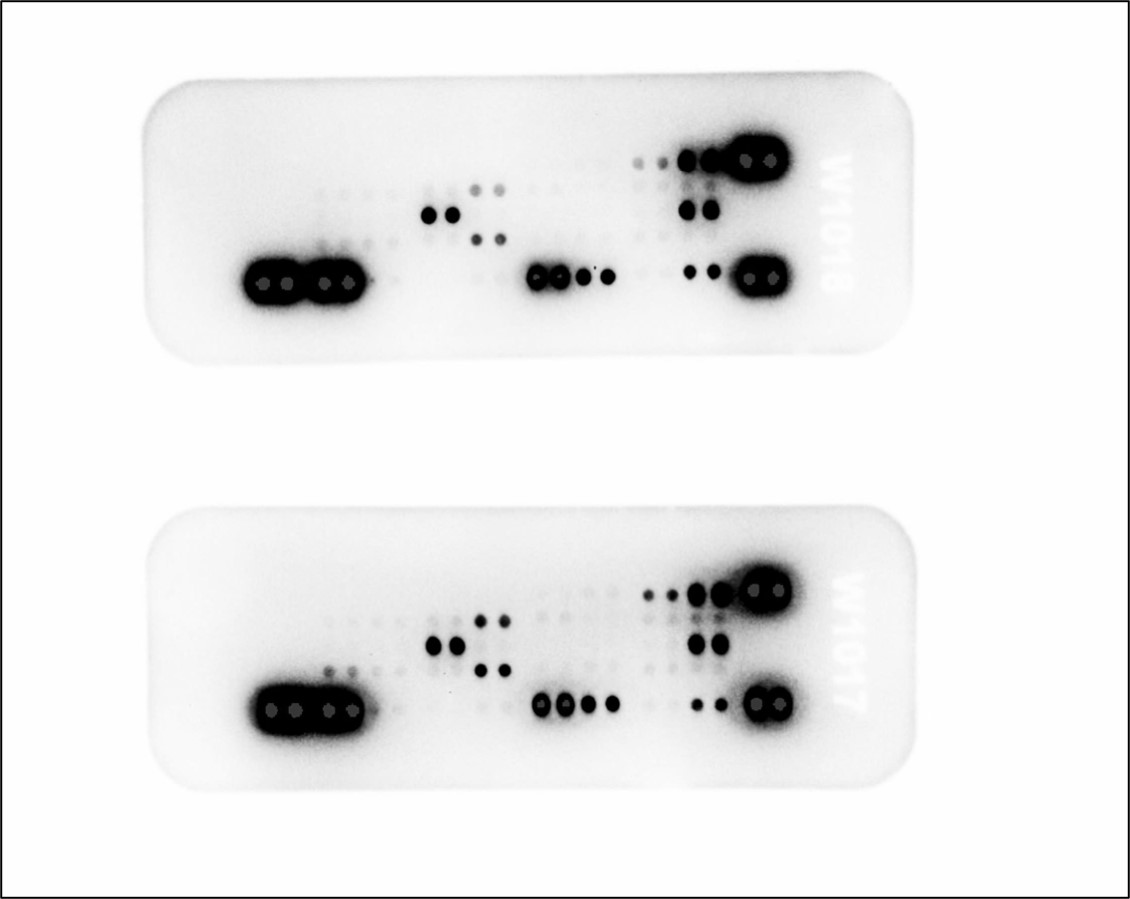

Supplement: Figure 4—figure supplement 1—source data 1. [file elife-84238-fig4-figsupp1-data1.zip › z Figure 4-Figure Supplement 1-Source Data 1/Figure 4-Figure Supplement 1-Source Data 1/original S4C/array high exposure unlabeled.jpg]

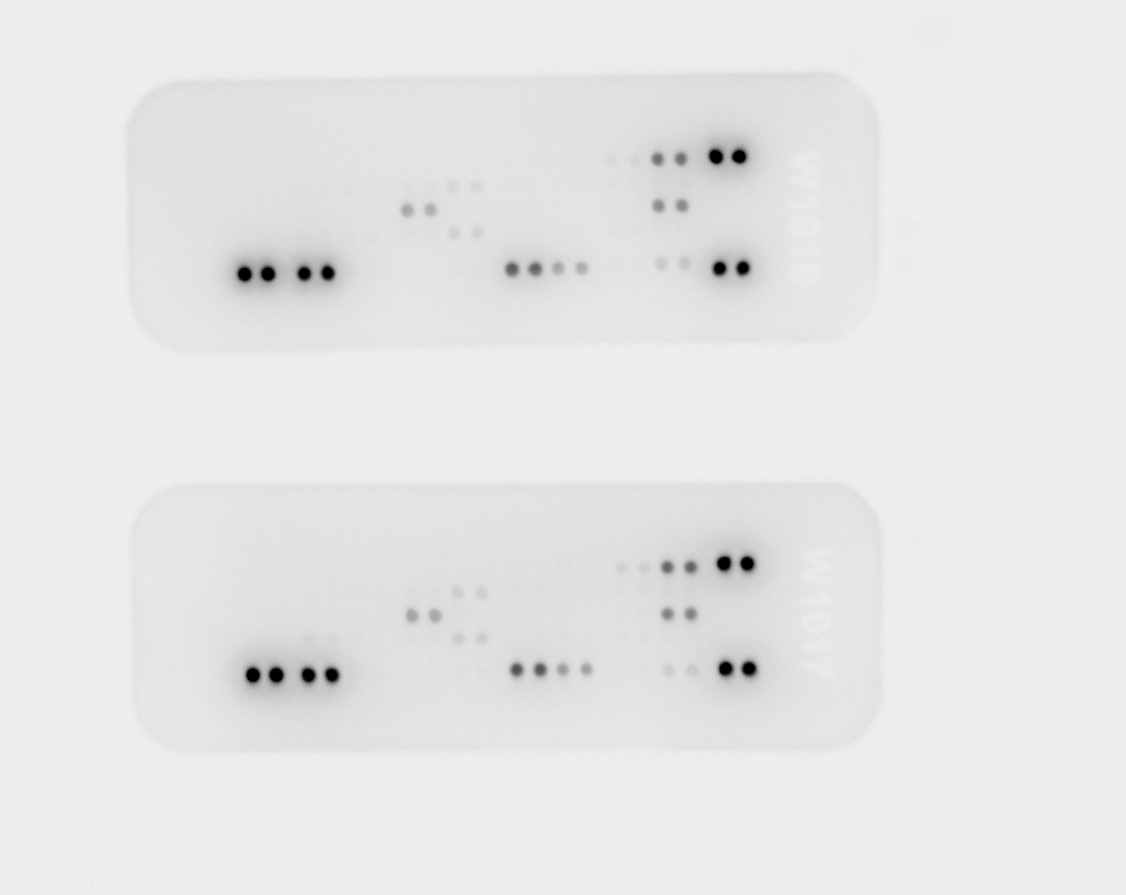

Supplement: Figure 4—figure supplement 1—source data 1. [file elife-84238-fig4-figsupp1-data1.zip › z Figure 4-Figure Supplement 1-Source Data 1/Figure 4-Figure Supplement 1-Source Data 1/original S4C/array low exposure.jpg]

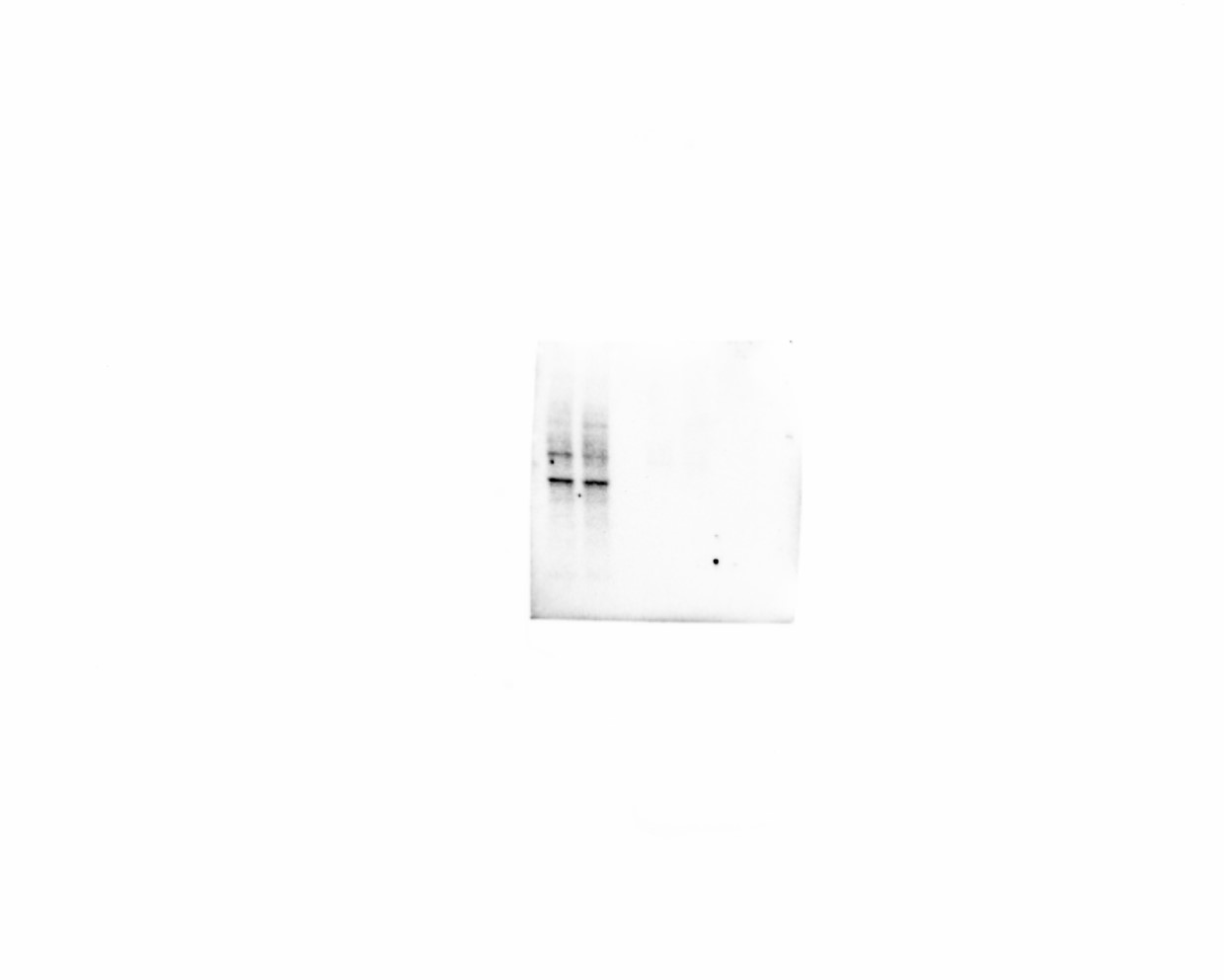

Supplement: Figure 5—source data 1. [file elife-84238-fig5-data1.zip › z Figure 5-Source Data 1/Figure 5-Source Data 1/Fig 5A/ACTB.jpg]

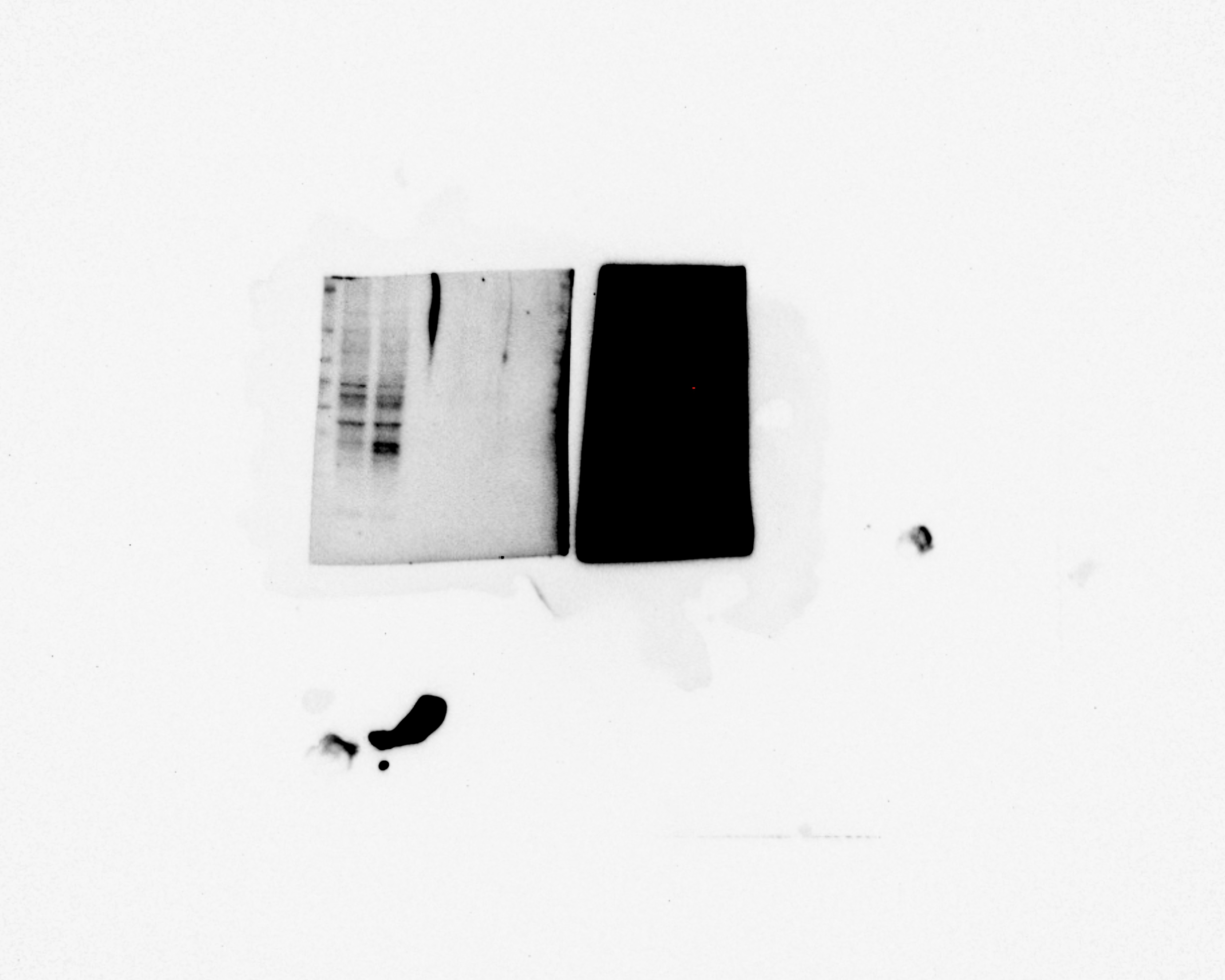

Supplement: Figure 5—source data 1. [file elife-84238-fig5-data1.zip › z Figure 5-Source Data 1/Figure 5-Source Data 1/Fig 5A/BAFF.jpg]

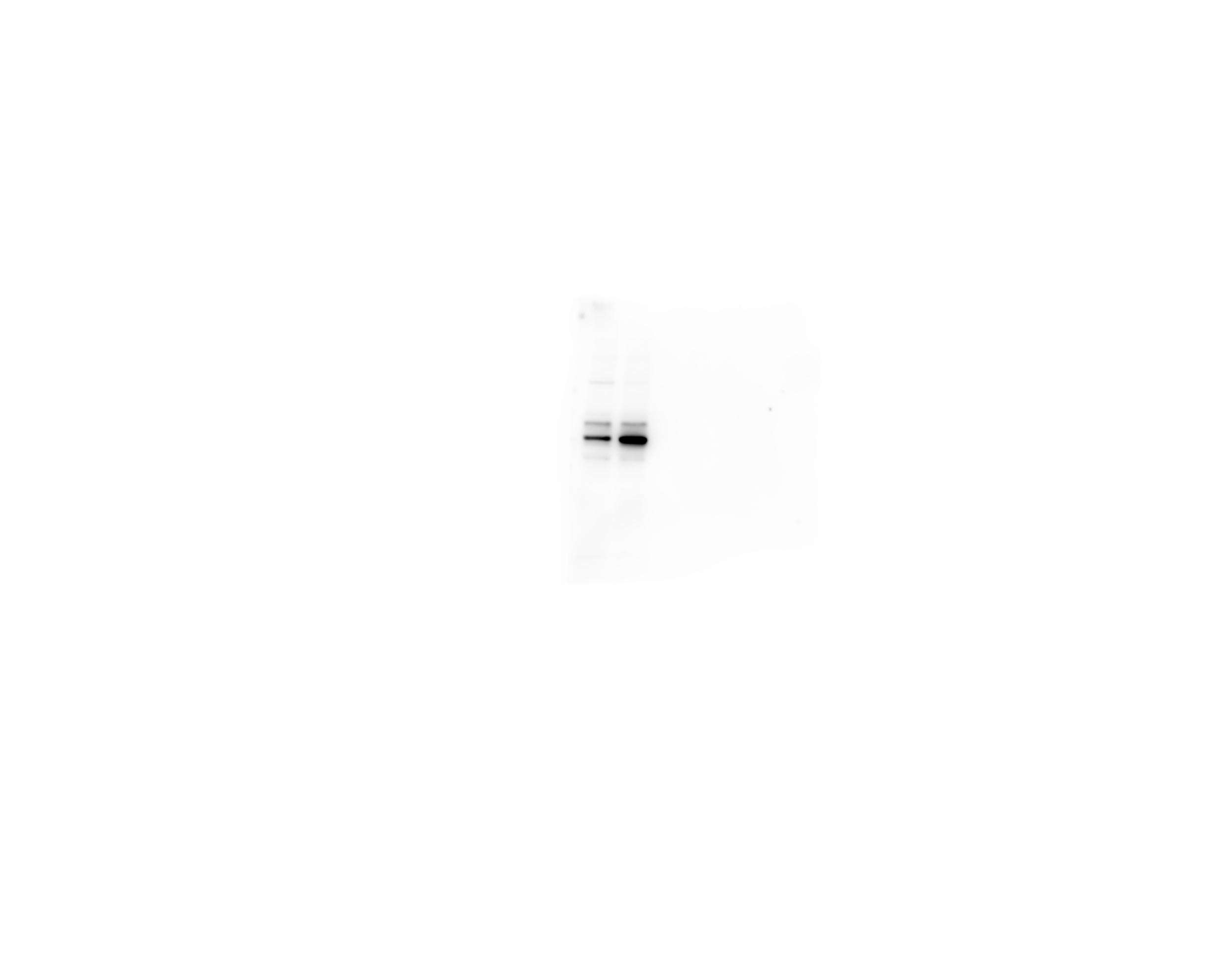

Supplement: Figure 5—source data 1. [file elife-84238-fig5-data1.zip › z Figure 5-Source Data 1/Figure 5-Source Data 1/Fig 5A/BAFFR.jpg]

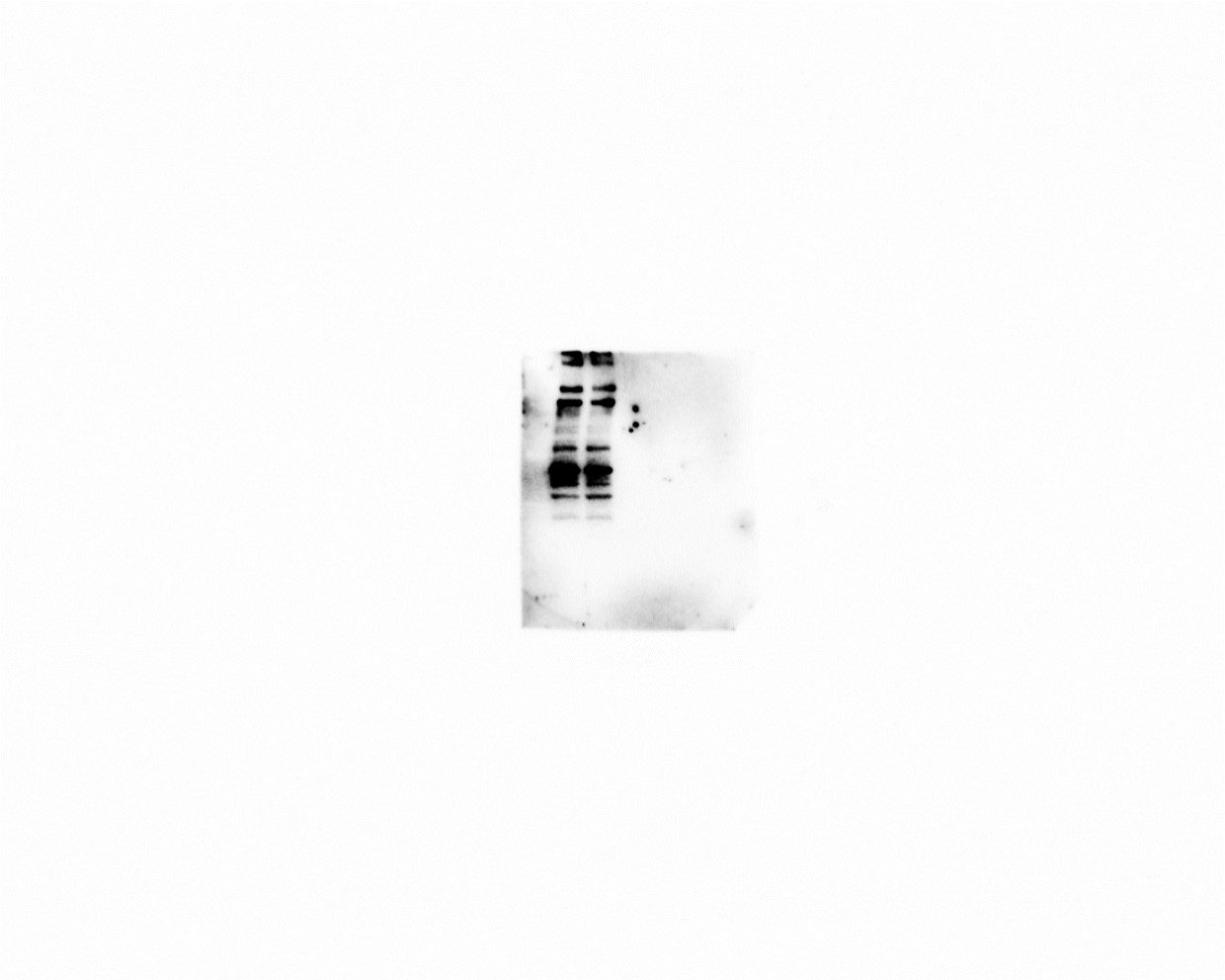

Supplement: Figure 5—source data 1. [file elife-84238-fig5-data1.zip › z Figure 5-Source Data 1/Figure 5-Source Data 1/Fig 5A/BCMA.jpg]

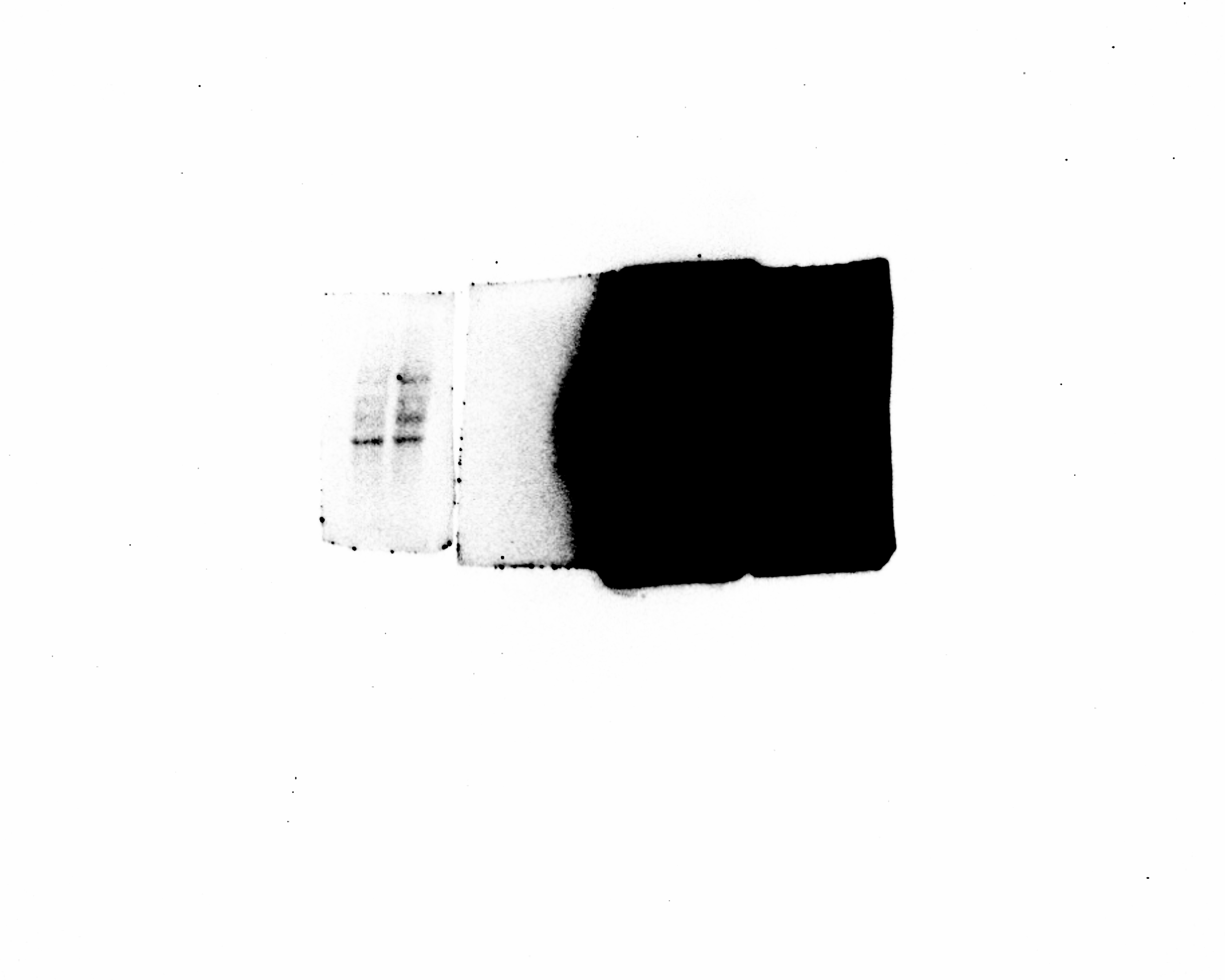

Supplement: Figure 5—source data 1. [file elife-84238-fig5-data1.zip › z Figure 5-Source Data 1/Figure 5-Source Data 1/Fig 5A/TACI high exposure.jpg]

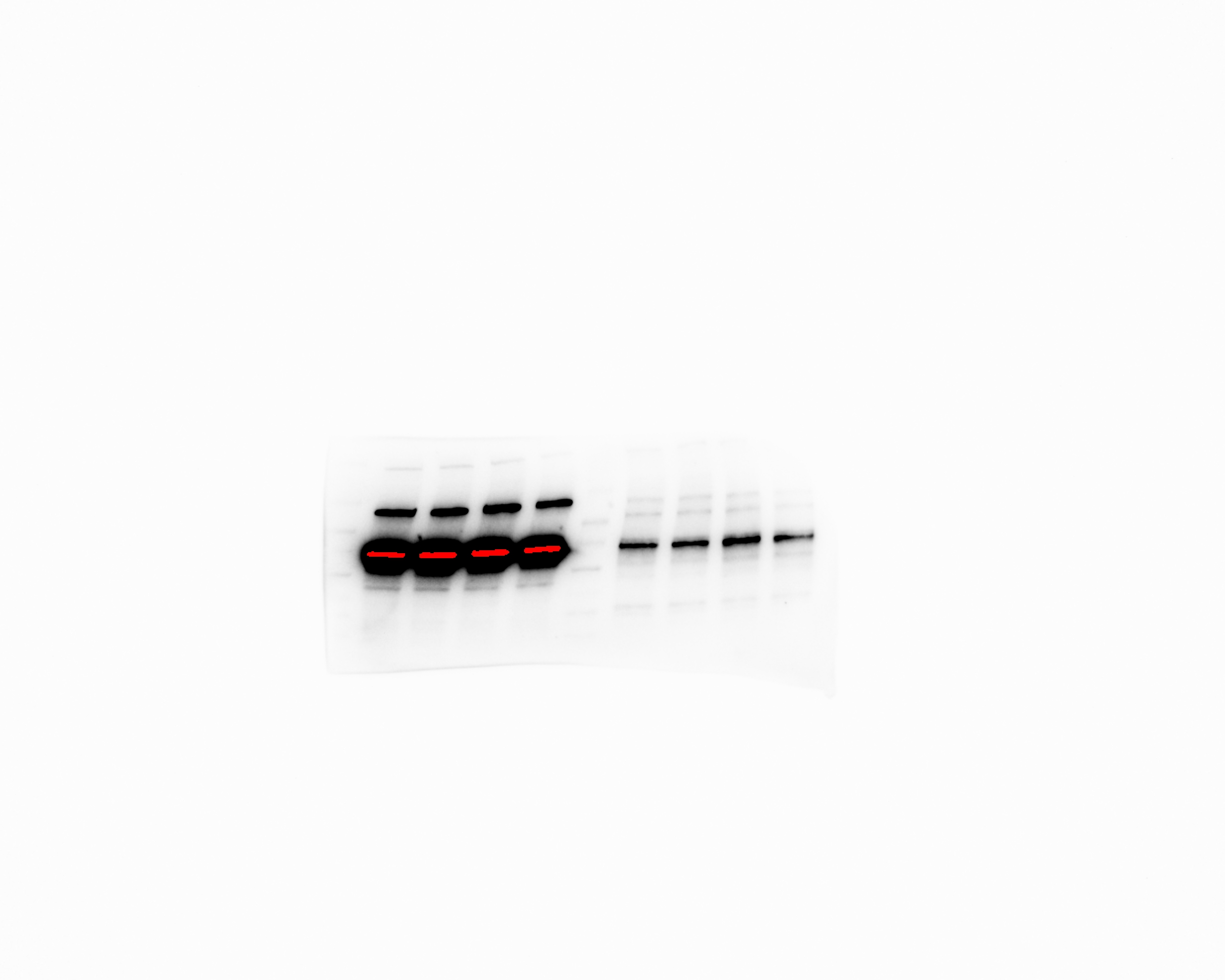

Supplement: Figure 5—source data 1. [file elife-84238-fig5-data1.zip › z Figure 5-Source Data 1/Figure 5-Source Data 1/Fig 5F/p65 high exposure.jpg]

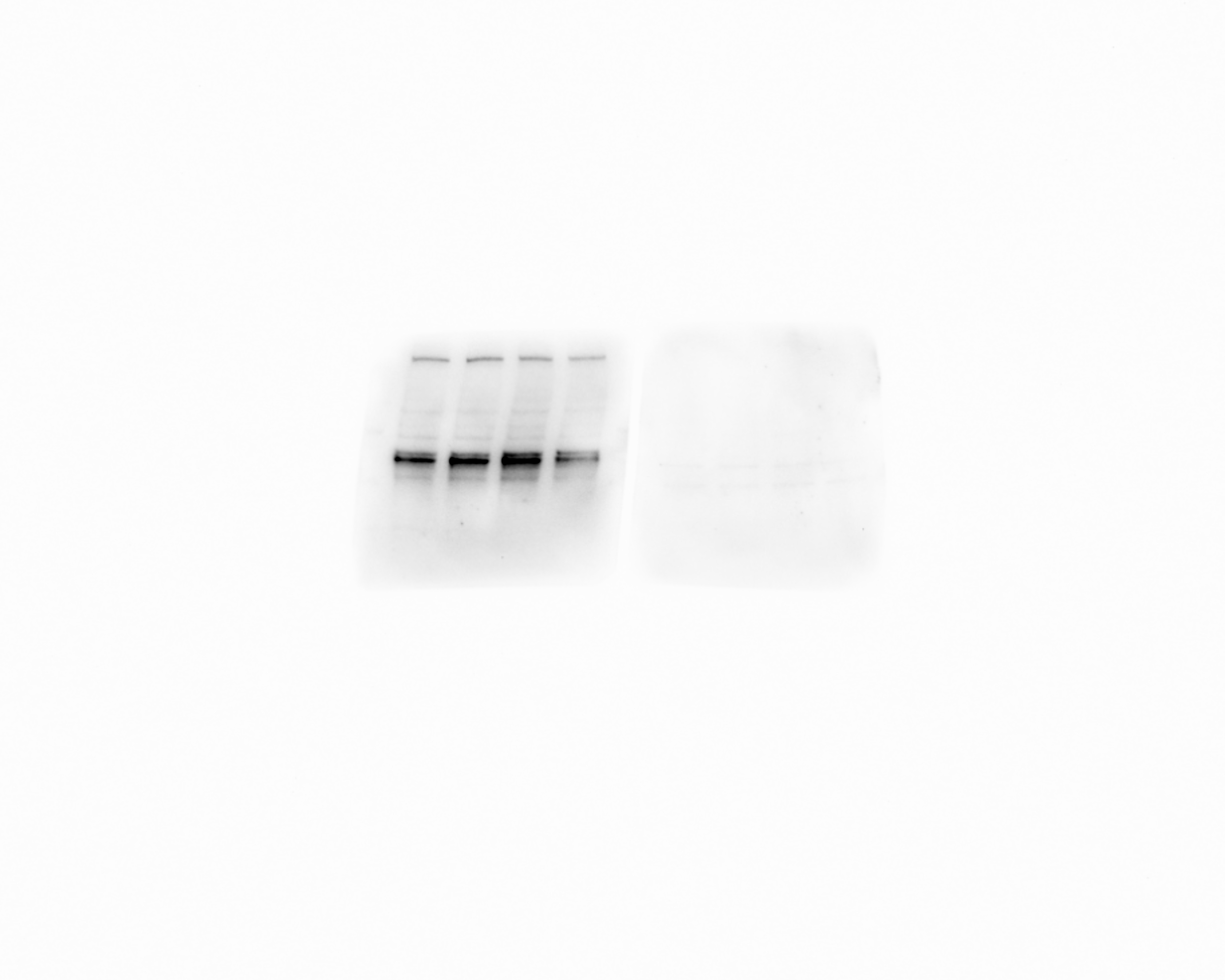

Supplement: Figure 5—source data 1. [file elife-84238-fig5-data1.zip › z Figure 5-Source Data 1/Figure 5-Source Data 1/Fig 5F/p65,Tubulin nuclei.jpg]

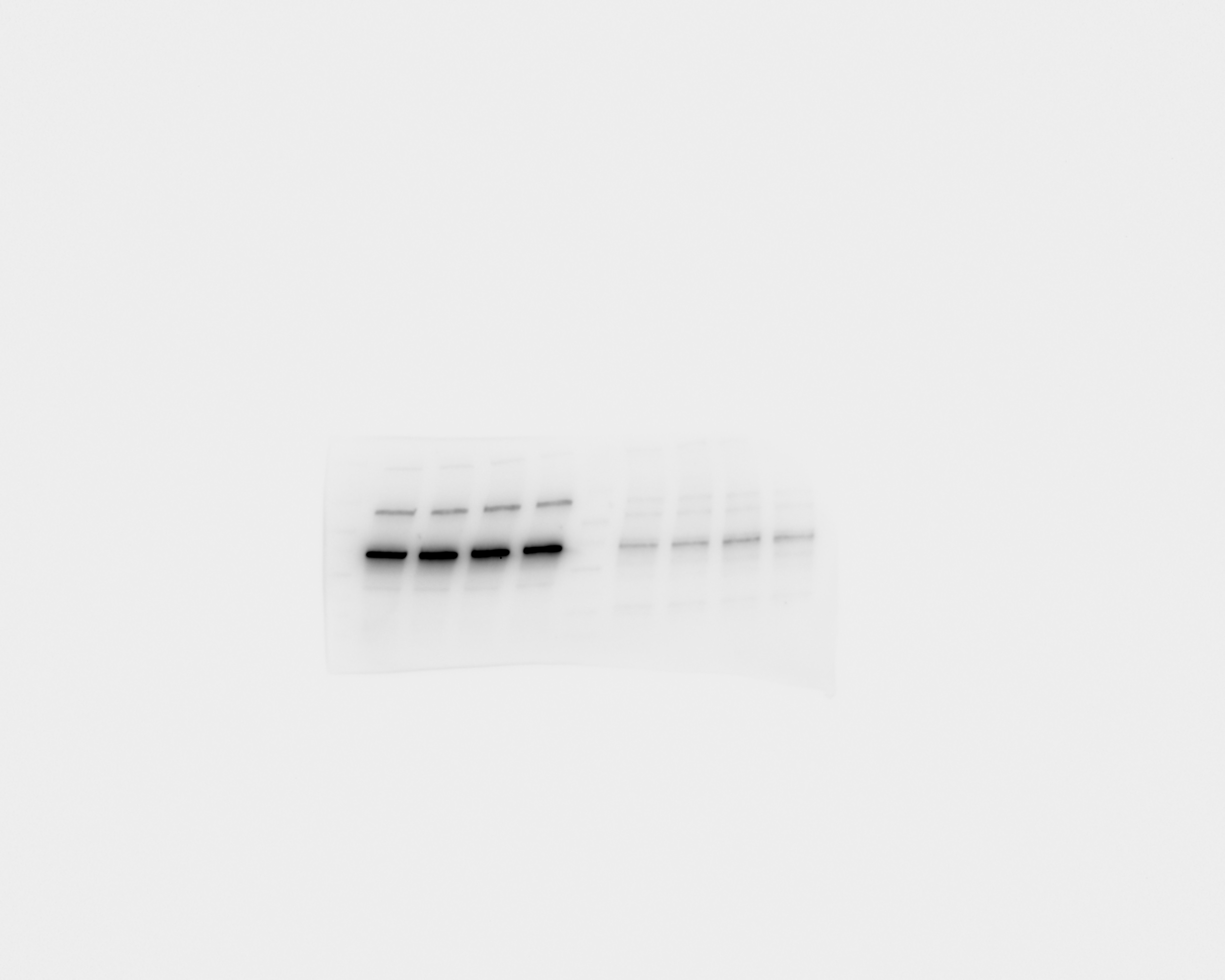

Supplement: Figure 5—source data 1. [file elife-84238-fig5-data1.zip › z Figure 5-Source Data 1/Figure 5-Source Data 1/Fig 5F/p65.jpg]

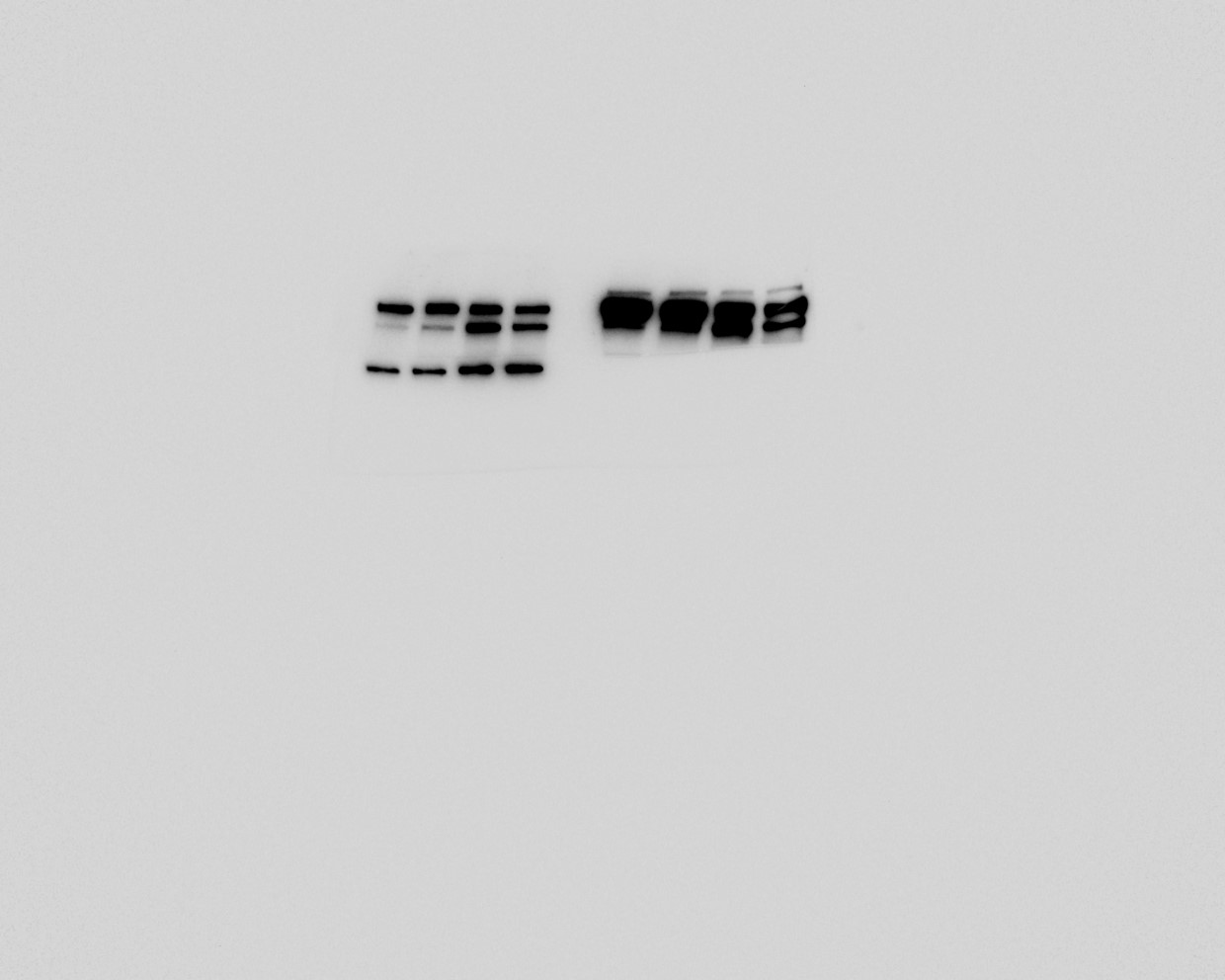

Supplement: Figure 5—source data 1. [file elife-84238-fig5-data1.zip › z Figure 5-Source Data 1/Figure 5-Source Data 1/Fig 5F/Parp, Tubulin high exposure.jpg]

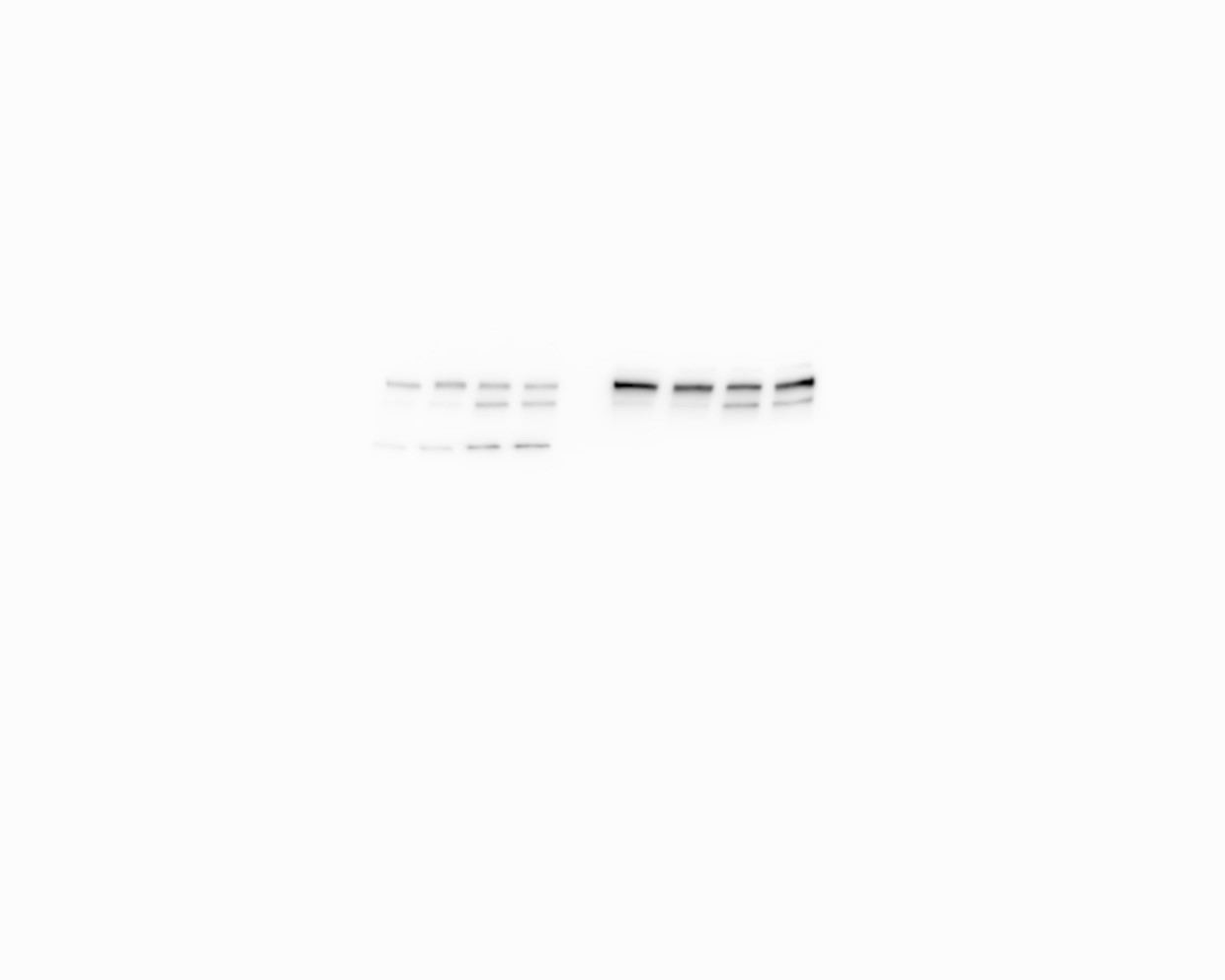

Supplement: Figure 5—source data 1. [file elife-84238-fig5-data1.zip › z Figure 5-Source Data 1/Figure 5-Source Data 1/Fig 5F/Parp,Tubulin lower exposure.jpg]

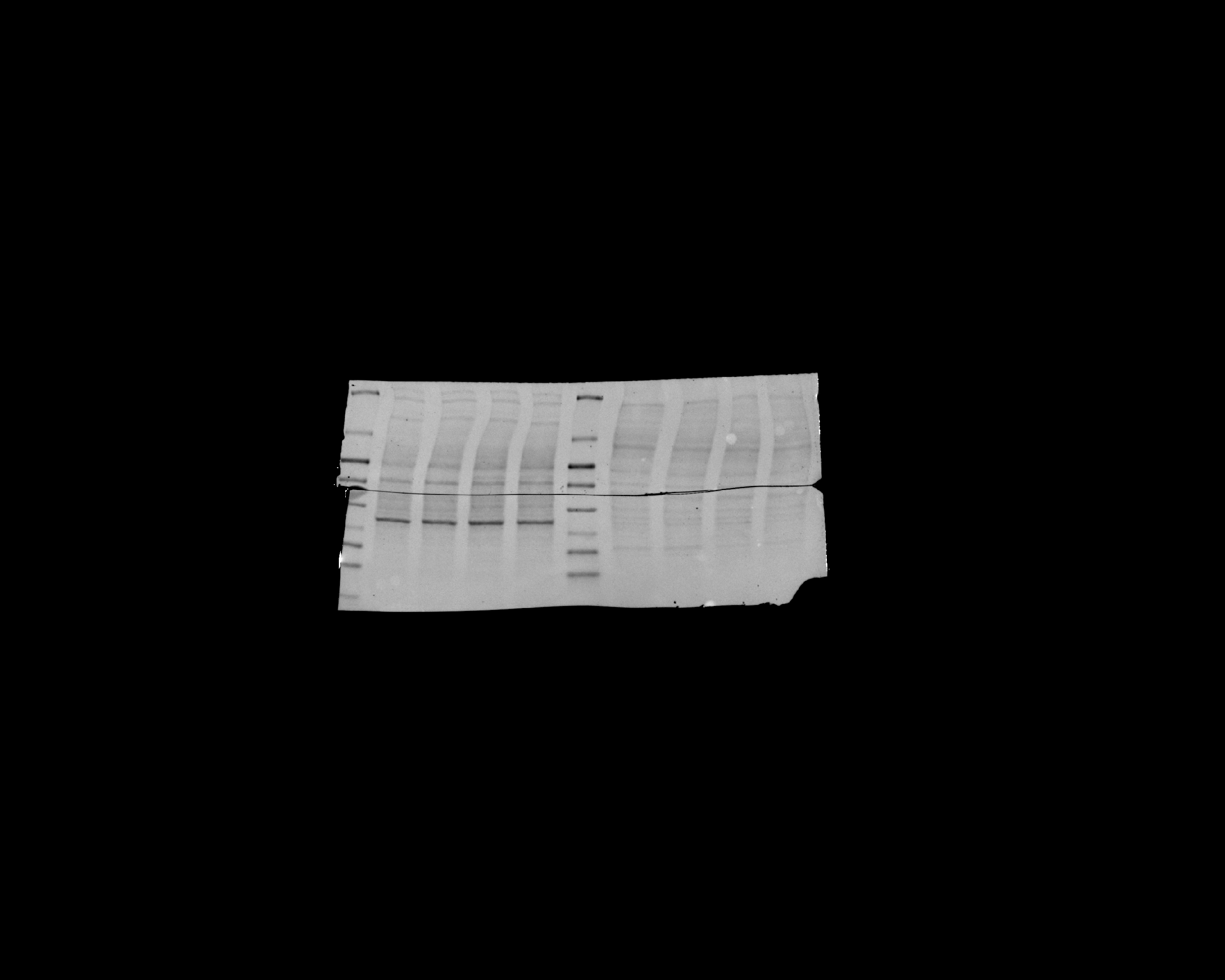

Supplement: Figure 5—source data 1. [file elife-84238-fig5-data1.zip › z Figure 5-Source Data 1/Figure 5-Source Data 1/Fig 5F/ponceau S.jpg]

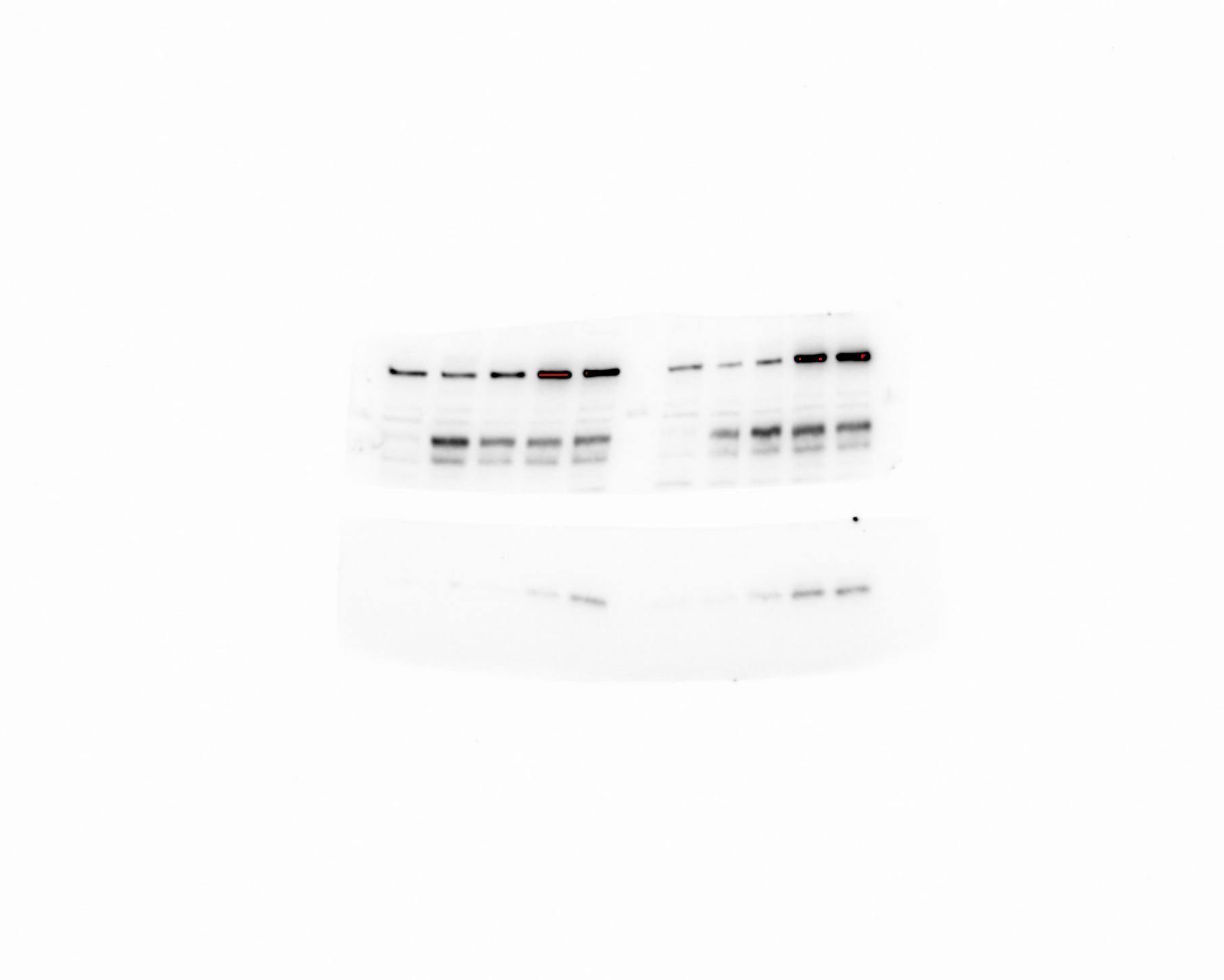

Supplement: Figure 5—source data 1. [file elife-84238-fig5-data1.zip › z Figure 5-Source Data 1/Figure 5-Source Data 1/Fig 5G/Ac-p65.jpg]

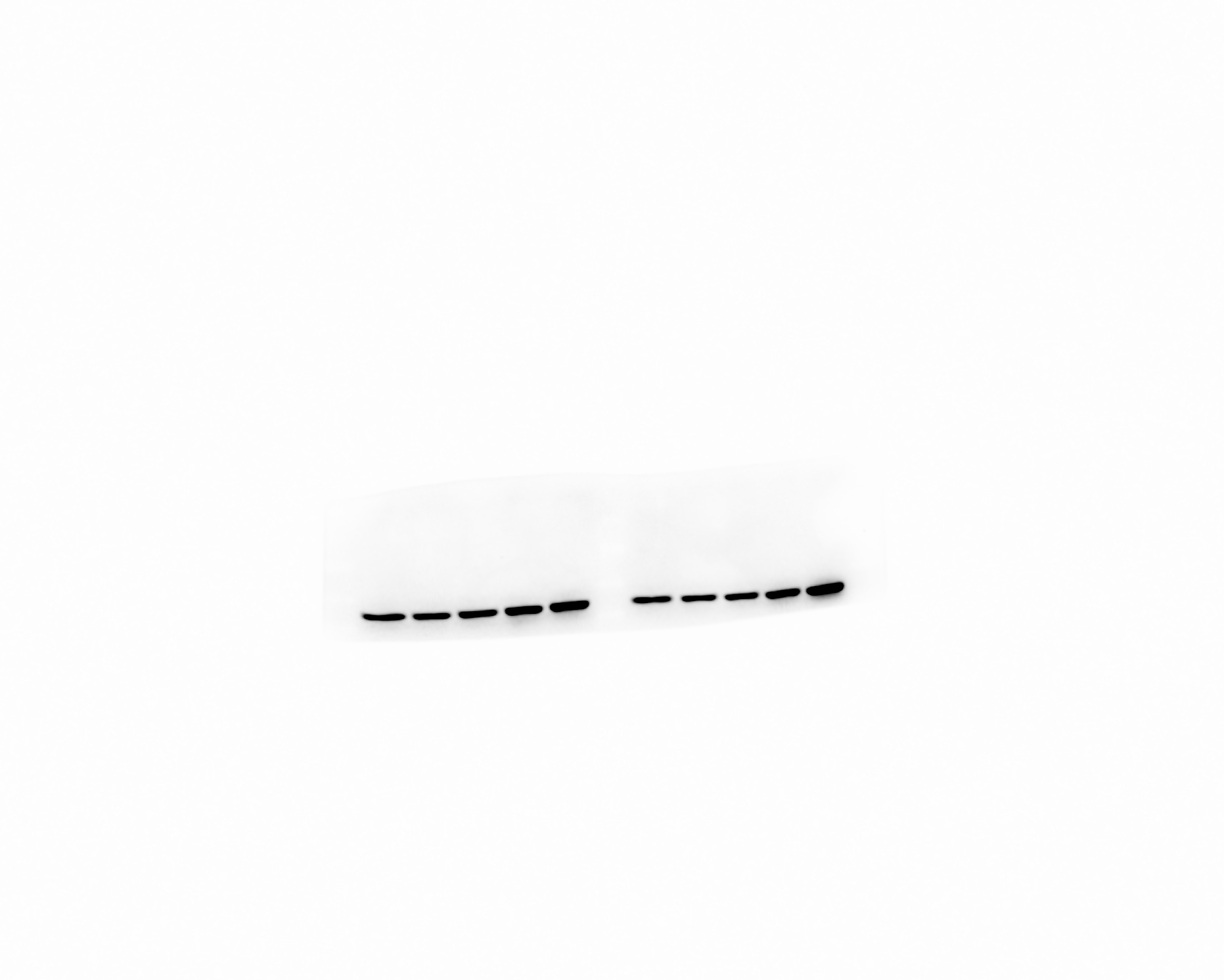

Supplement: Figure 5—source data 1. [file elife-84238-fig5-data1.zip › z Figure 5-Source Data 1/Figure 5-Source Data 1/Fig 5G/ACTB.jpg]

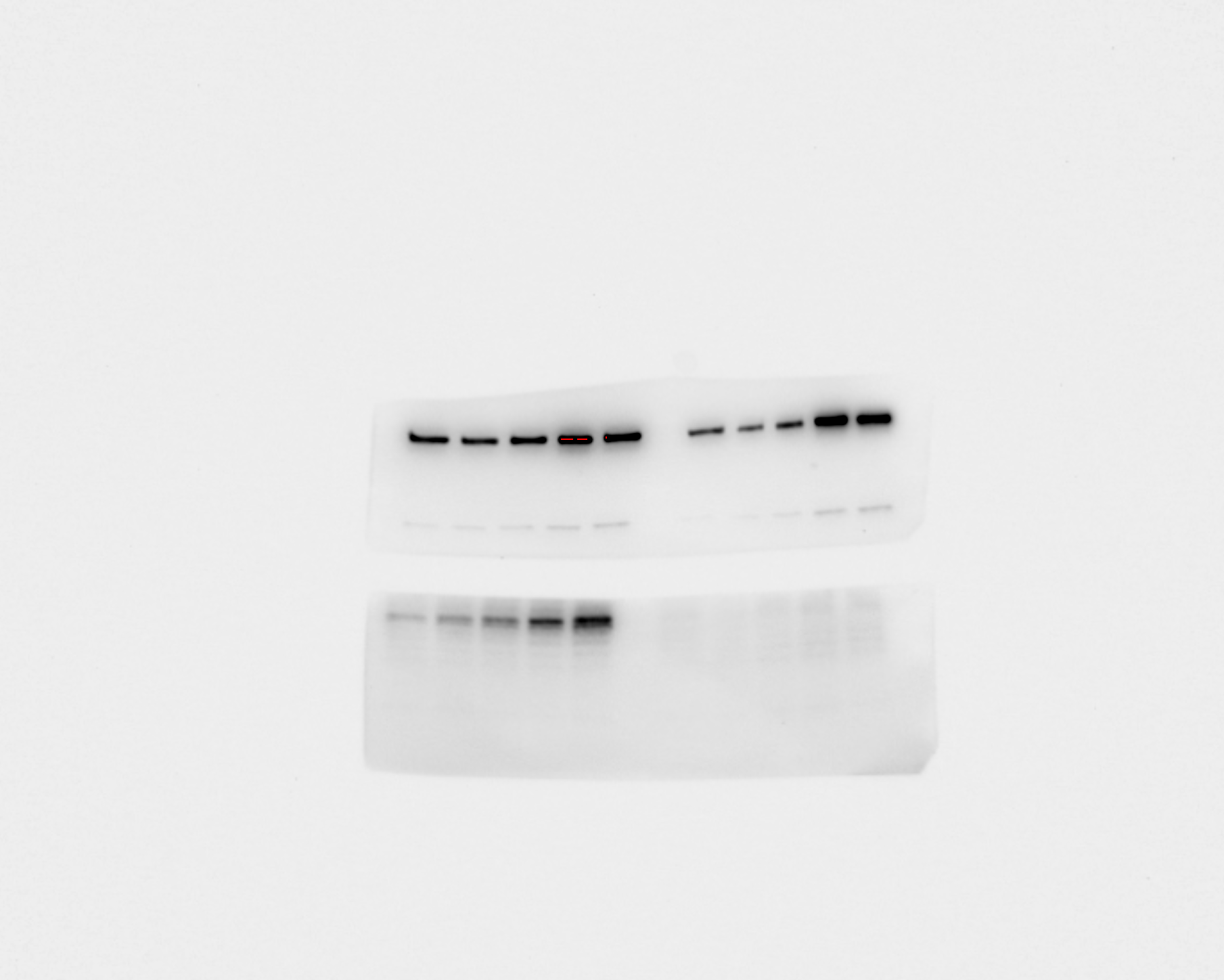

Supplement: Figure 5—source data 1. [file elife-84238-fig5-data1.zip › z Figure 5-Source Data 1/Figure 5-Source Data 1/Fig 5G/BAFF.jpg]

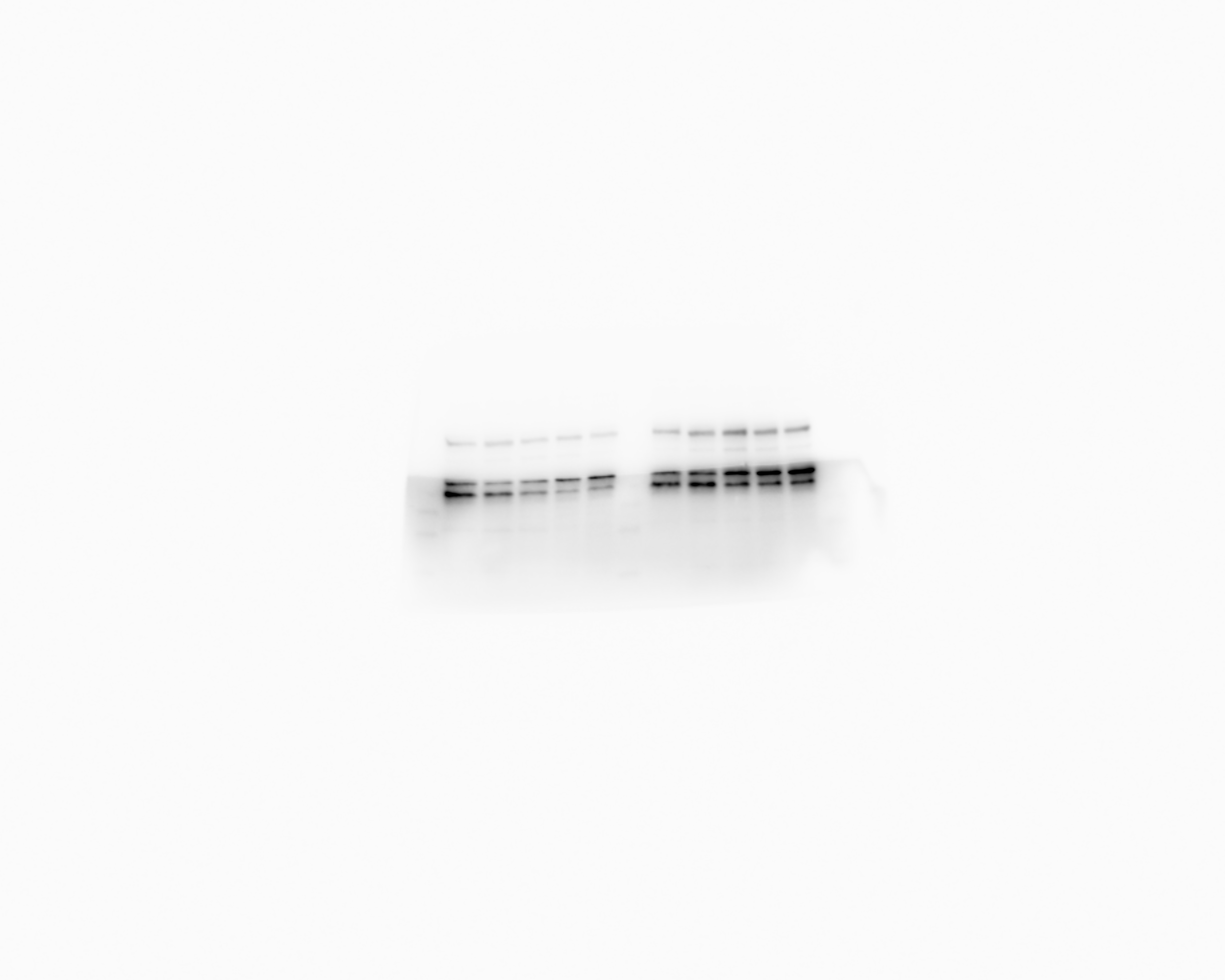

Supplement: Figure 5—source data 1. [file elife-84238-fig5-data1.zip › z Figure 5-Source Data 1/Figure 5-Source Data 1/Fig 5G/IkBa.jpg]

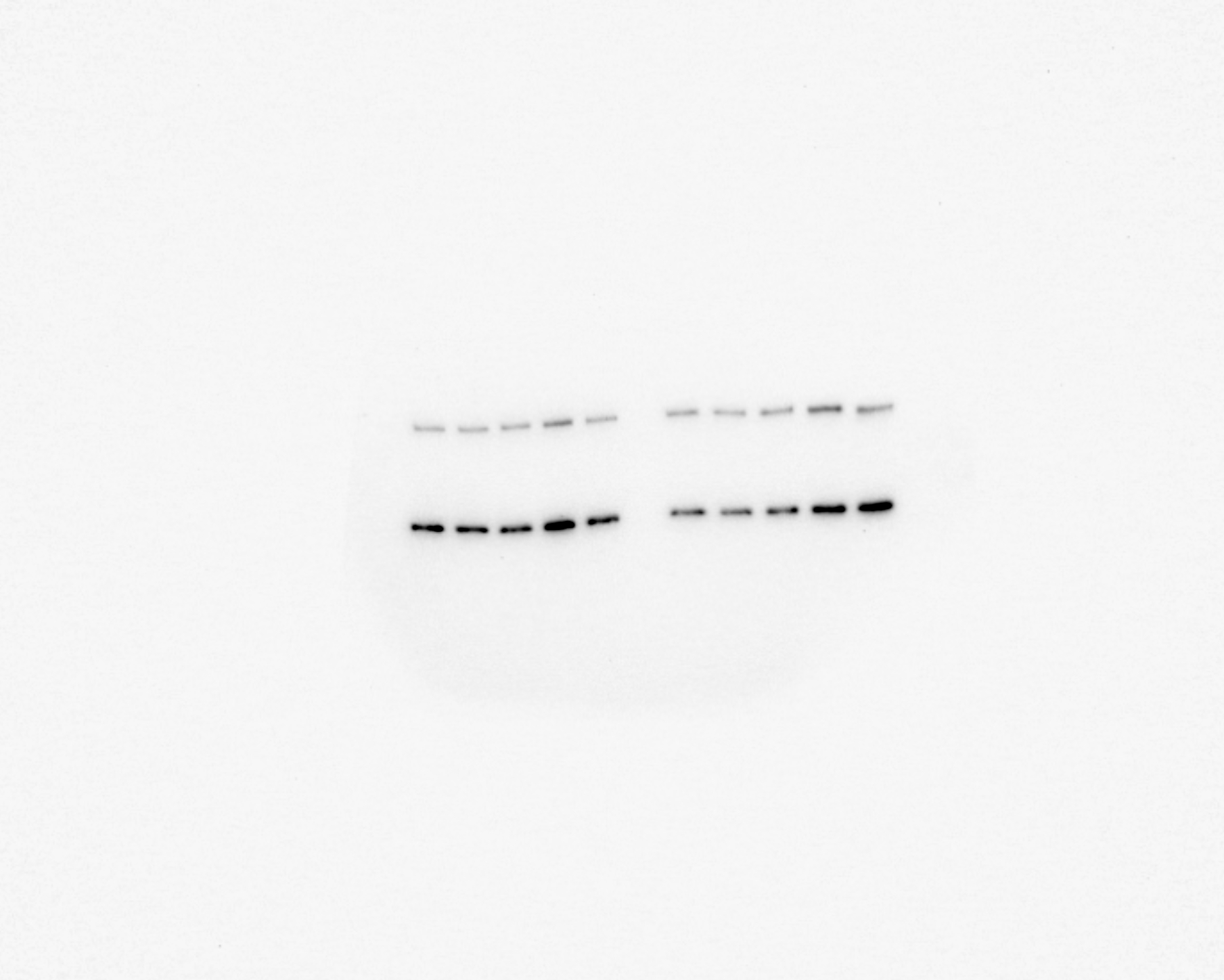

Supplement: Figure 5—source data 1. [file elife-84238-fig5-data1.zip › z Figure 5-Source Data 1/Figure 5-Source Data 1/Fig 5G/p105.p50.jpg]

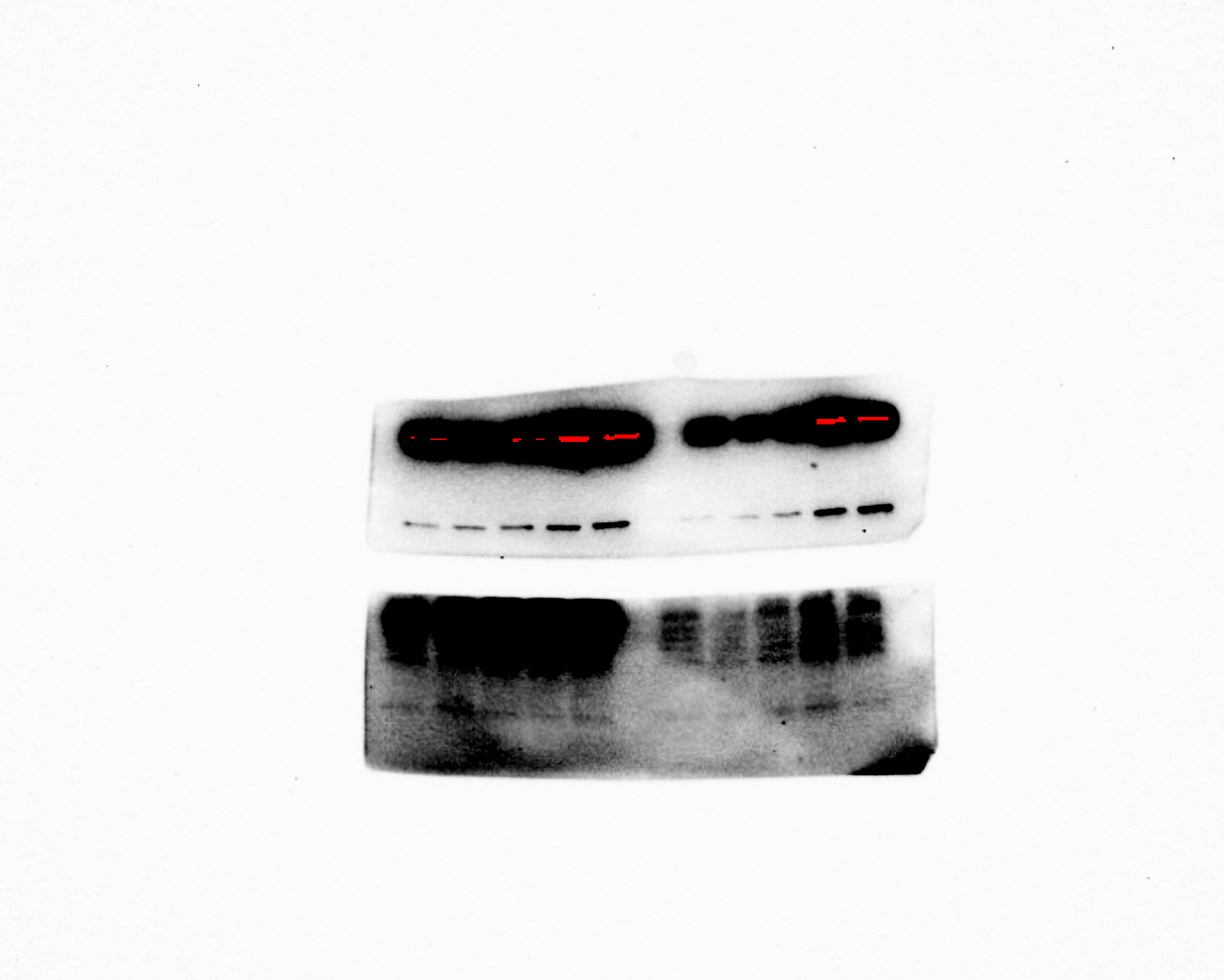

Supplement: Figure 5—source data 1. [file elife-84238-fig5-data1.zip › z Figure 5-Source Data 1/Figure 5-Source Data 1/Fig 5G/p52 .jpg]

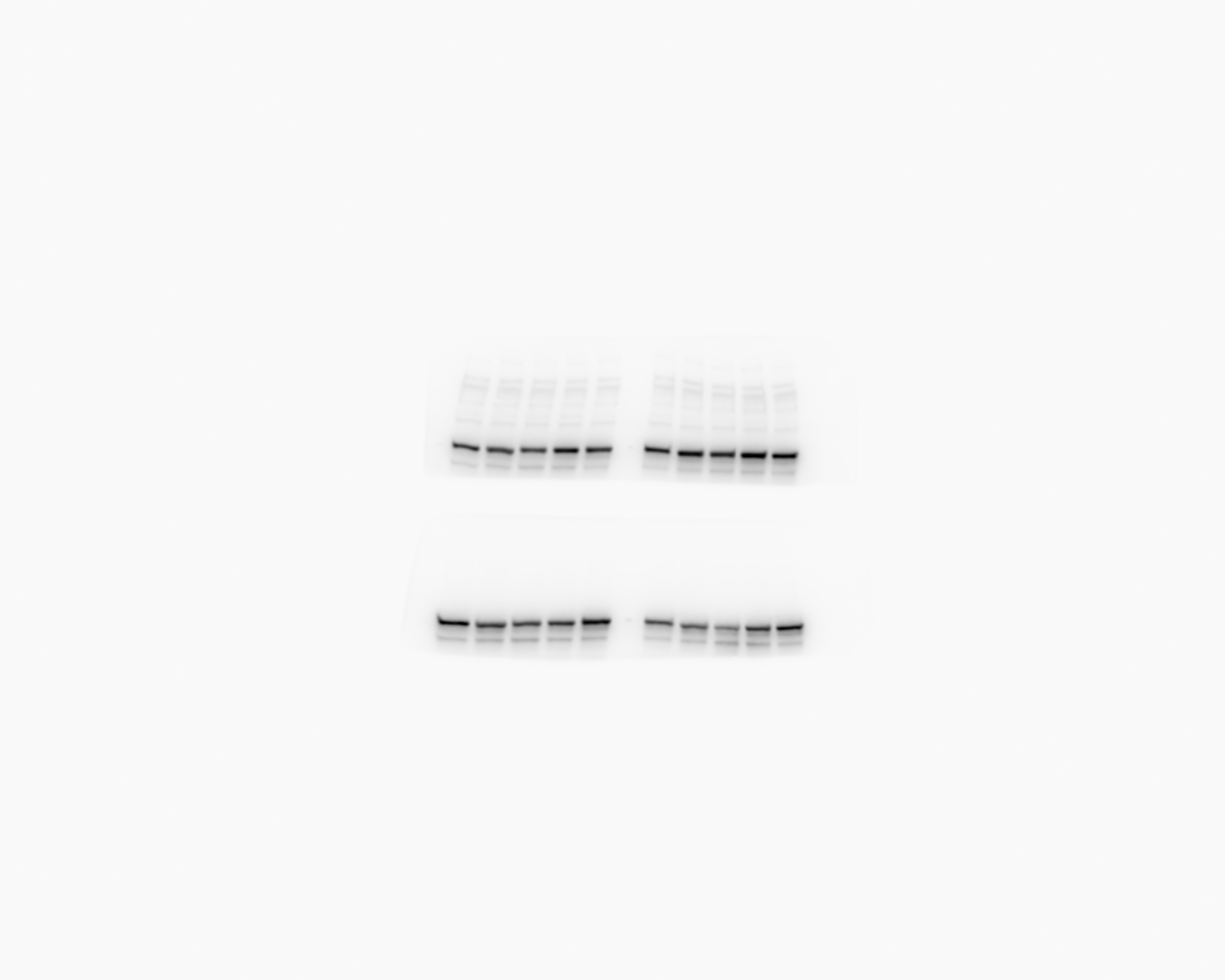

Supplement: Figure 5—source data 1. [file elife-84238-fig5-data1.zip › z Figure 5-Source Data 1/Figure 5-Source Data 1/Fig 5G/p65.jpg]

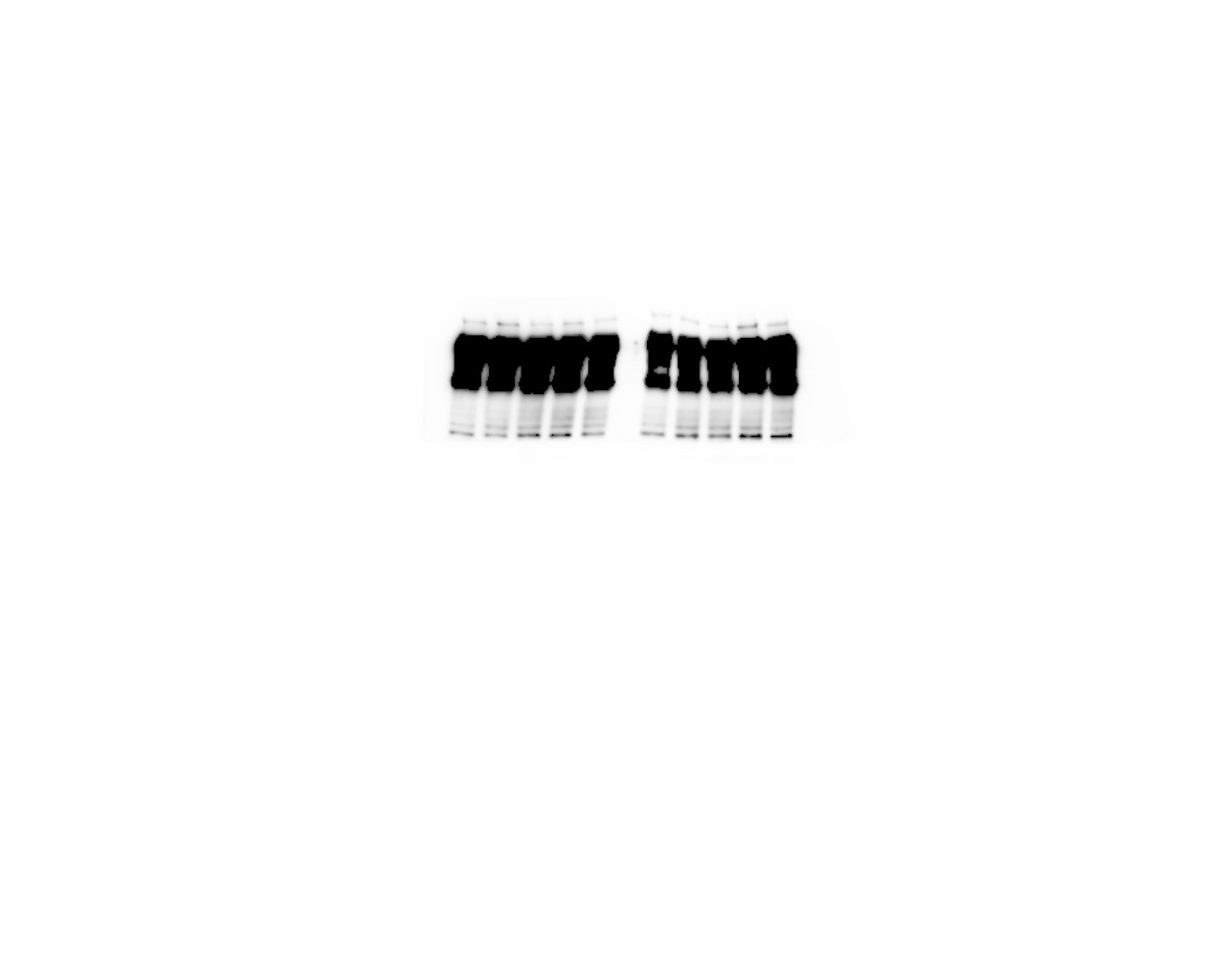

Supplement: Figure 5—source data 1. [file elife-84238-fig5-data1.zip › z Figure 5-Source Data 1/Figure 5-Source Data 1/Fig 5G/phospho-p65.jpg]

Figure 5A

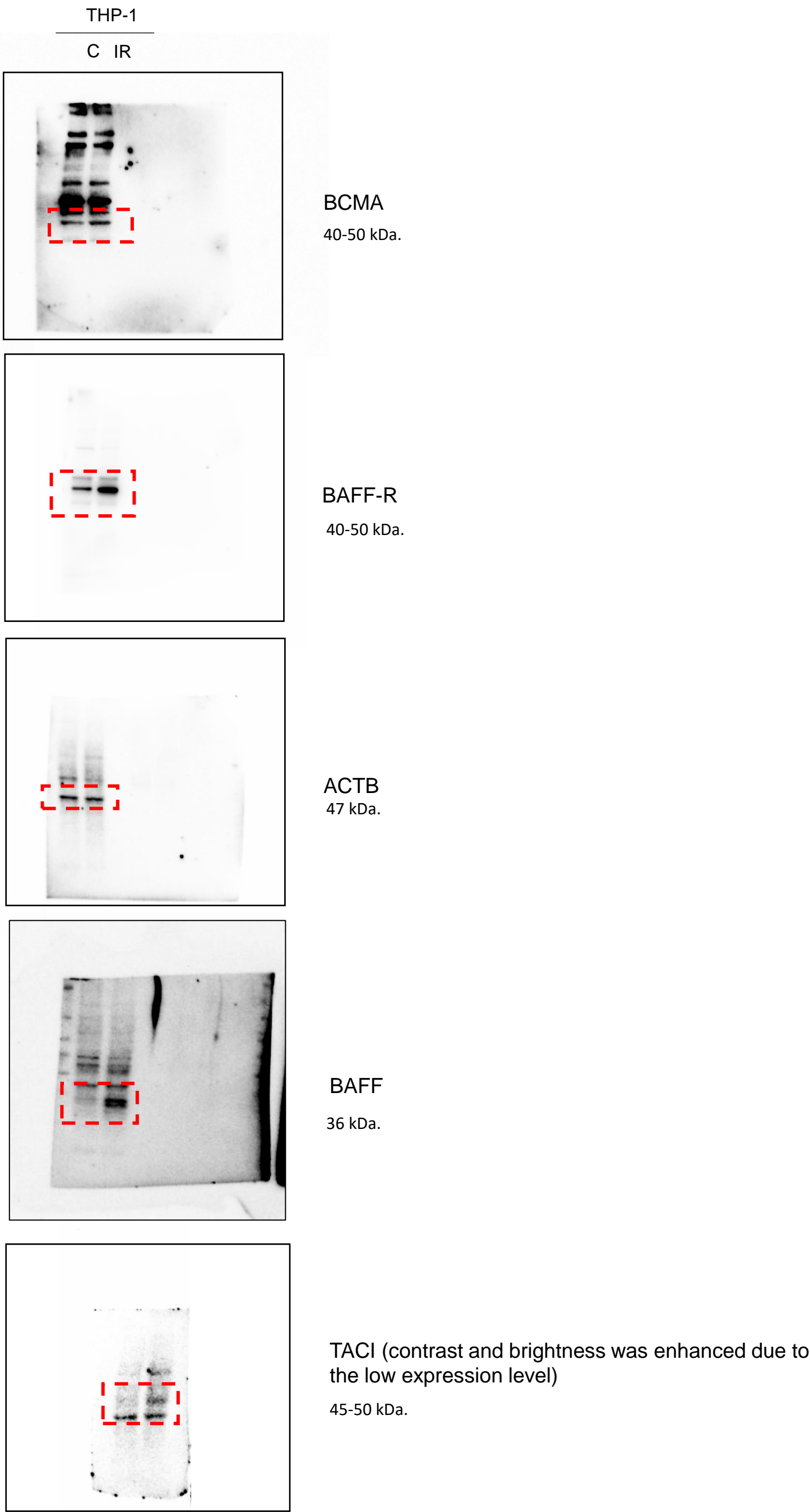

Figure 5F

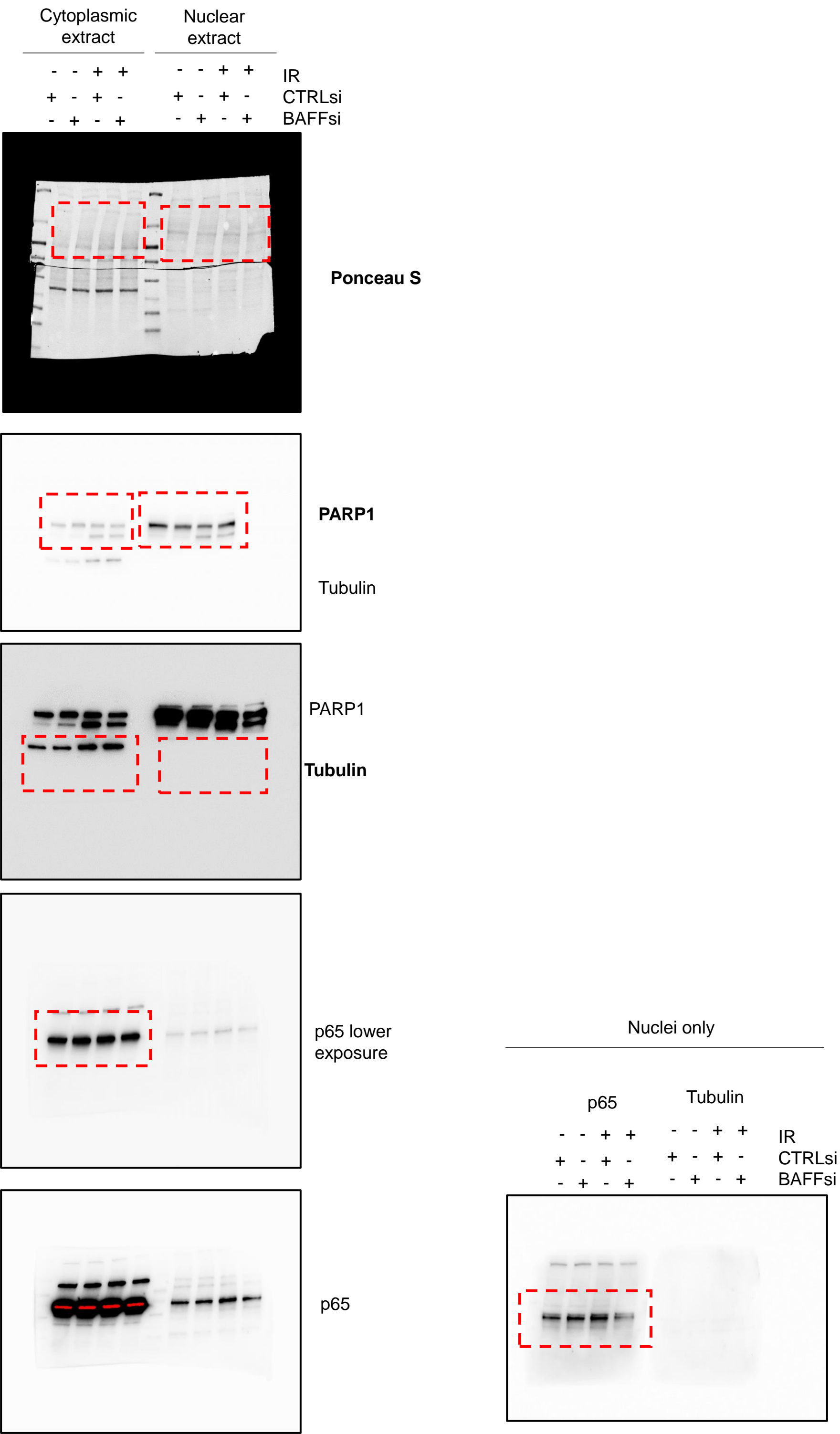

Figure 5G

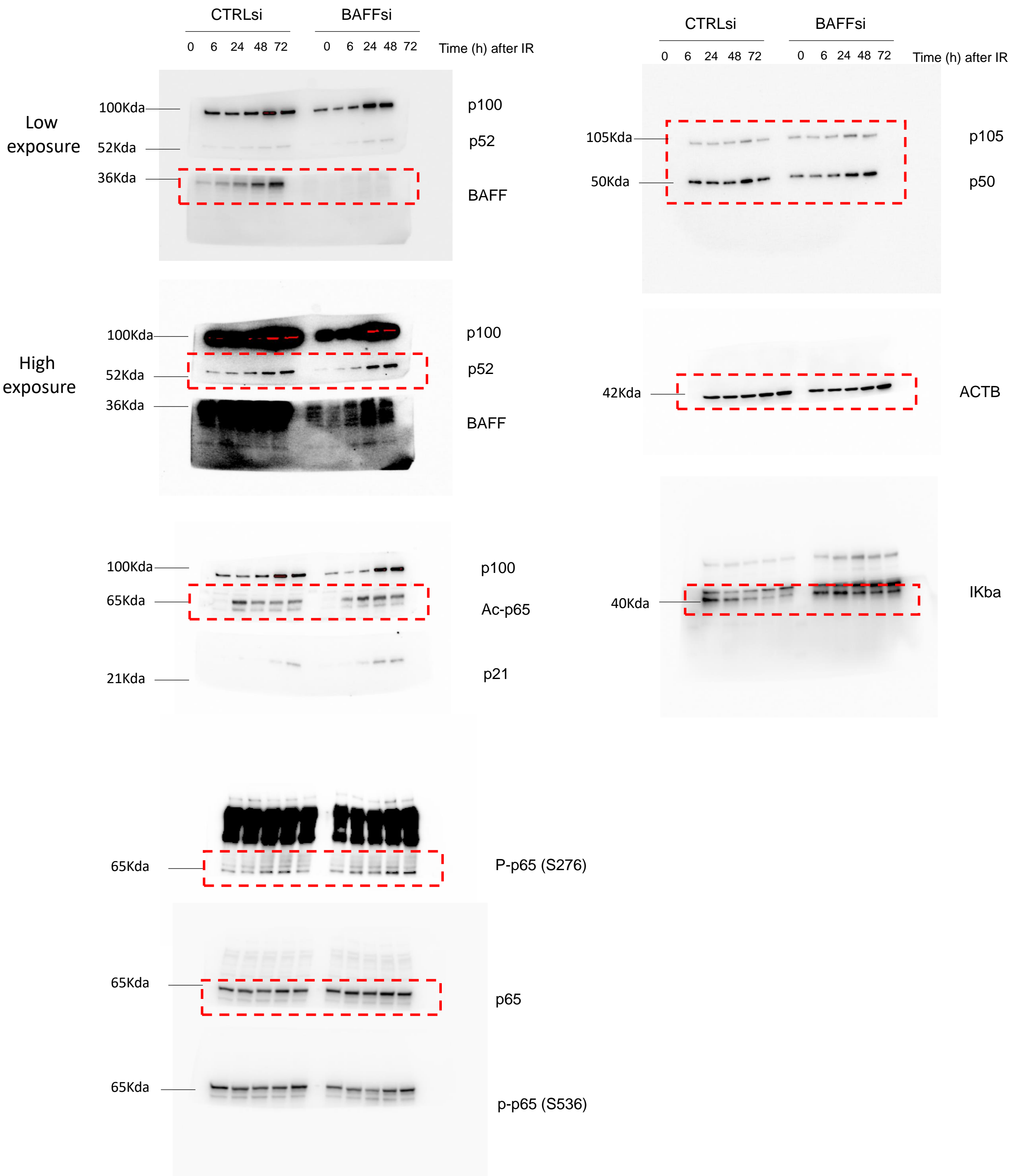

Supplement: Figure 5—source data 1. [file elife-84238-fig5-data1.zip › z Figure 5-Source Data 1/Figure 5-Source Data 1/Figure 5 uncropped blots.pdf]

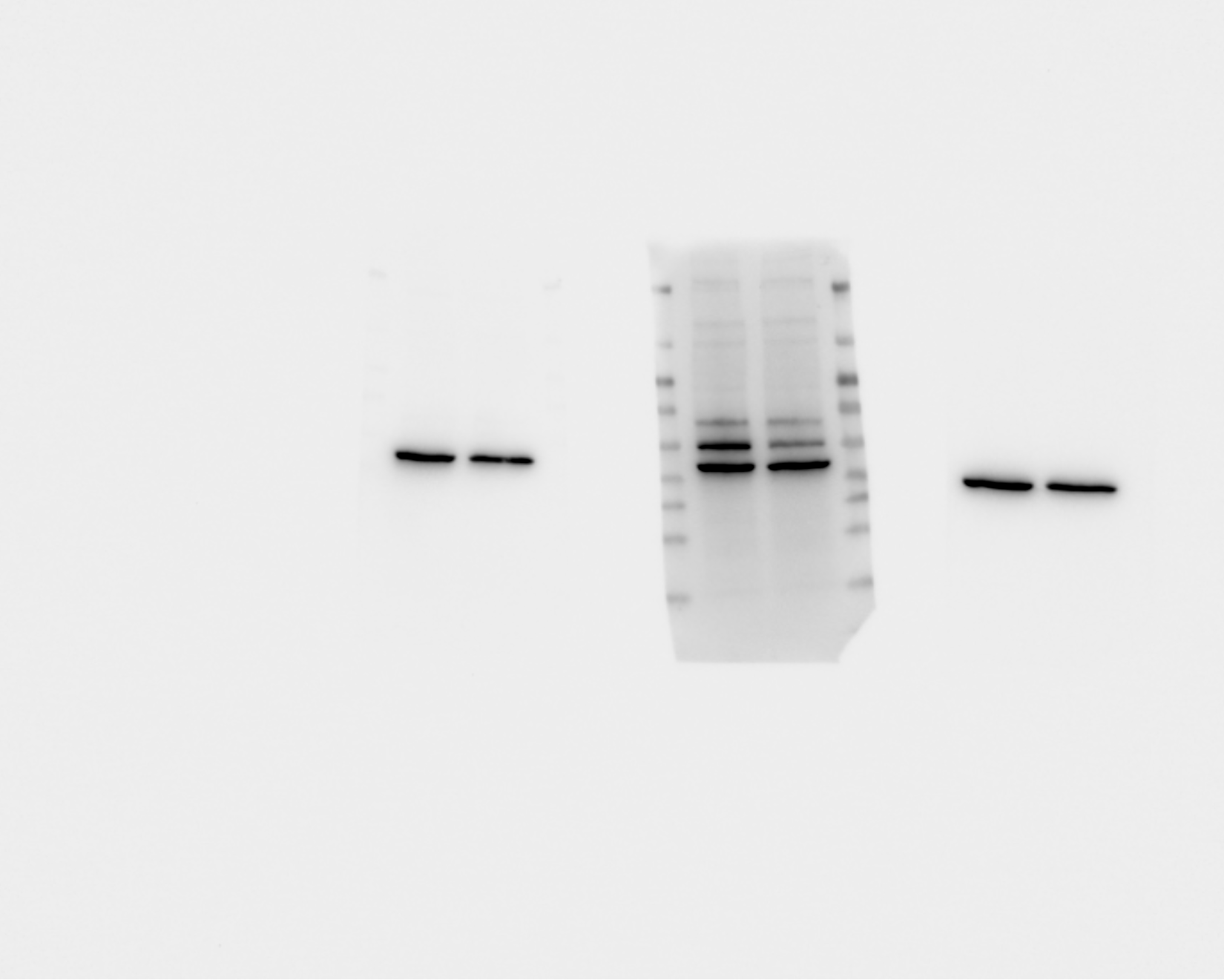

Supplement: Figure 5—figure supplement 1—source data 1. [file elife-84238-fig5-figsupp1-data1.zip › z Figure 5-Figure supplement 1-Source Data 1/Figure 5-Figure Supplement 1-Source Data 1/Fig 5SA/Actin.tif]

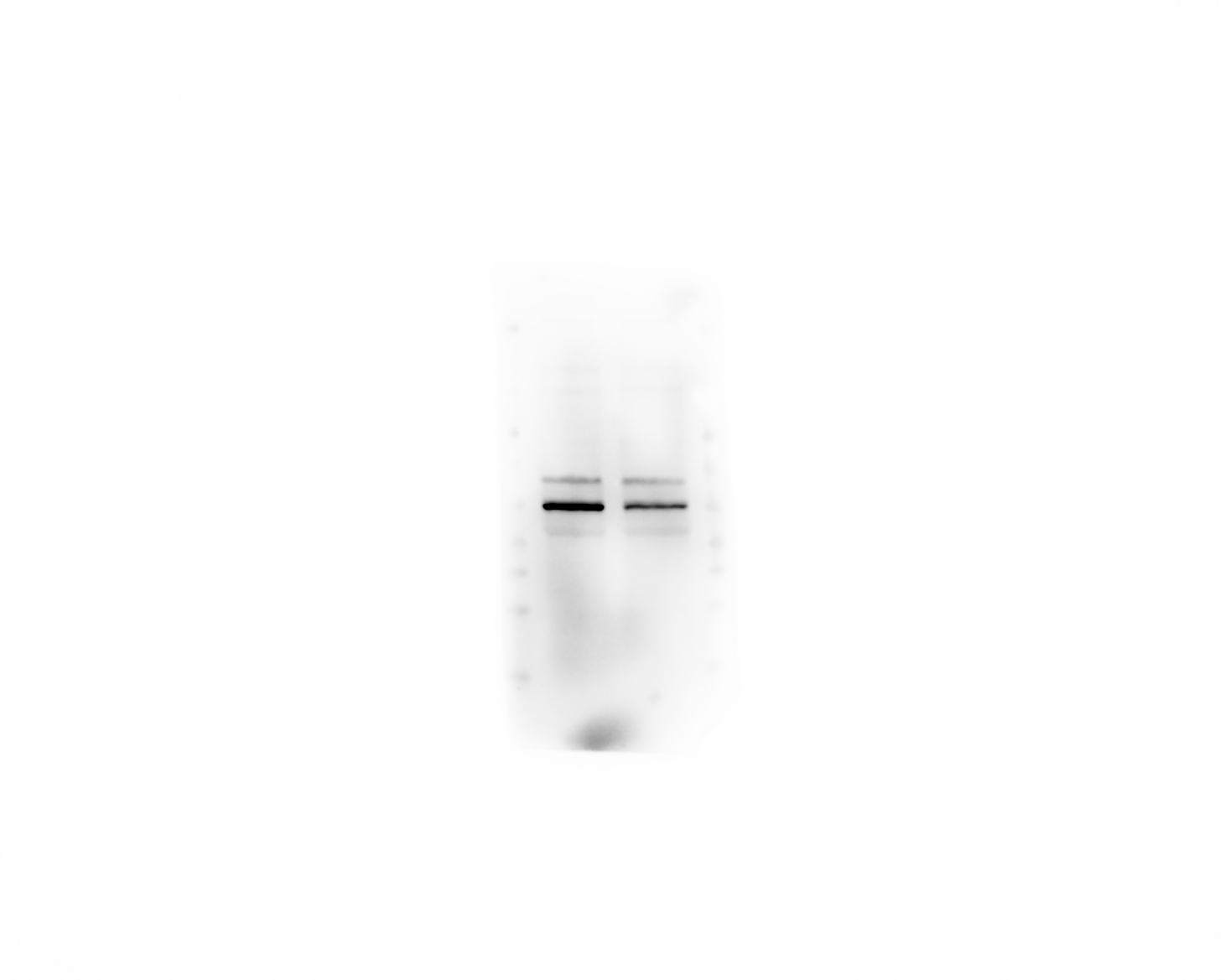

Supplement: Figure 5—figure supplement 1—source data 1. [file elife-84238-fig5-figsupp1-data1.zip › z Figure 5-Figure supplement 1-Source Data 1/Figure 5-Figure Supplement 1-Source Data 1/Fig 5SA/BAFFRsi THP.tif]

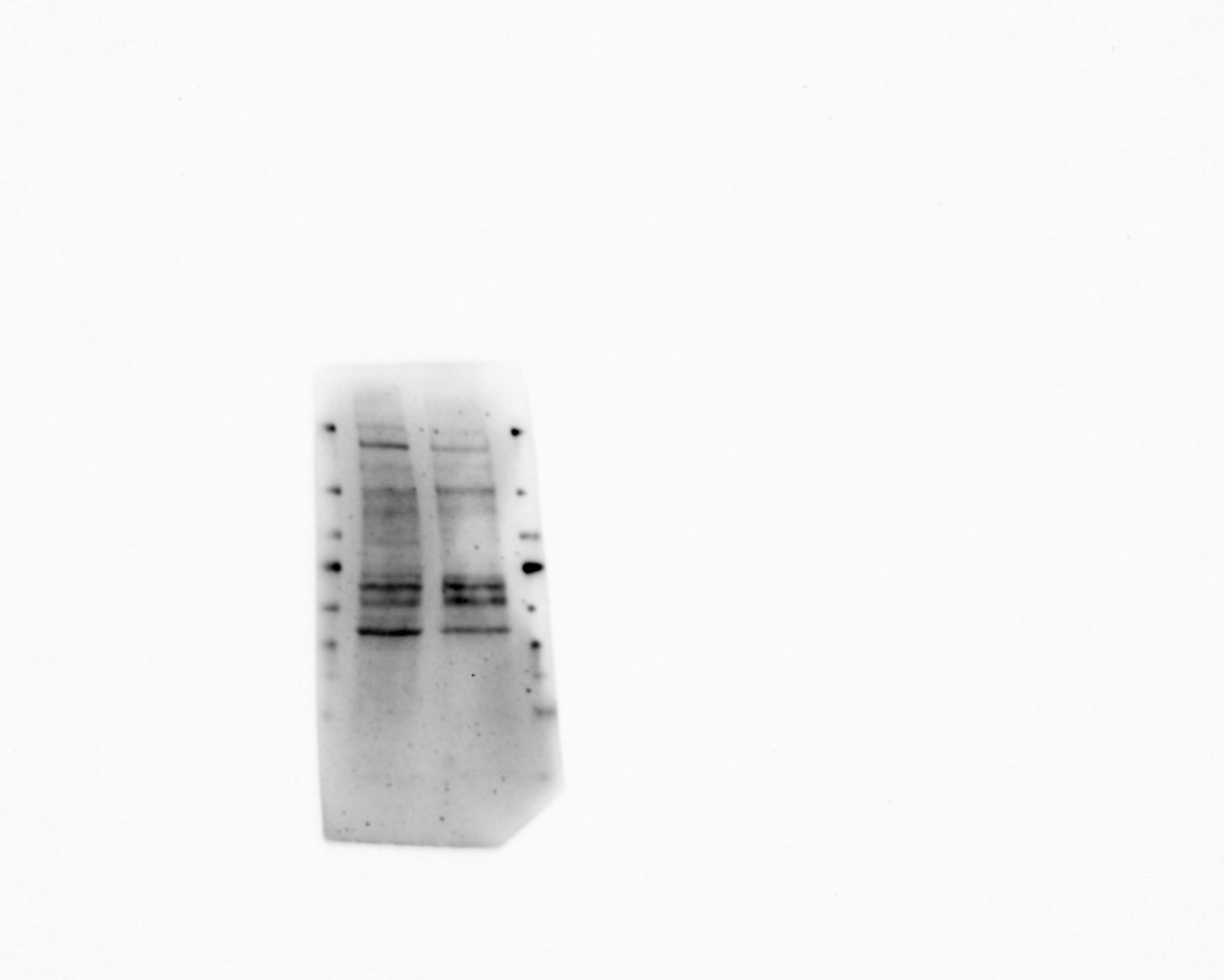

Supplement: Figure 5—figure supplement 1—source data 1. [file elife-84238-fig5-figsupp1-data1.zip › z Figure 5-Figure supplement 1-Source Data 1/Figure 5-Figure Supplement 1-Source Data 1/Fig 5SA/BCMA THP1.tif]

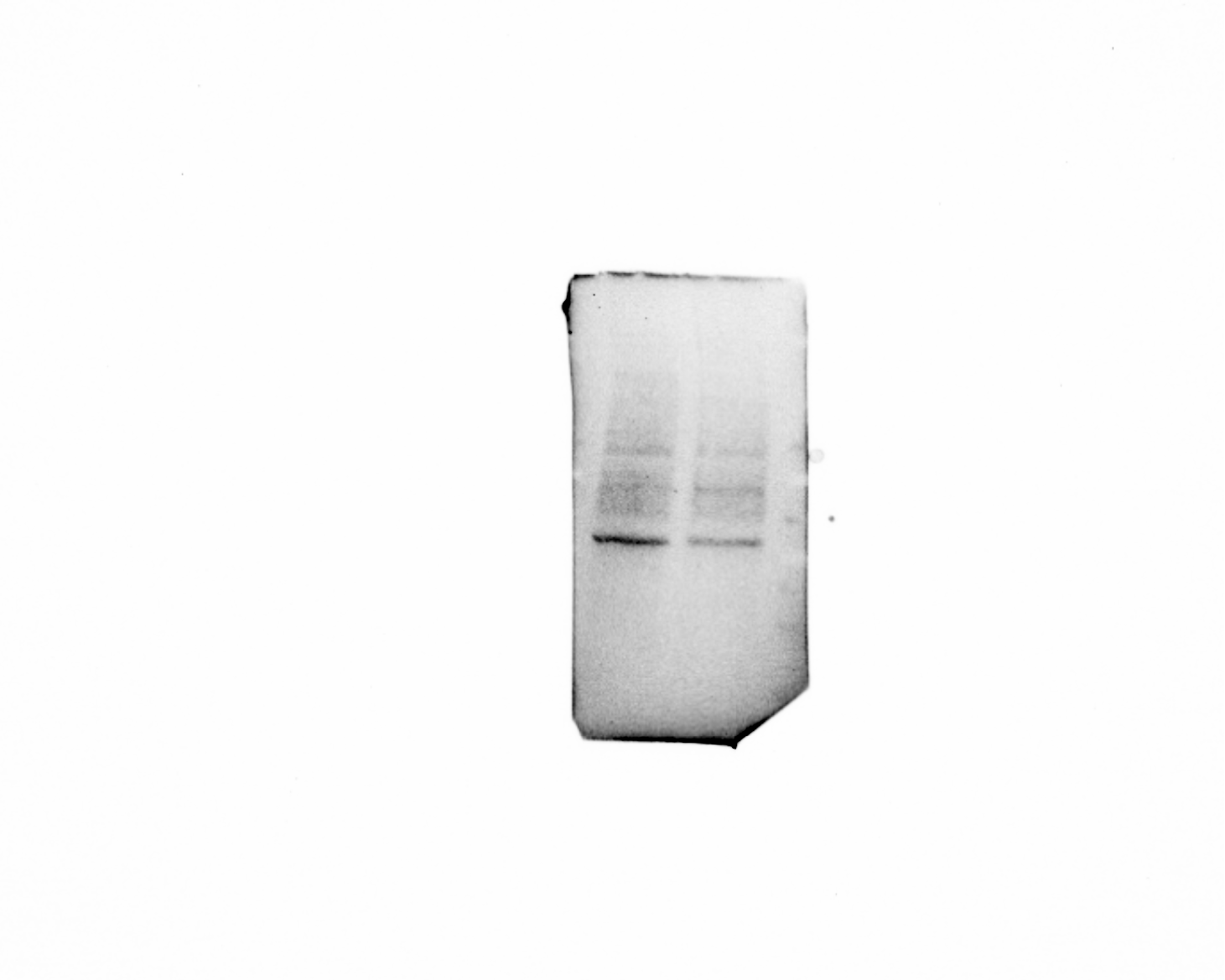

Supplement: Figure 5—figure supplement 1—source data 1. [file elife-84238-fig5-figsupp1-data1.zip › z Figure 5-Figure supplement 1-Source Data 1/Figure 5-Figure Supplement 1-Source Data 1/Fig 5SA/TACIsi THP.tif]

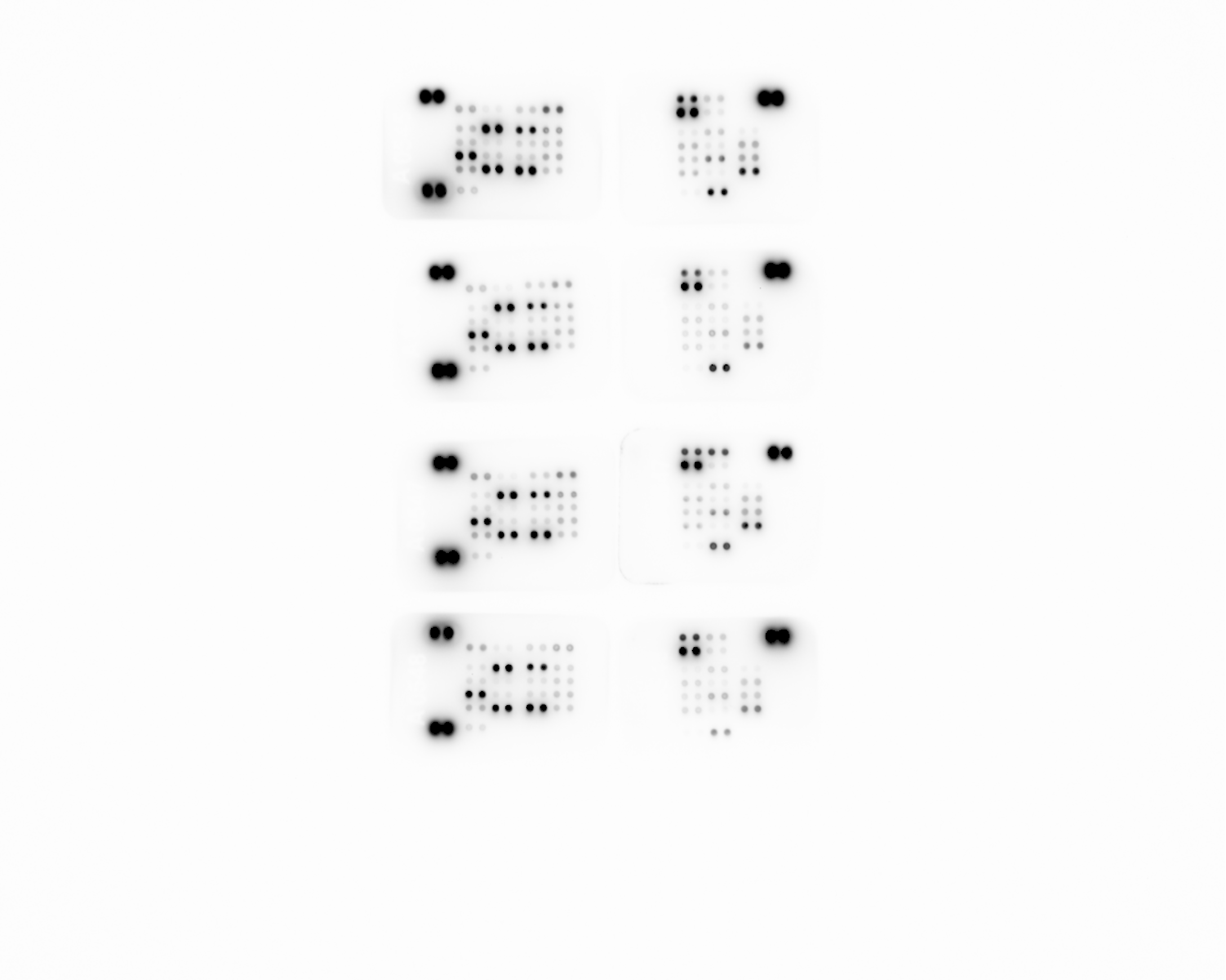

Supplement: Figure 5—figure supplement 1—source data 1. [file elife-84238-fig5-figsupp1-data1.zip › z Figure 5-Figure supplement 1-Source Data 1/Figure 5-Figure Supplement 1-Source Data 1/Fig 5SD/replicate 1 and 2.jpeg]

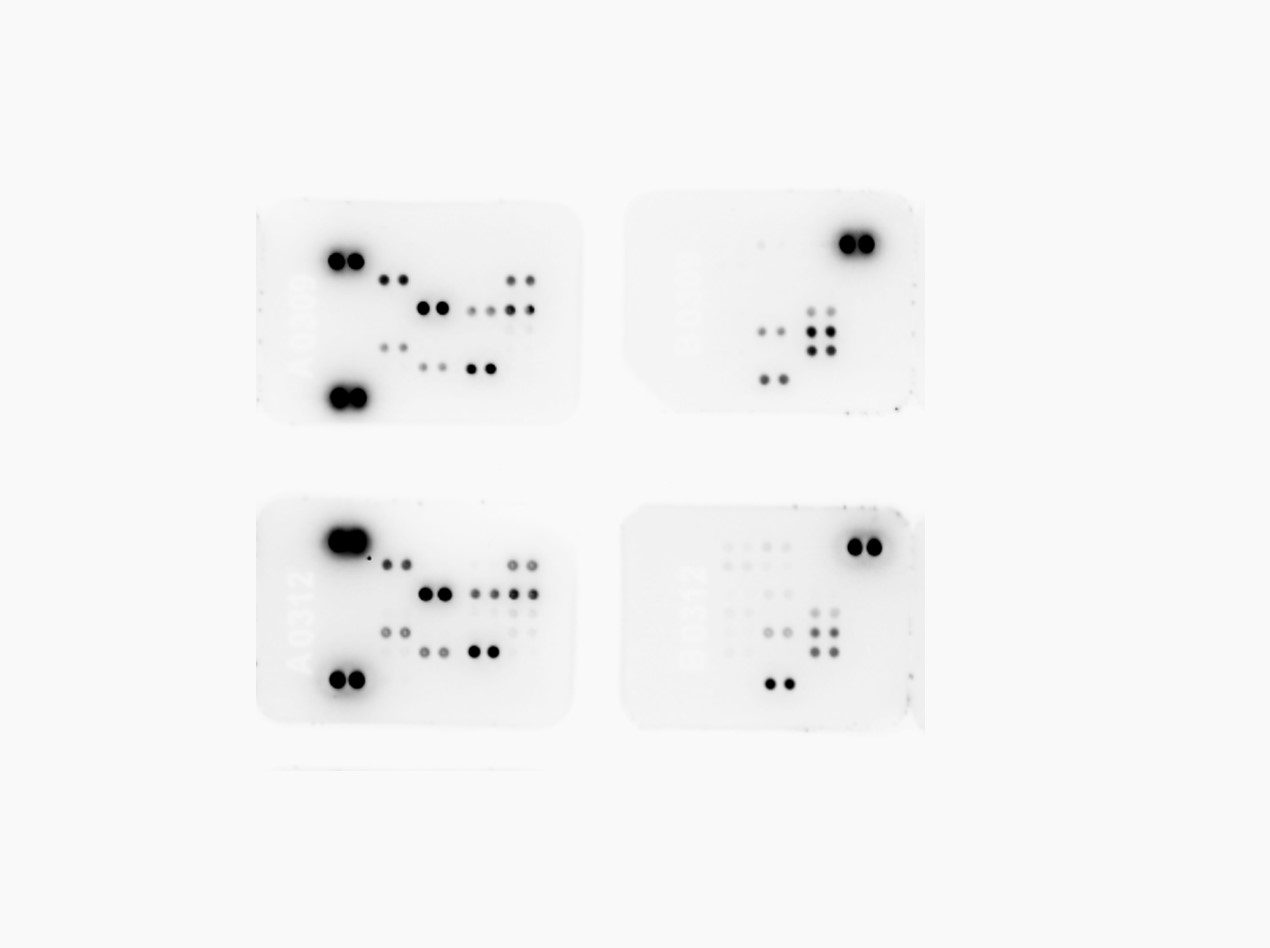

Supplement: Figure 5—figure supplement 1—source data 1. [file elife-84238-fig5-figsupp1-data1.zip › z Figure 5-Figure supplement 1-Source Data 1/Figure 5-Figure Supplement 1-Source Data 1/Fig 5SD/Replicate 3 edited for correct orientation.jpg]

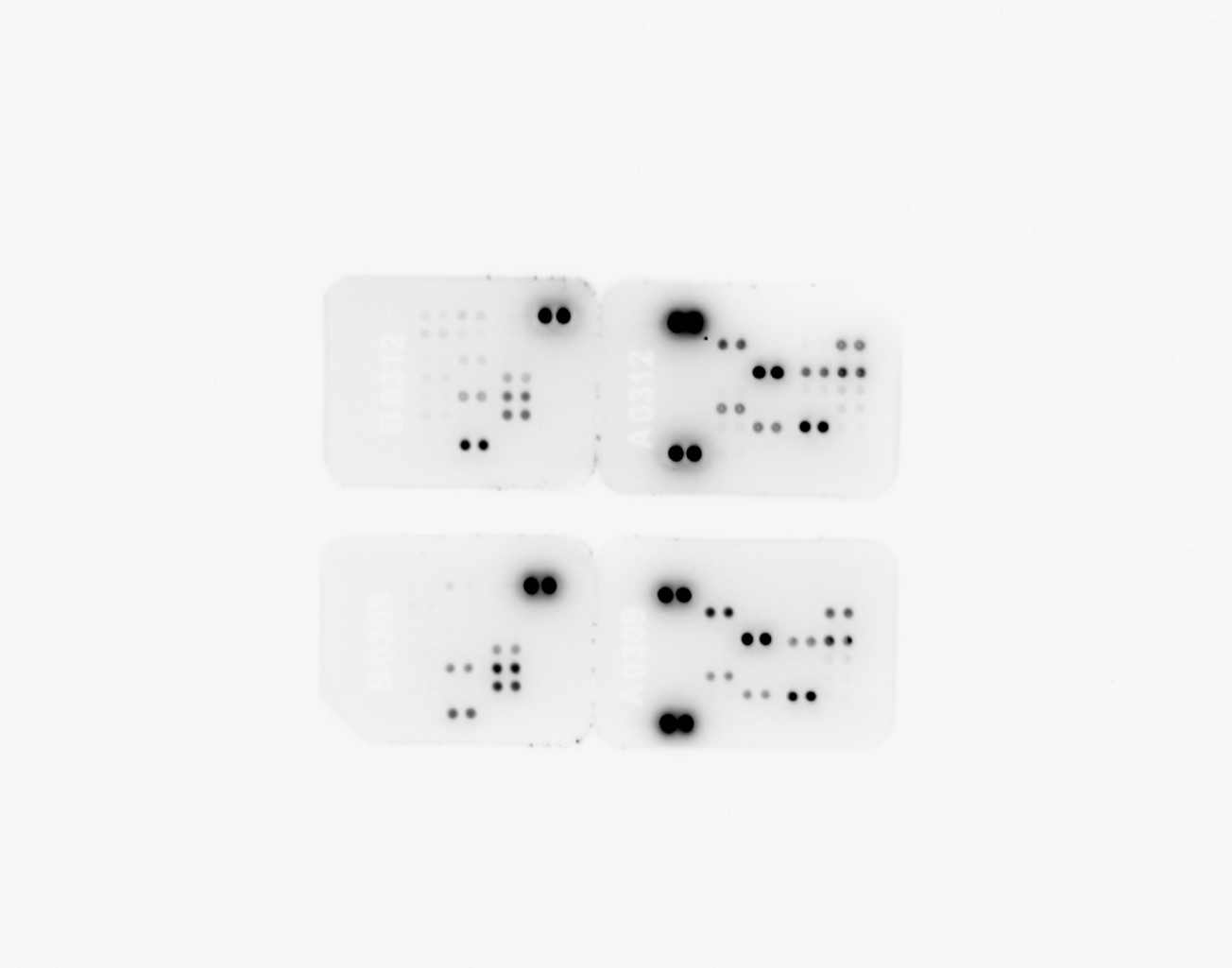

Supplement: Figure 5—figure supplement 1—source data 1. [file elife-84238-fig5-figsupp1-data1.zip › z Figure 5-Figure supplement 1-Source Data 1/Figure 5-Figure Supplement 1-Source Data 1/Fig 5SD/Replicate 3 unedited.jpg]

S5A

THP-1

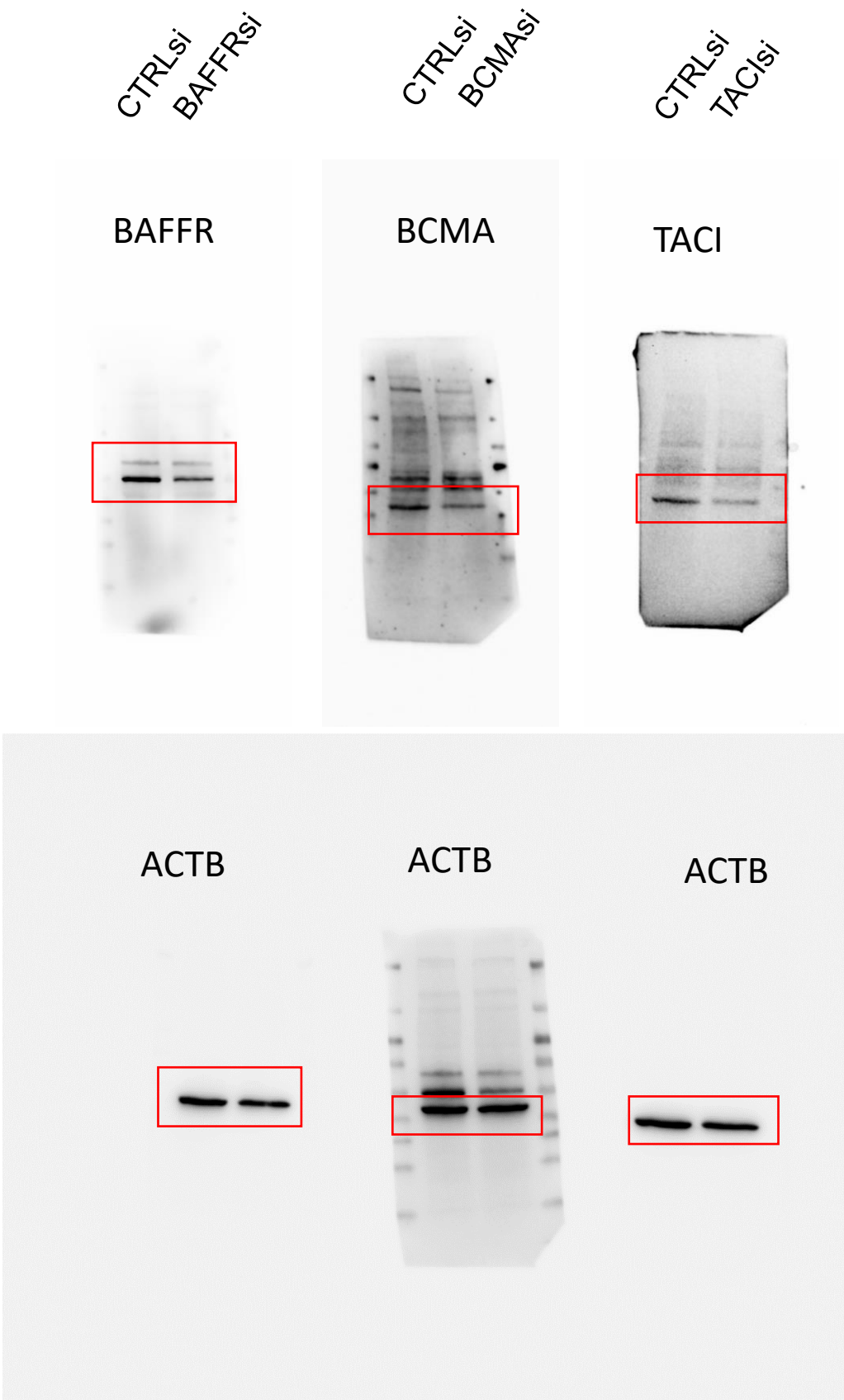

Replicate 1

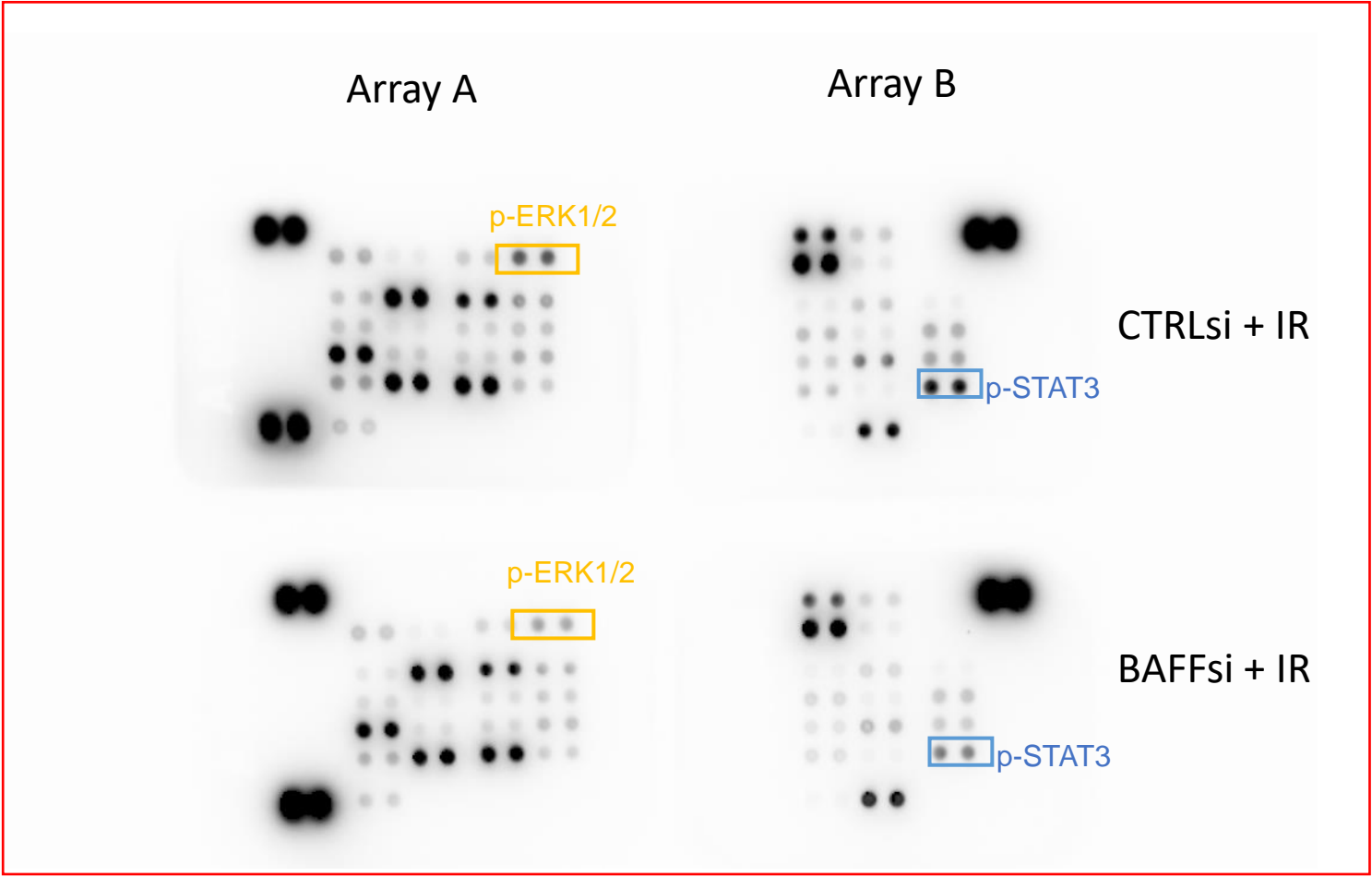

Replicate 2

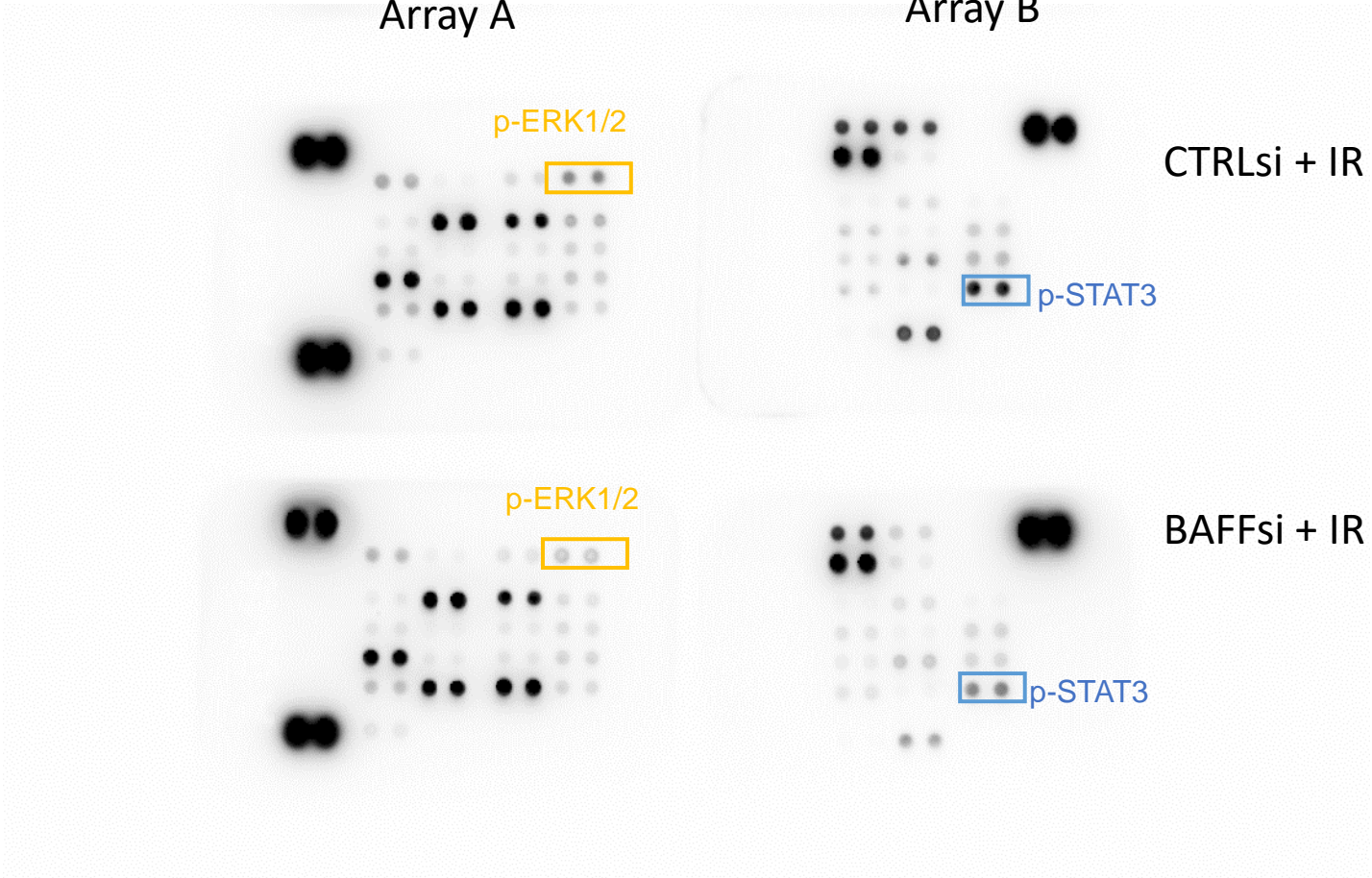

Replicate 3

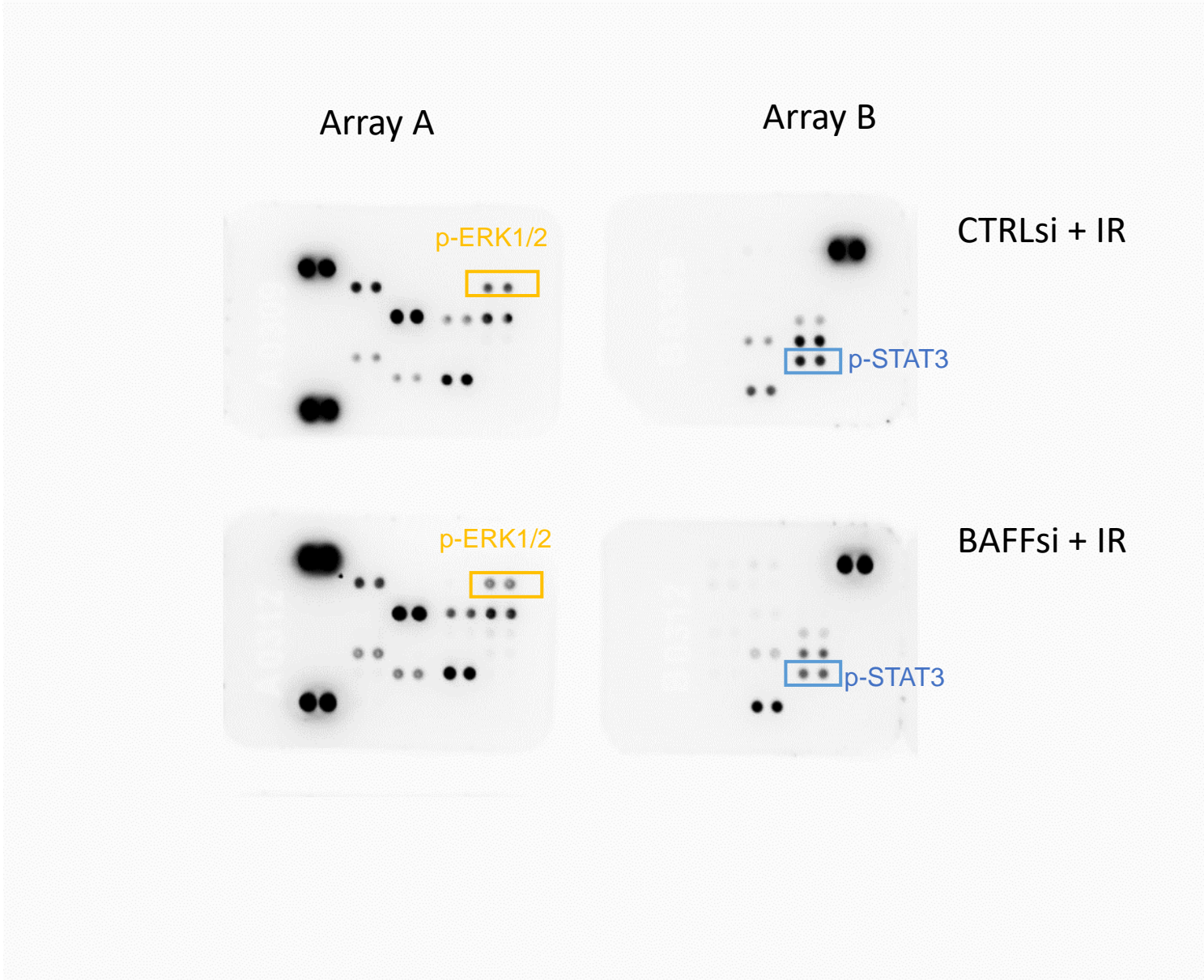

Supplement: Figure 5—figure supplement 1—source data 1. [file elife-84238-fig5-figsupp1-data1.zip › z Figure 5-Figure supplement 1-Source Data 1/Figure 5-Figure Supplement 1-Source Data 1/Figure 5S uncropped blots.pdf]

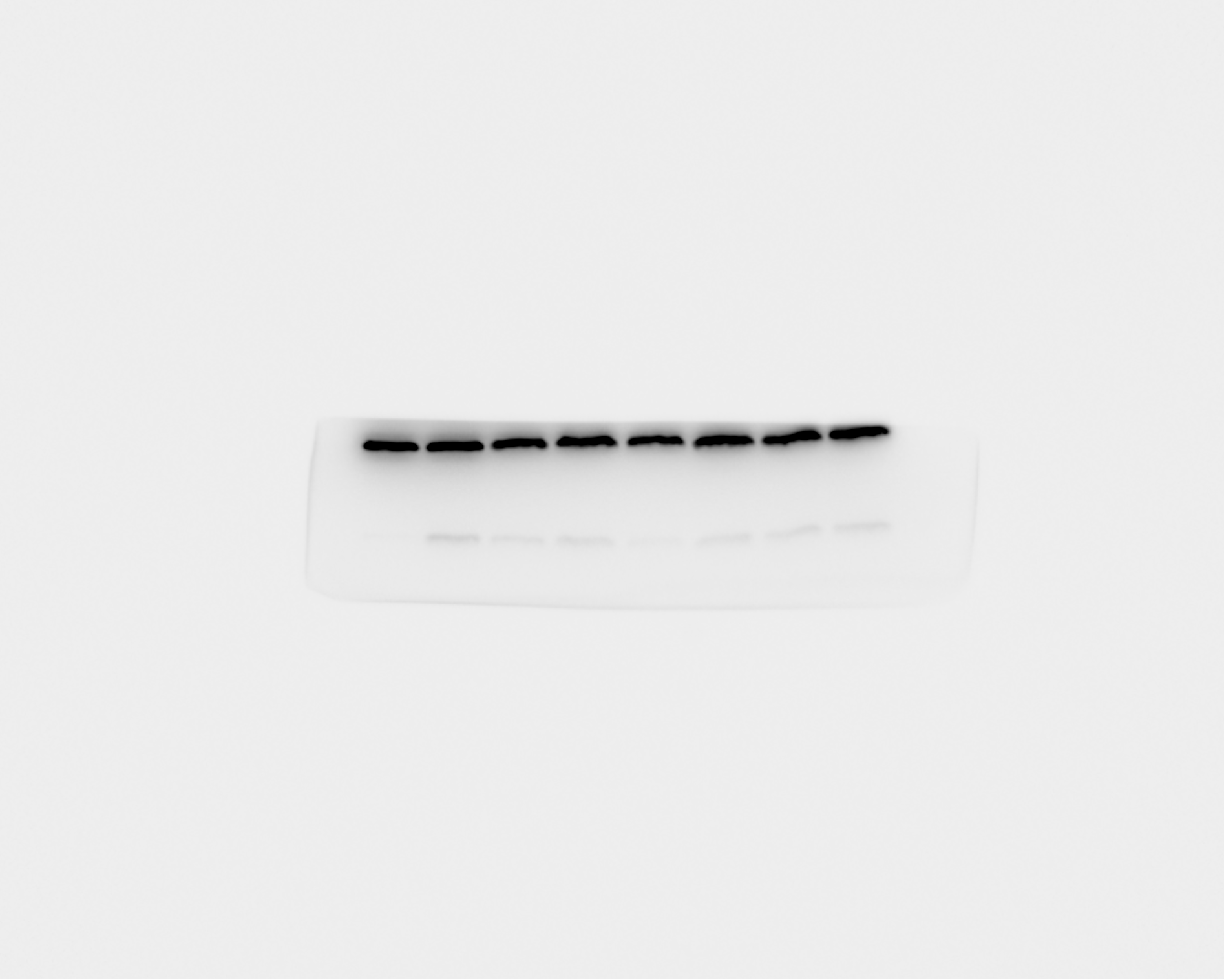

Supplement: Figure 6—figure supplement 1—source data 1. [file elife-84238-fig6-figsupp1-data1.zip › z Figure 6-Figure Supplement 1-Source Data 1/Figure 6-Figure Supplement 1-Source Data 1/original pics/ACTB.jpg]

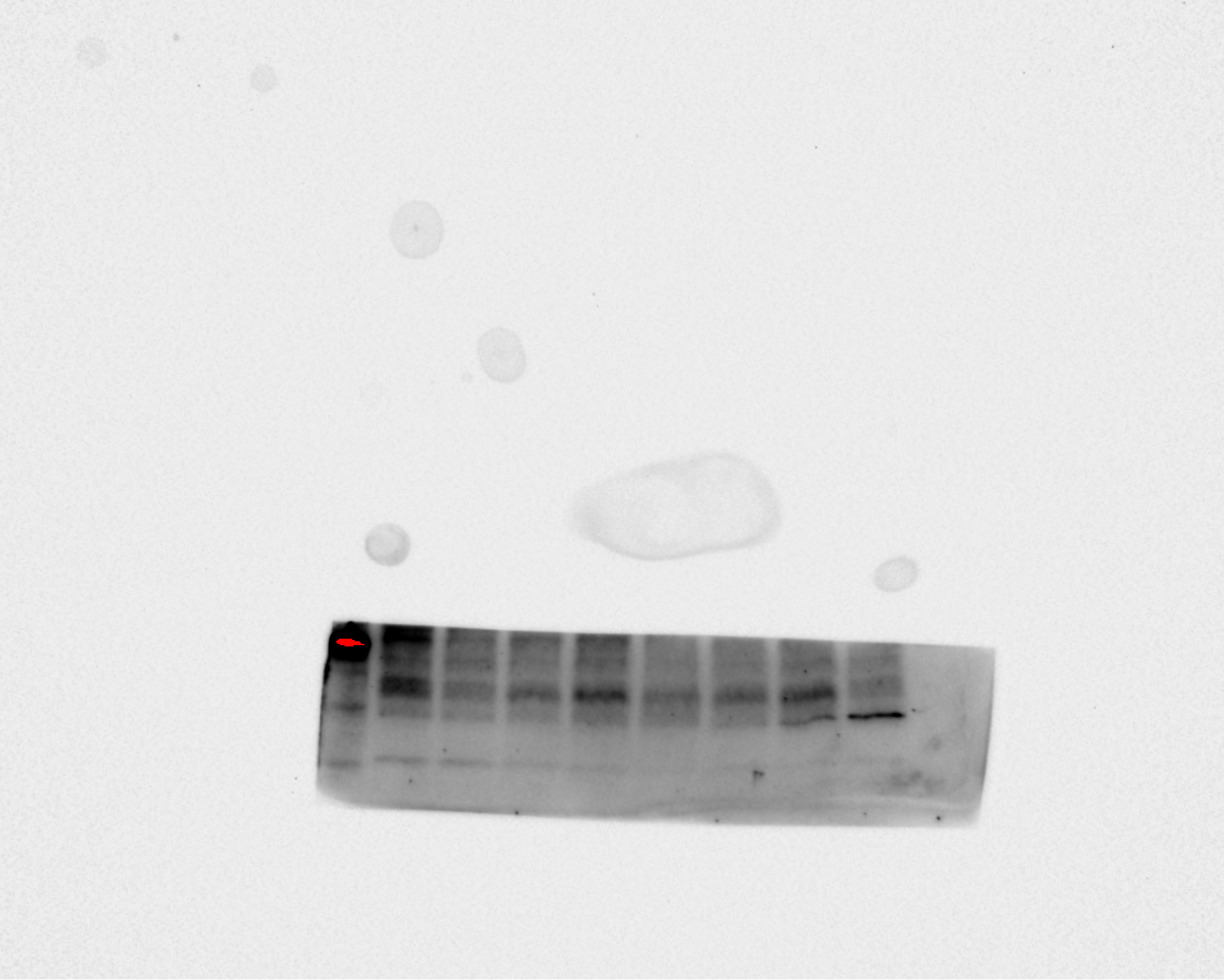

Supplement: Figure 6—figure supplement 1—source data 1. [file elife-84238-fig6-figsupp1-data1.zip › z Figure 6-Figure Supplement 1-Source Data 1/Figure 6-Figure Supplement 1-Source Data 1/original pics/BAFF.jpg]

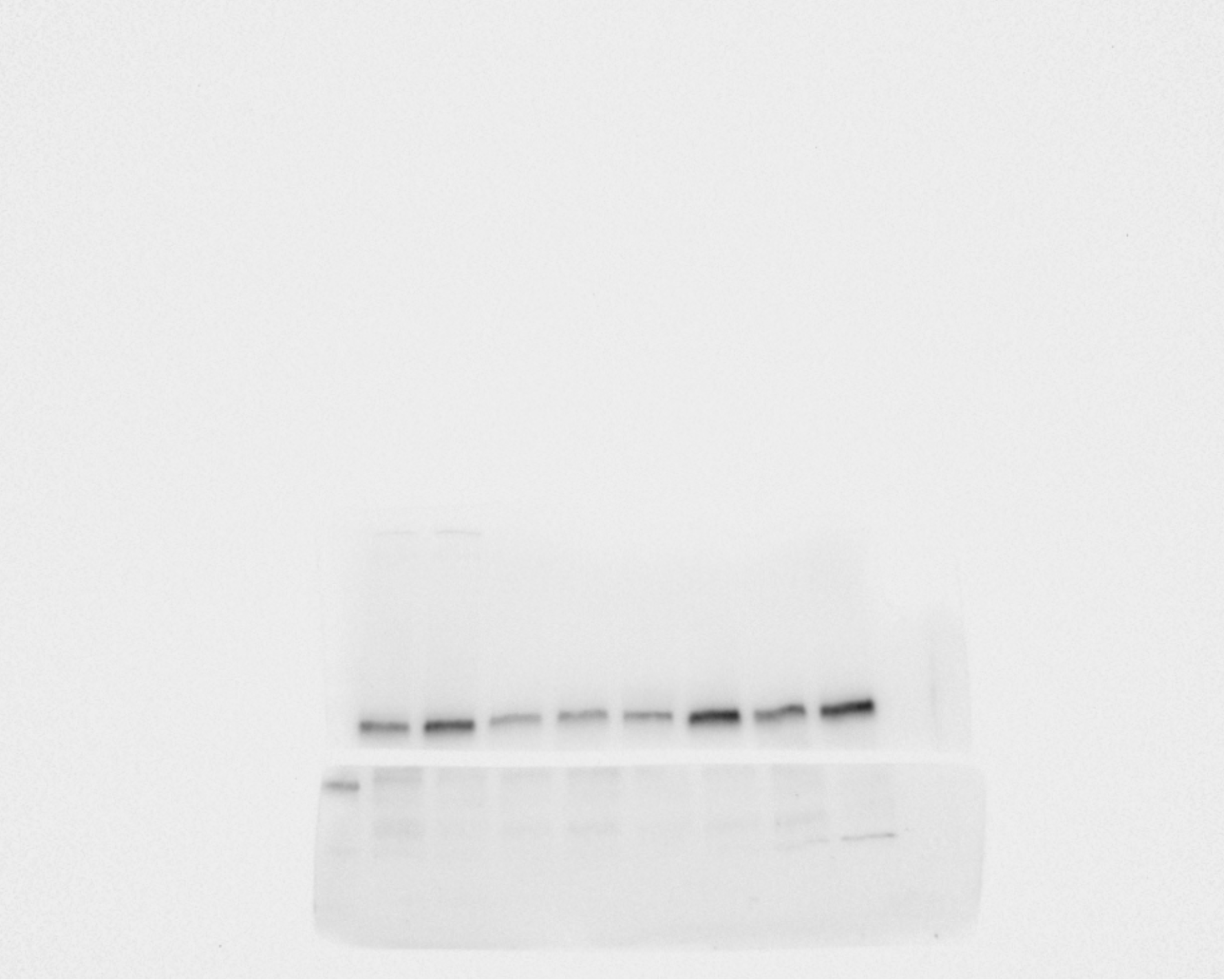

Supplement: Figure 6—figure supplement 1—source data 1. [file elife-84238-fig6-figsupp1-data1.zip › z Figure 6-Figure Supplement 1-Source Data 1/Figure 6-Figure Supplement 1-Source Data 1/original pics/p53.jpg]

Figure 6-Figure Supplement 1

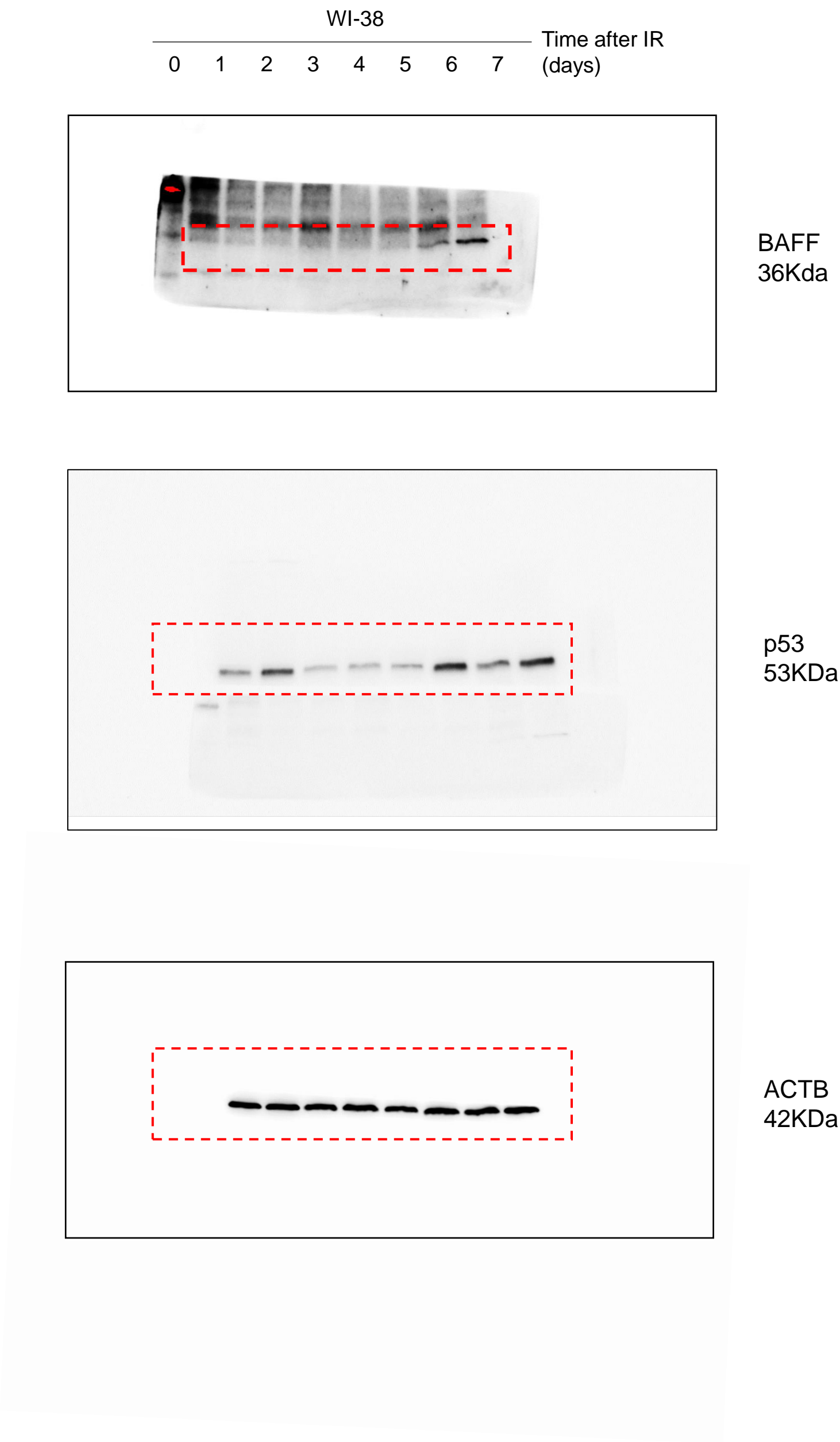

Supplement: Figure 6—figure supplement 1—source data 1. [file elife-84238-fig6-figsupp1-data1.zip › z Figure 6-Figure Supplement 1-Source Data 1/Figure 6-Figure Supplement 1-Source Data 1/uncropped blots S6.pdf]

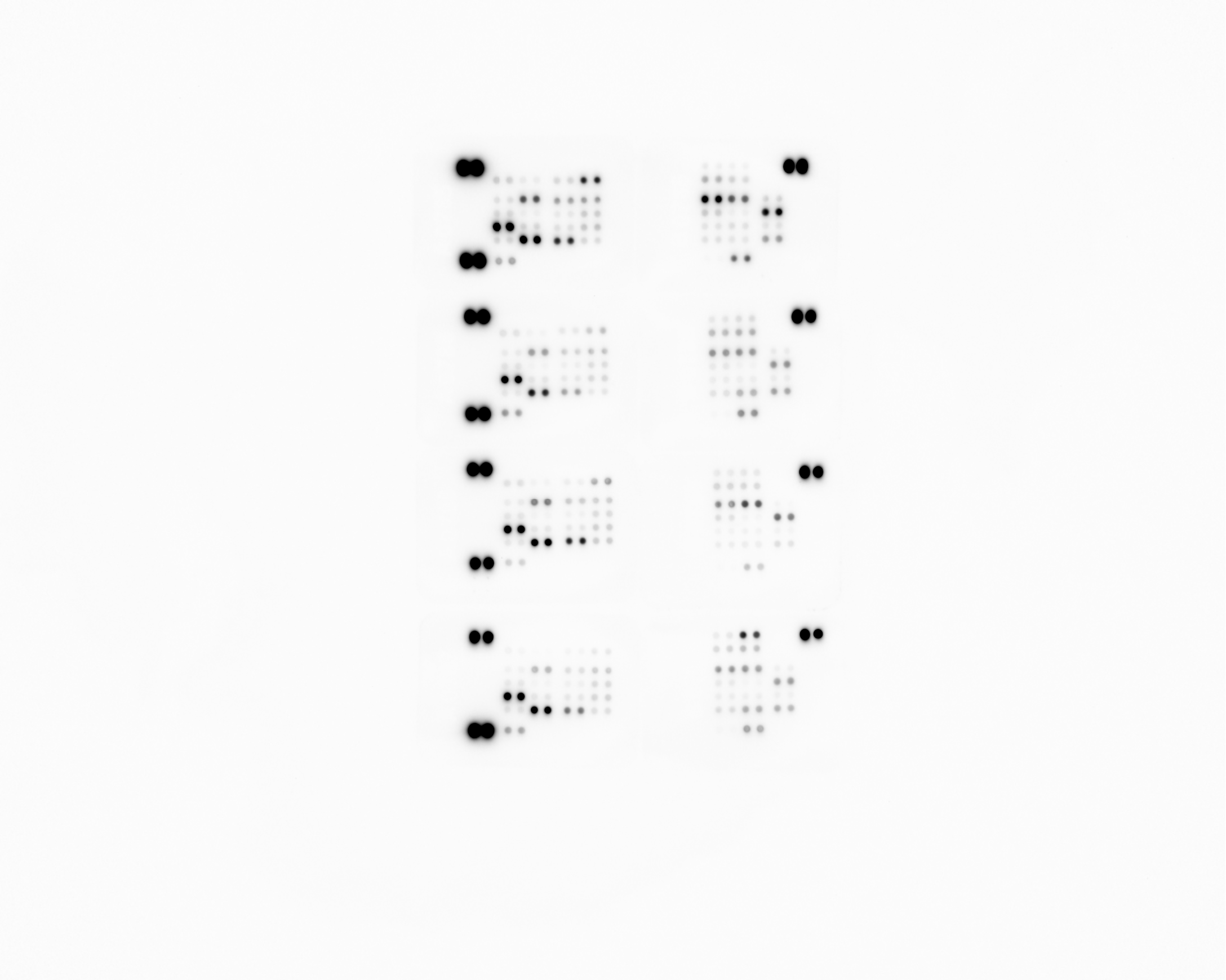

Supplement: Figure 7—source data 1. [file elife-84238-fig7-data1.zip › z Figure 7-Source Data 1/Figure 7-Source Data 1/original files/7A/rep 2 and 3.jpg]

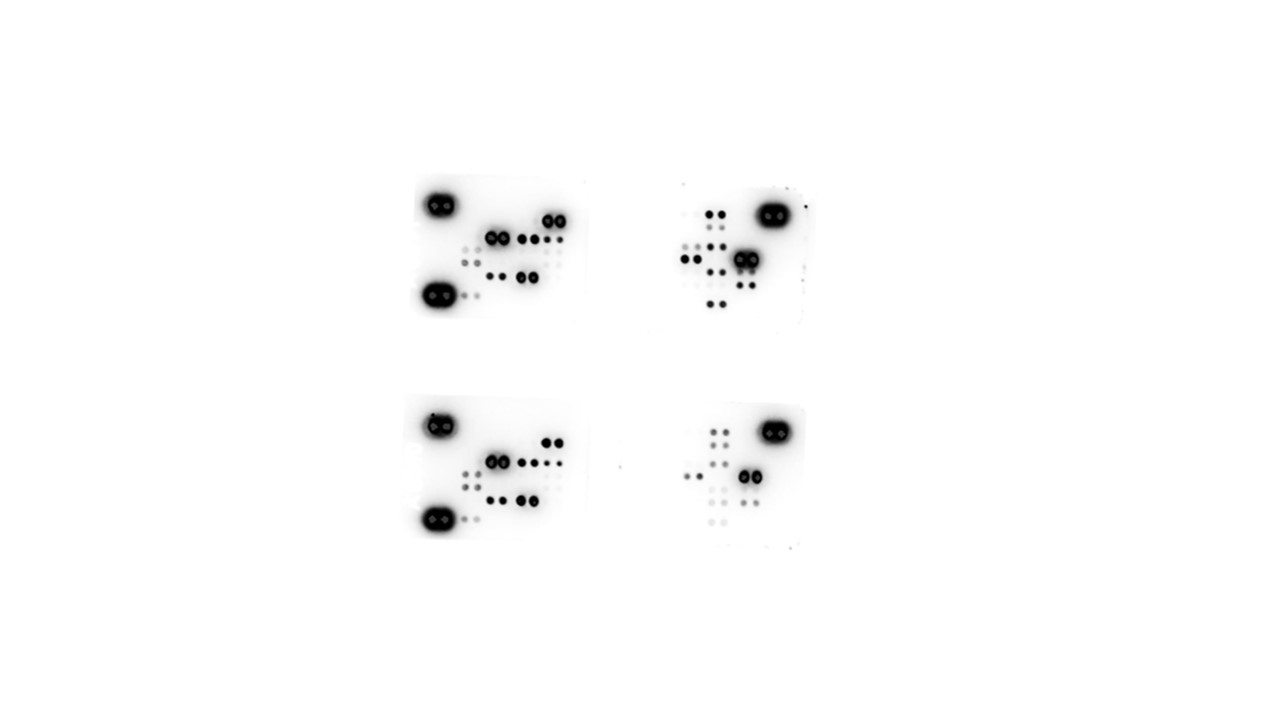

Supplement: Figure 7—source data 1. [file elife-84238-fig7-data1.zip › z Figure 7-Source Data 1/Figure 7-Source Data 1/original files/7A/rep1.jpg]

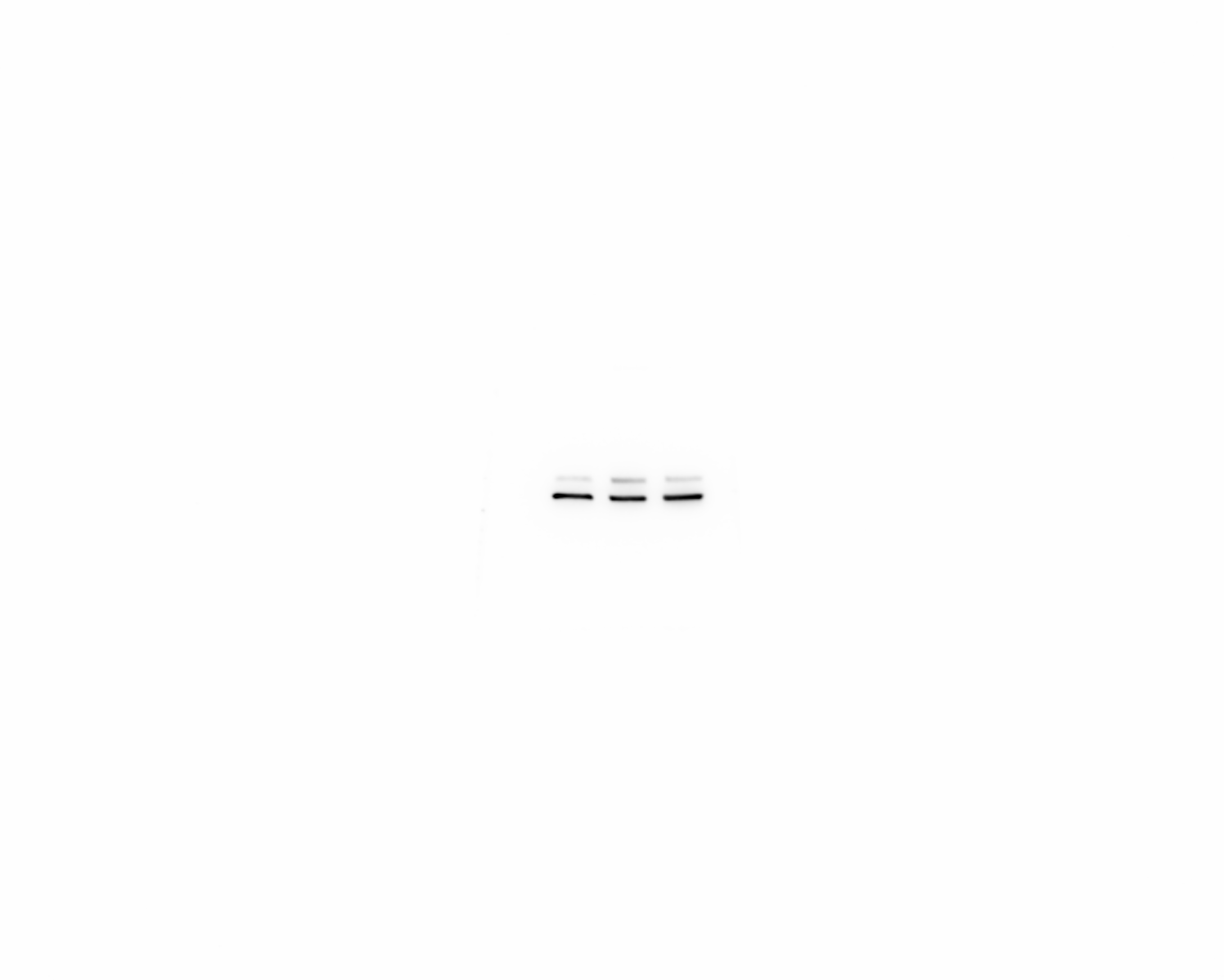

Supplement: Figure 7—source data 1. [file elife-84238-fig7-data1.zip › z Figure 7-Source Data 1/Figure 7-Source Data 1/original files/7B/IMR-90/ACTB.jpg]

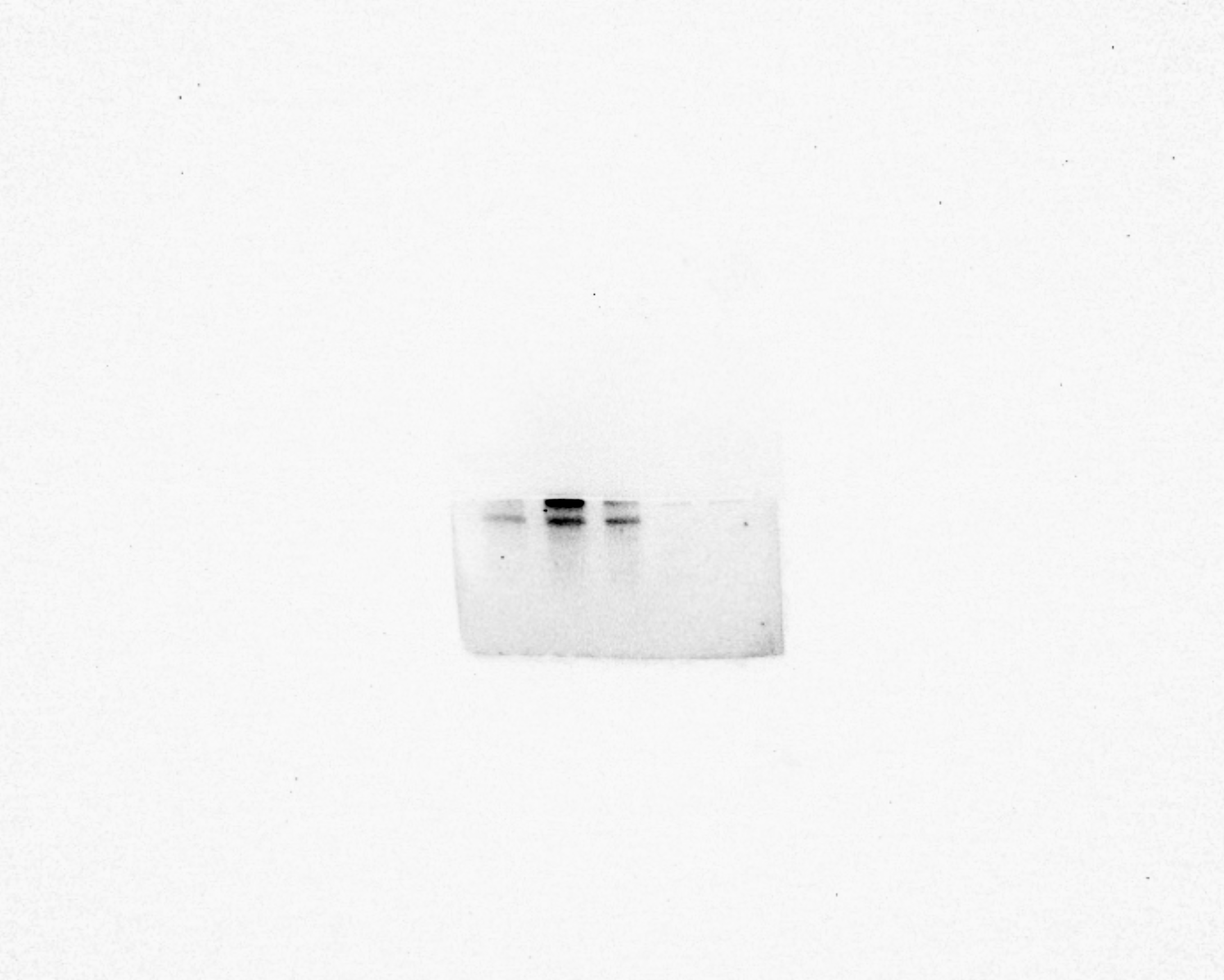

Supplement: Figure 7—source data 1. [file elife-84238-fig7-data1.zip › z Figure 7-Source Data 1/Figure 7-Source Data 1/original files/7B/IMR-90/BAFF.jpg]

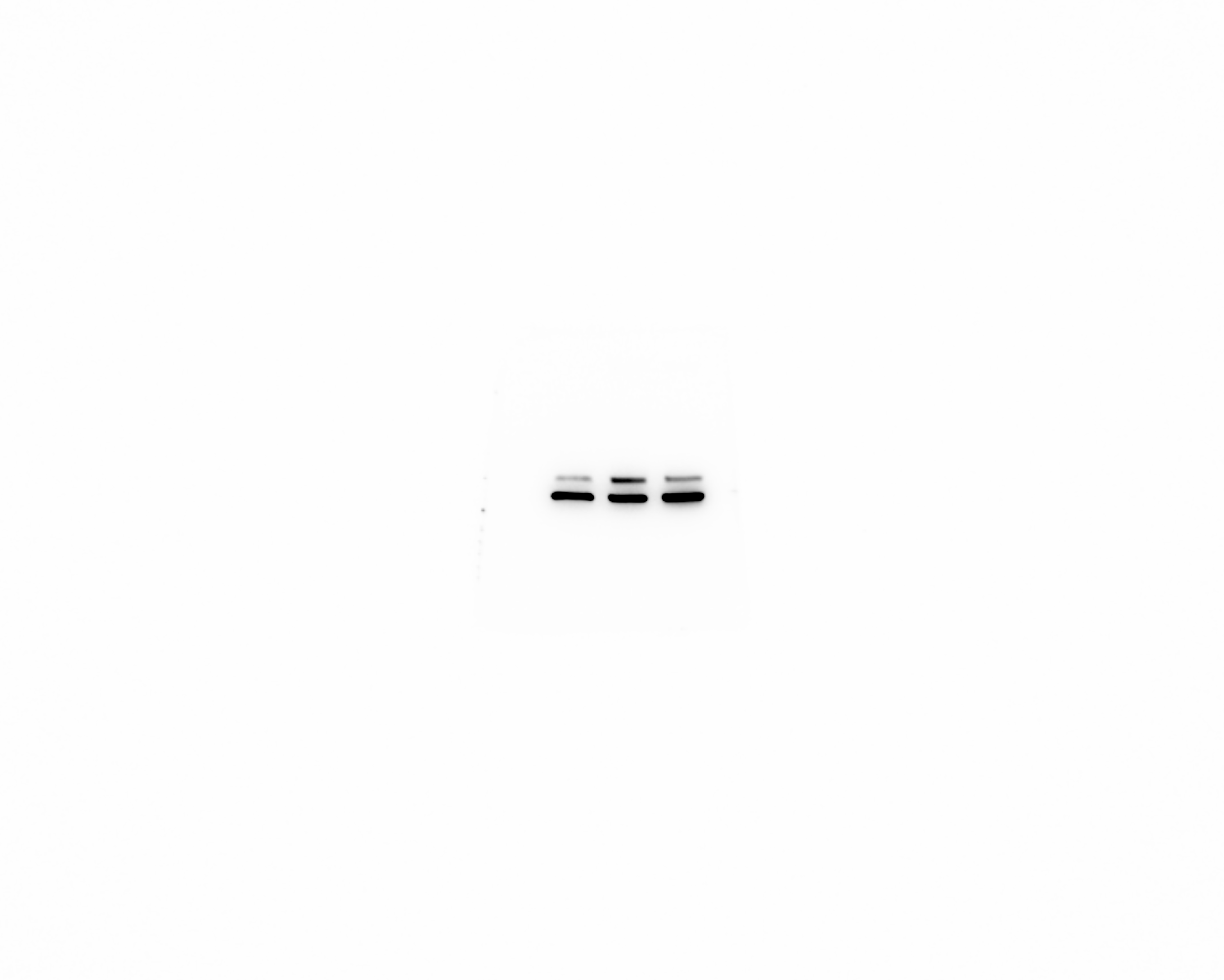

Supplement: Figure 7—source data 1. [file elife-84238-fig7-data1.zip › z Figure 7-Source Data 1/Figure 7-Source Data 1/original files/7B/IMR-90/p53.jpg]

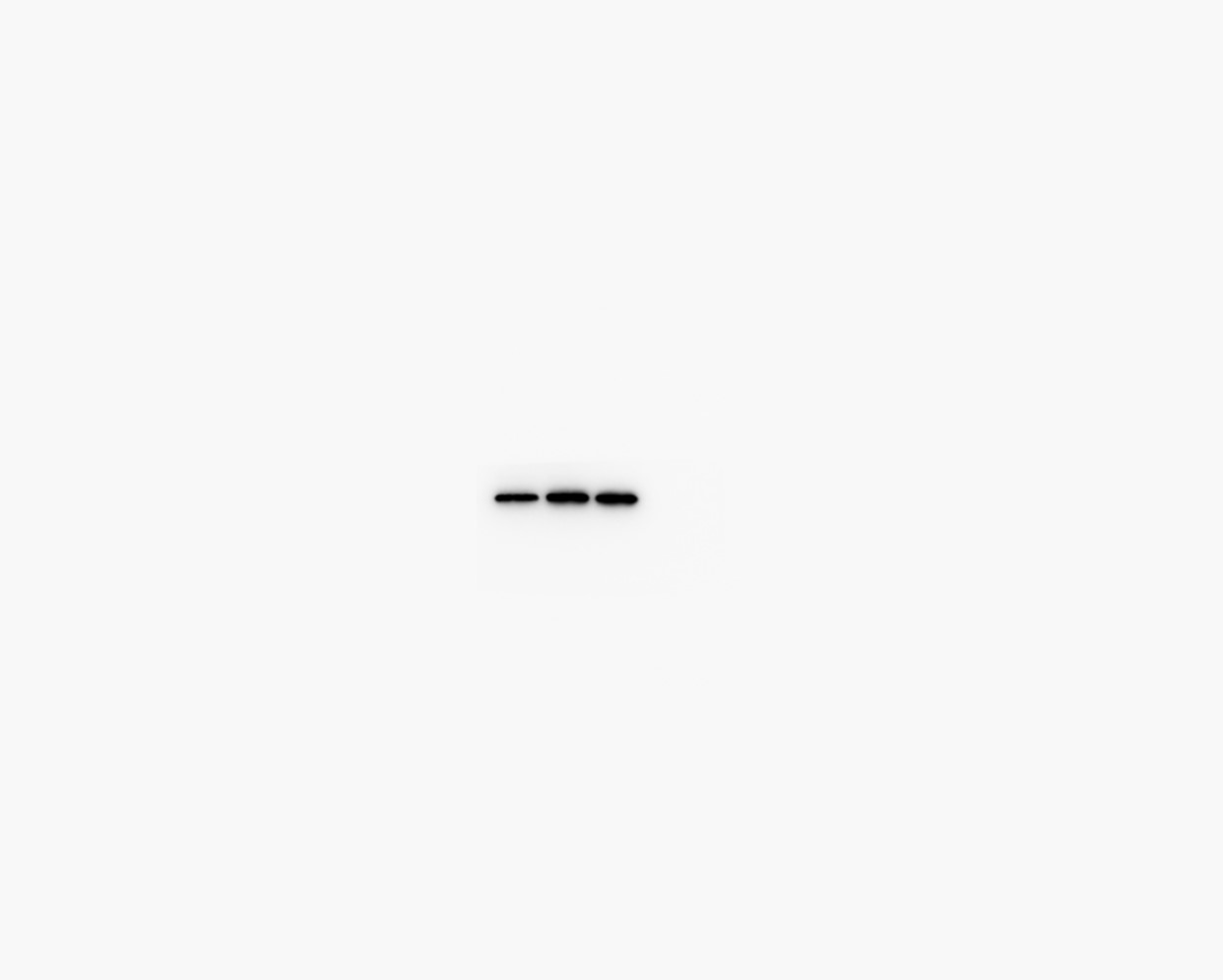

Supplement: Figure 7—source data 1. [file elife-84238-fig7-data1.zip › z Figure 7-Source Data 1/Figure 7-Source Data 1/original files/7B/RAW 264.7/ACTB.jpg]

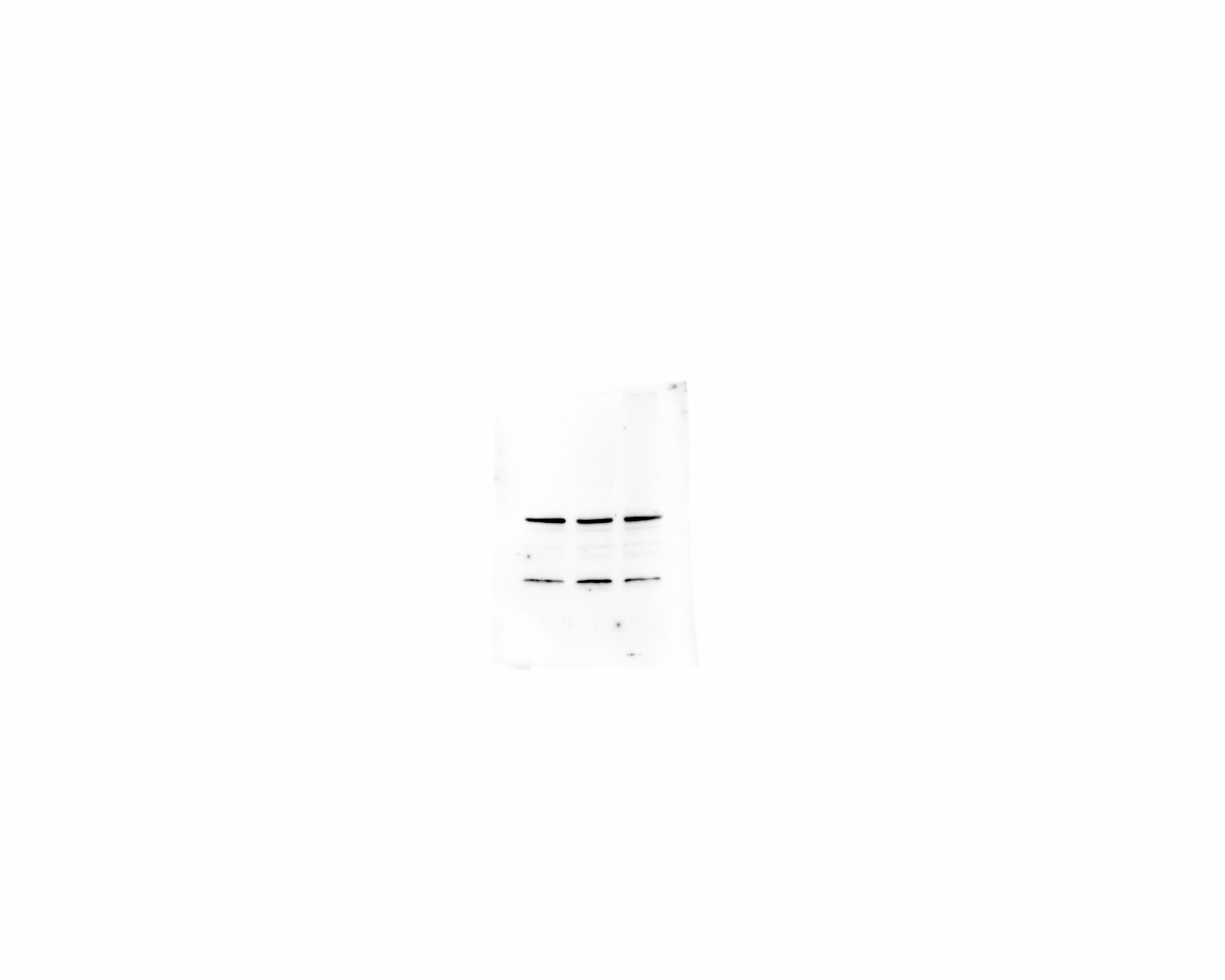

Supplement: Figure 7—source data 1. [file elife-84238-fig7-data1.zip › z Figure 7-Source Data 1/Figure 7-Source Data 1/original files/7B/RAW 264.7/BAFF.jpg]

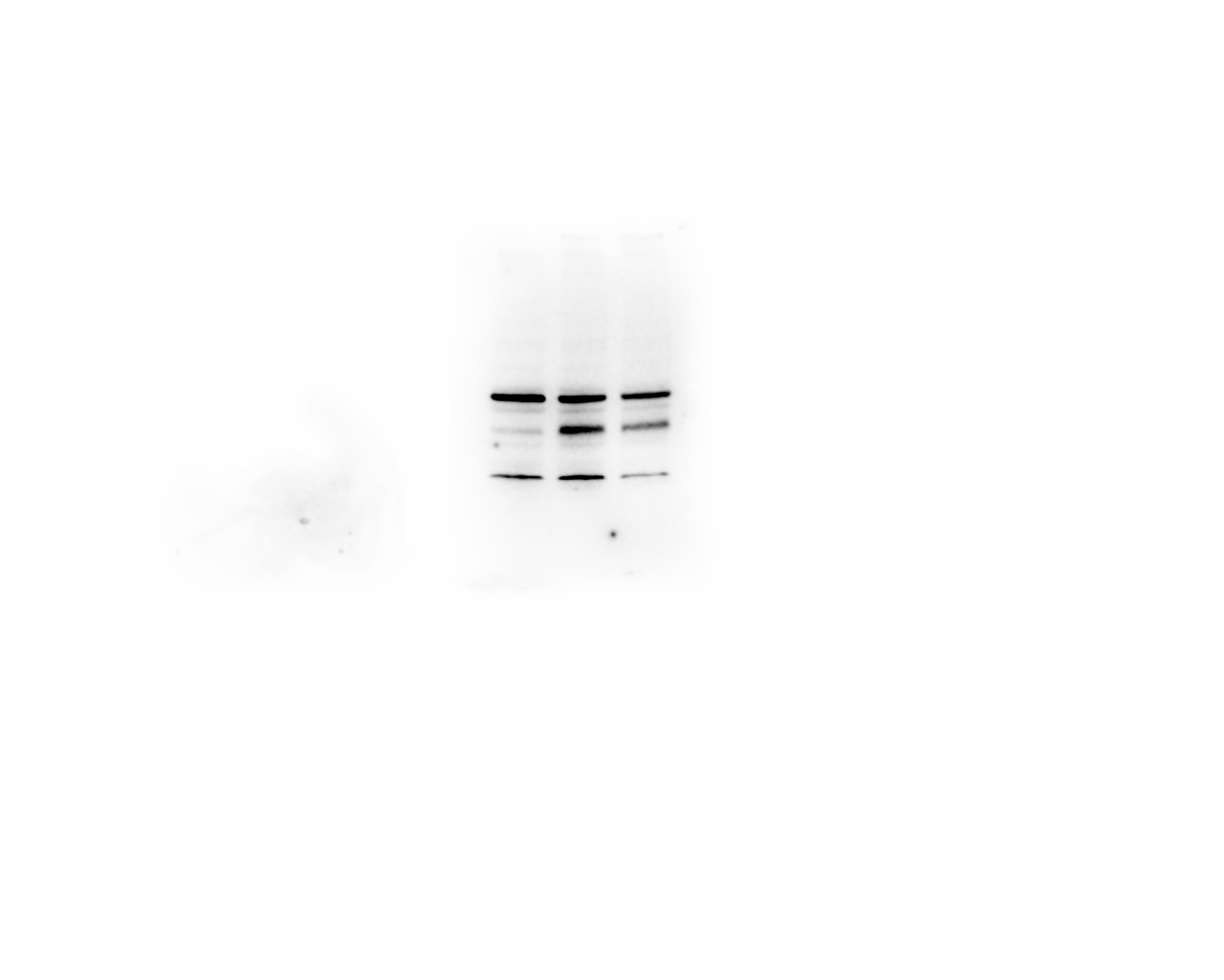

Supplement: Figure 7—source data 1. [file elife-84238-fig7-data1.zip › z Figure 7-Source Data 1/Figure 7-Source Data 1/original files/7B/RAW 264.7/p53.jpg]

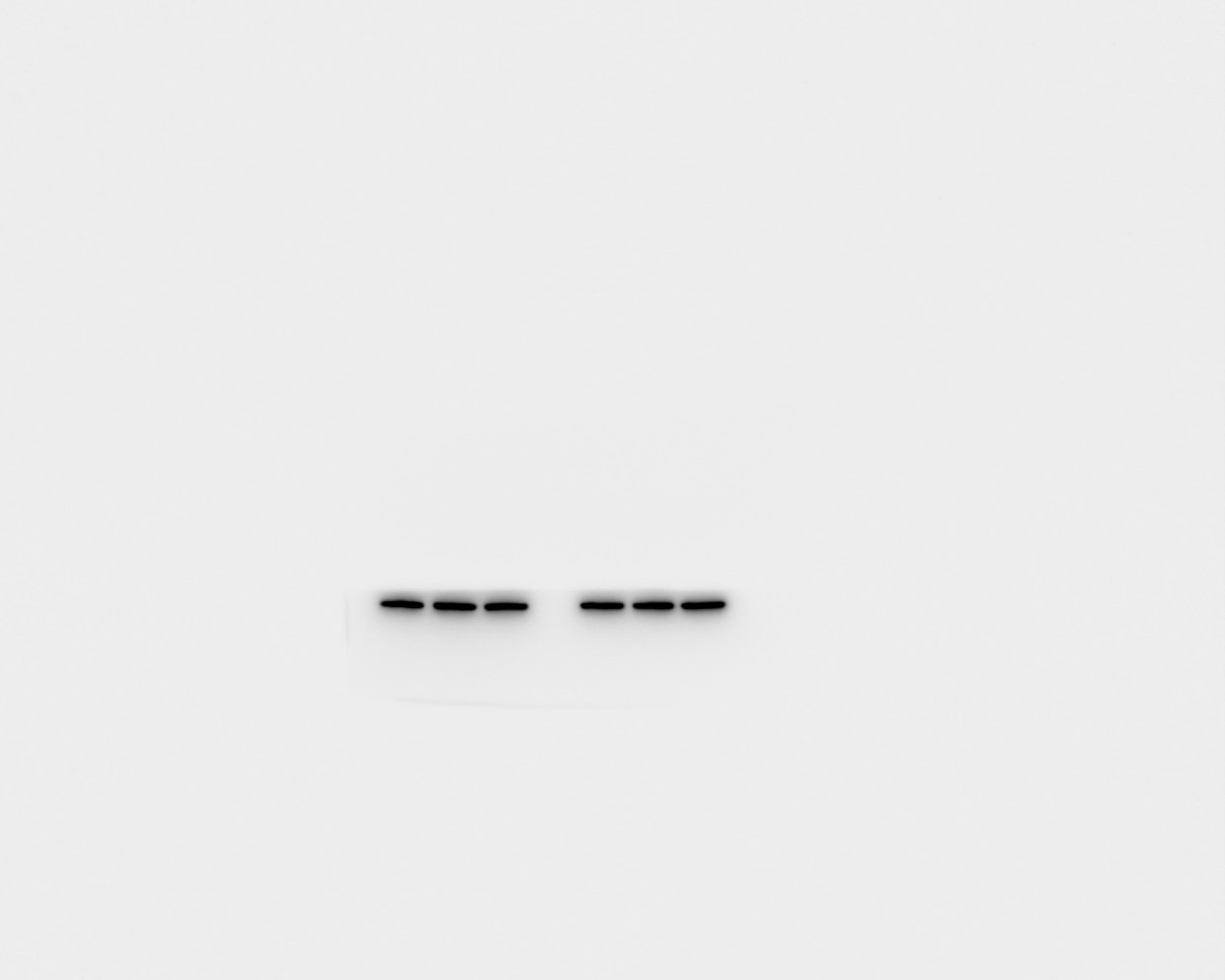

Supplement: Figure 7—source data 1. [file elife-84238-fig7-data1.zip › z Figure 7-Source Data 1/Figure 7-Source Data 1/original files/7B/WI-38 and THP-1/ACTB.jpg]

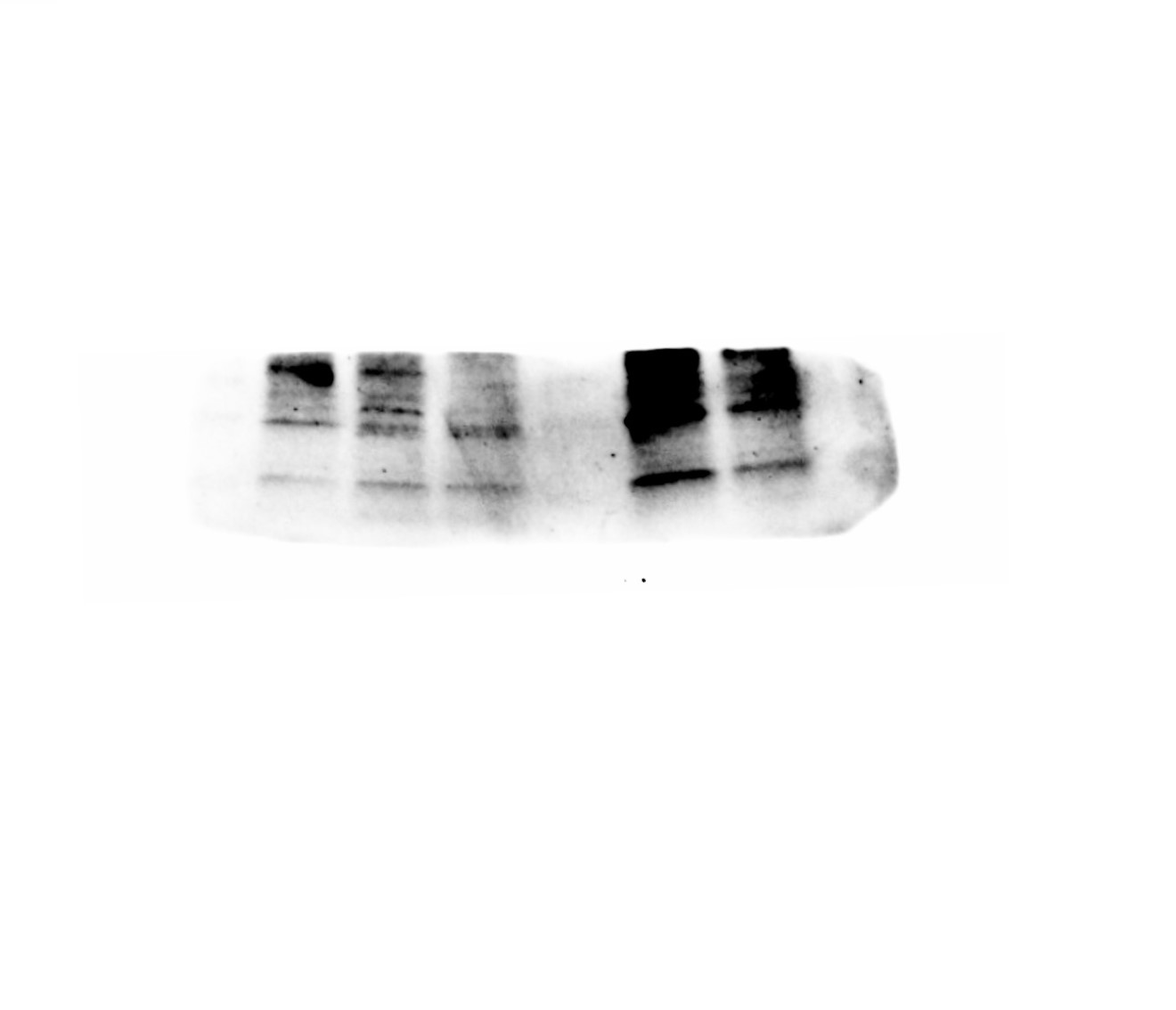

Supplement: Figure 7—source data 1. [file elife-84238-fig7-data1.zip › z Figure 7-Source Data 1/Figure 7-Source Data 1/original files/7B/WI-38 and THP-1/BAFF_WI38_30ug.jpg]

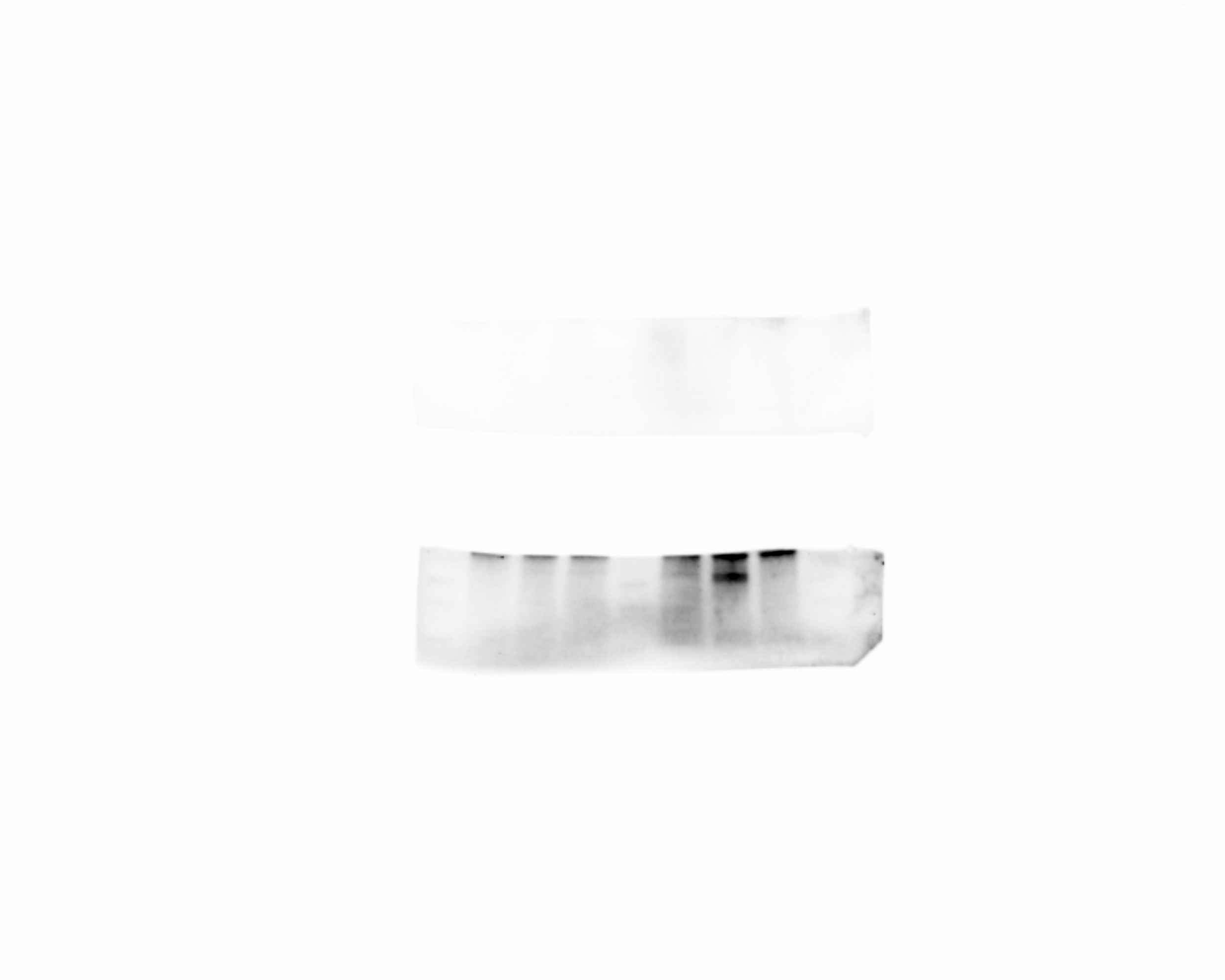

Supplement: Figure 7—source data 1. [file elife-84238-fig7-data1.zip › z Figure 7-Source Data 1/Figure 7-Source Data 1/original files/7B/WI-38 and THP-1/BAFF10ug.jpg]

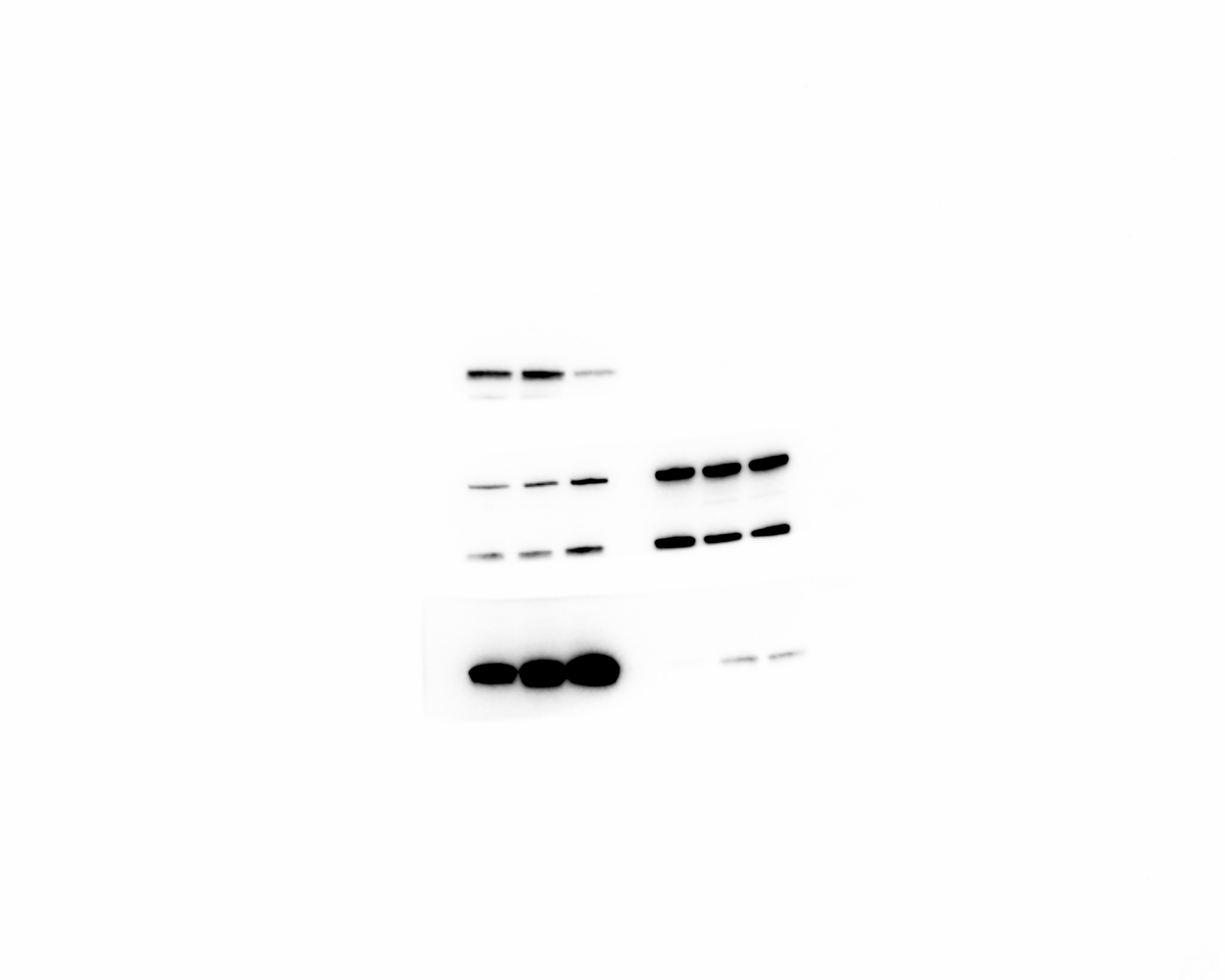

Supplement: Figure 7—source data 1. [file elife-84238-fig7-data1.zip › z Figure 7-Source Data 1/Figure 7-Source Data 1/original files/7B/WI-38 and THP-1/p53.jpg]

A

WI-38 Phospho-kinase array

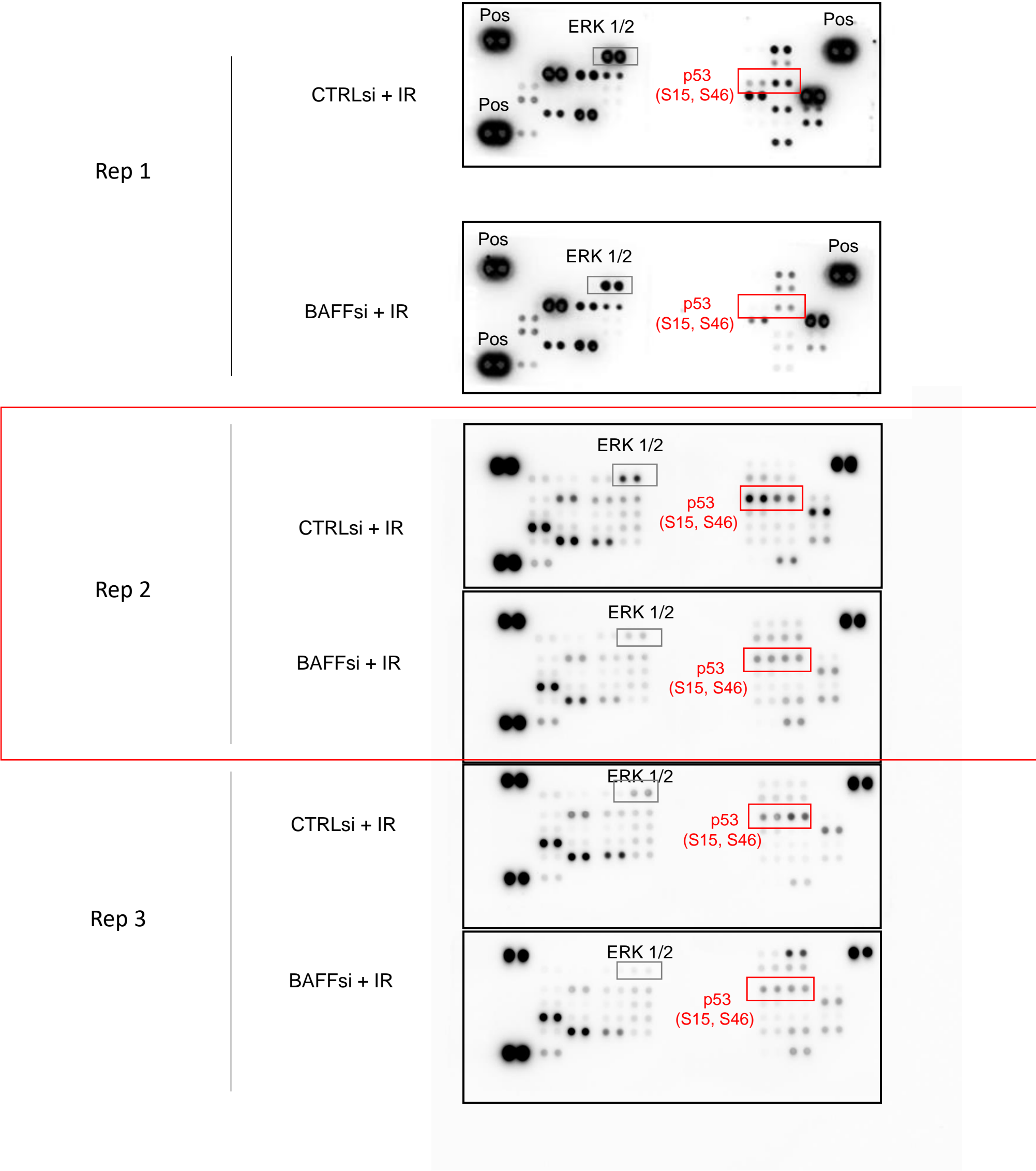

B

IMR-90

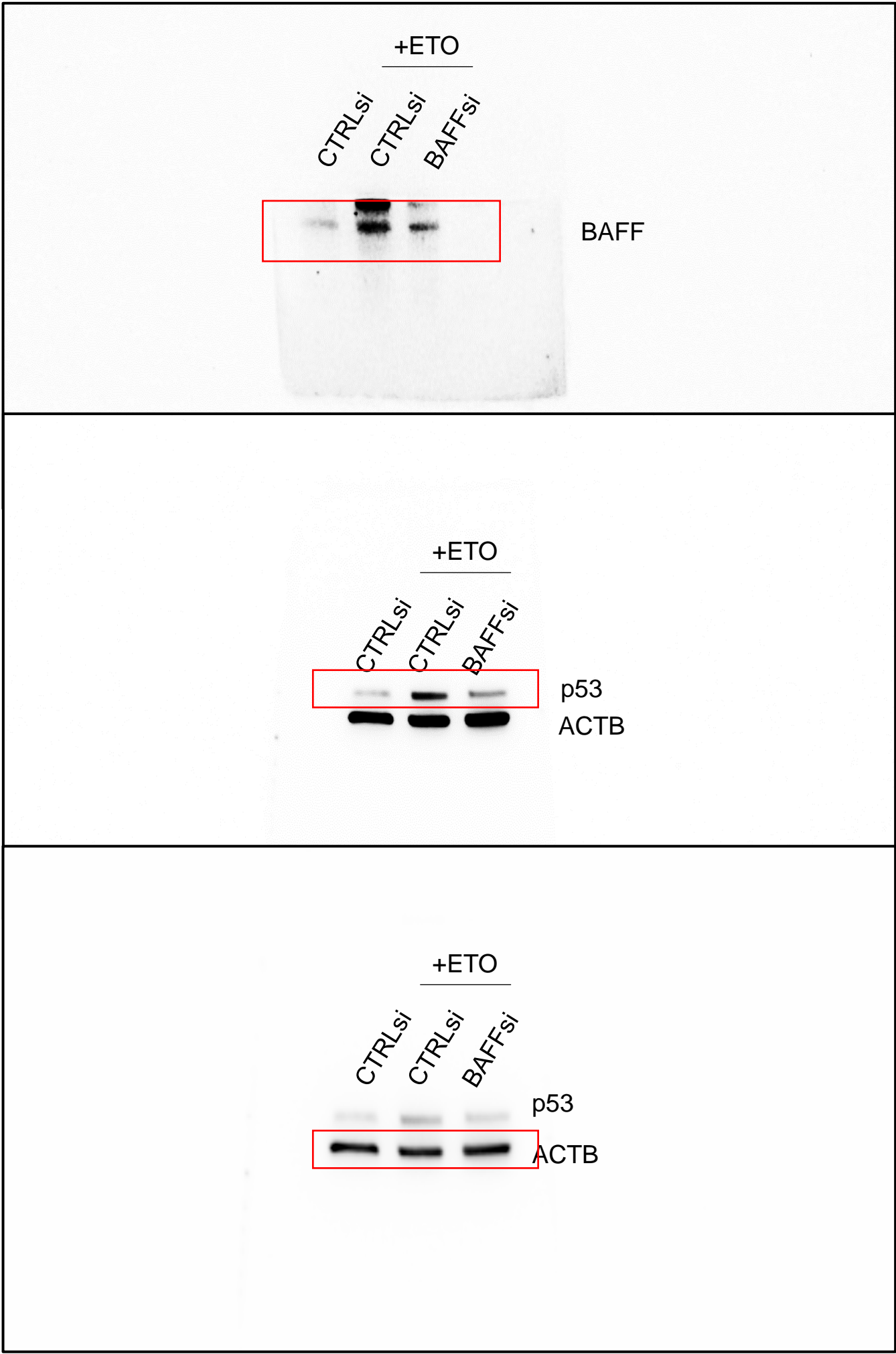

B,C

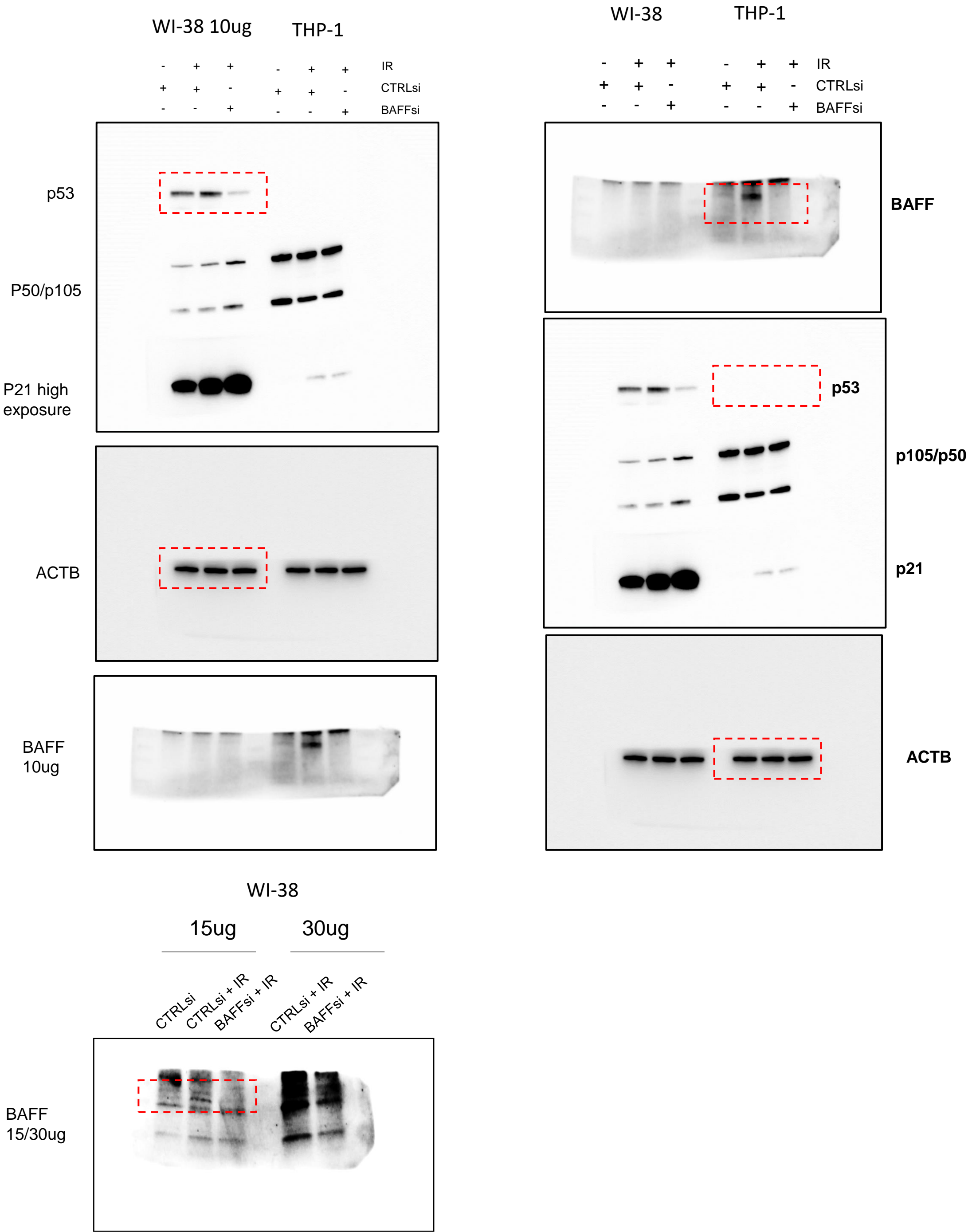

C

RAW 264.7

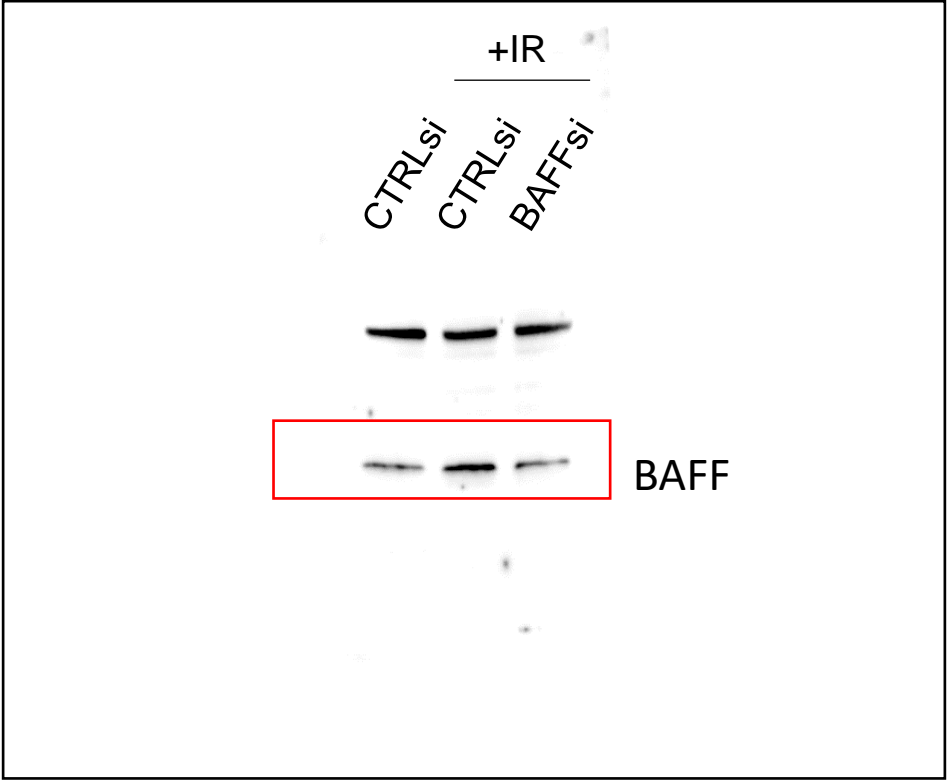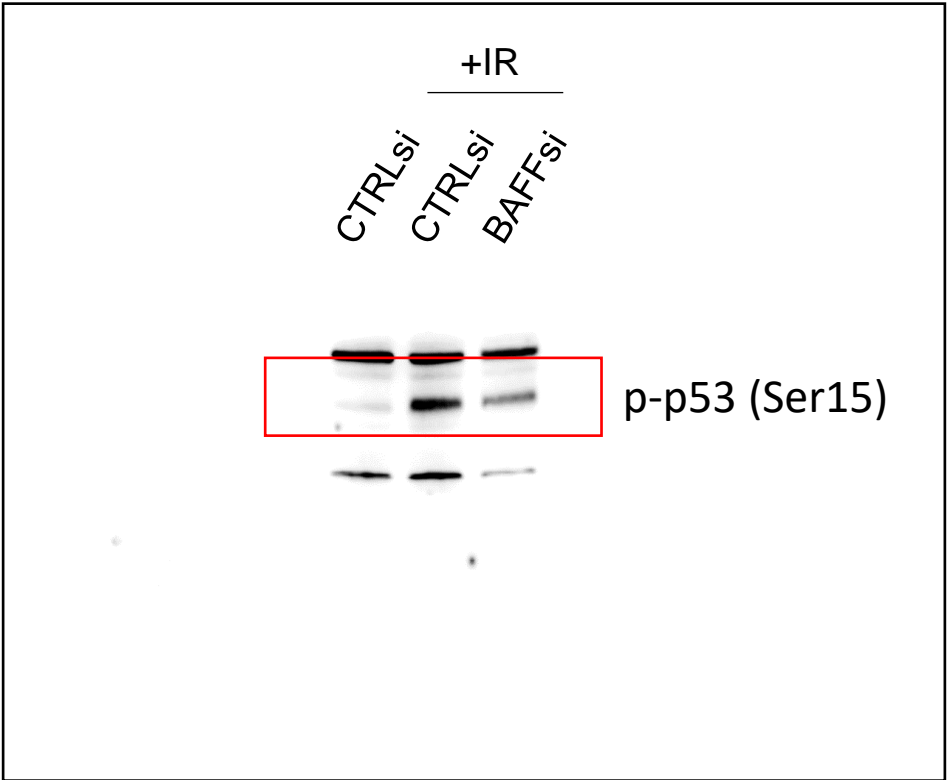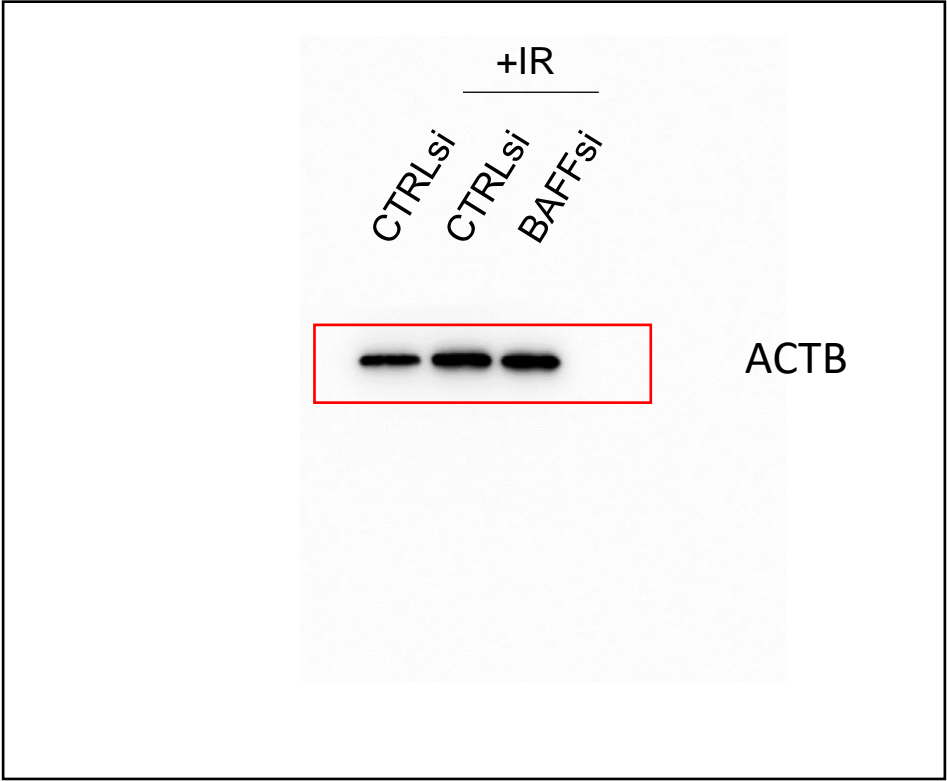

Supplement: Figure 7—source data 1. [file elife-84238-fig7-data1.zip › z Figure 7-Source Data 1/Figure 7-Source Data 1/uncropped blots Fig 7.pdf]

A

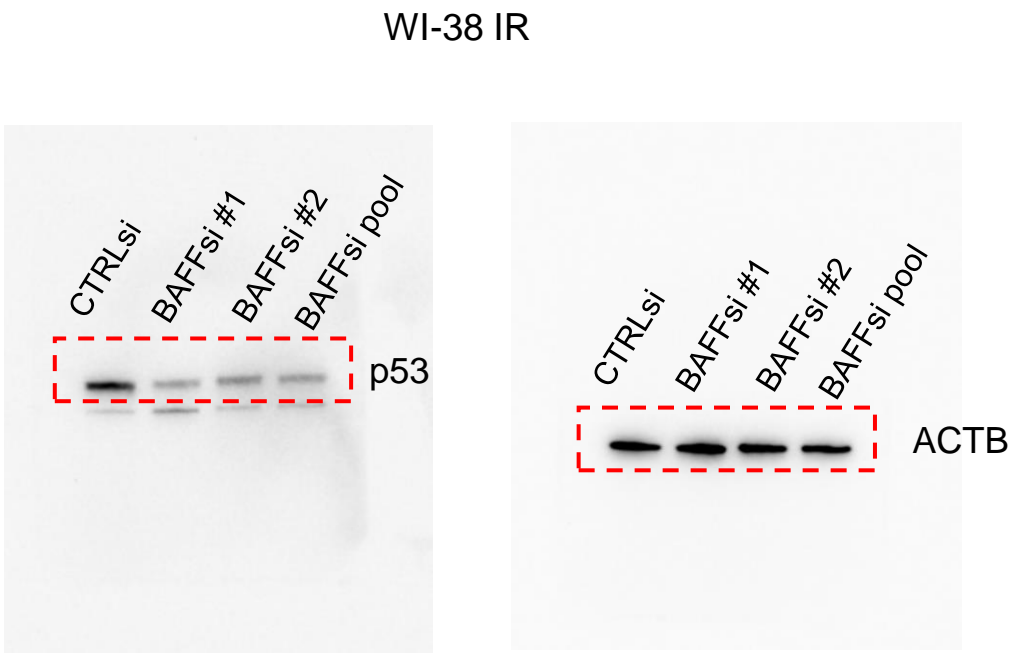

B

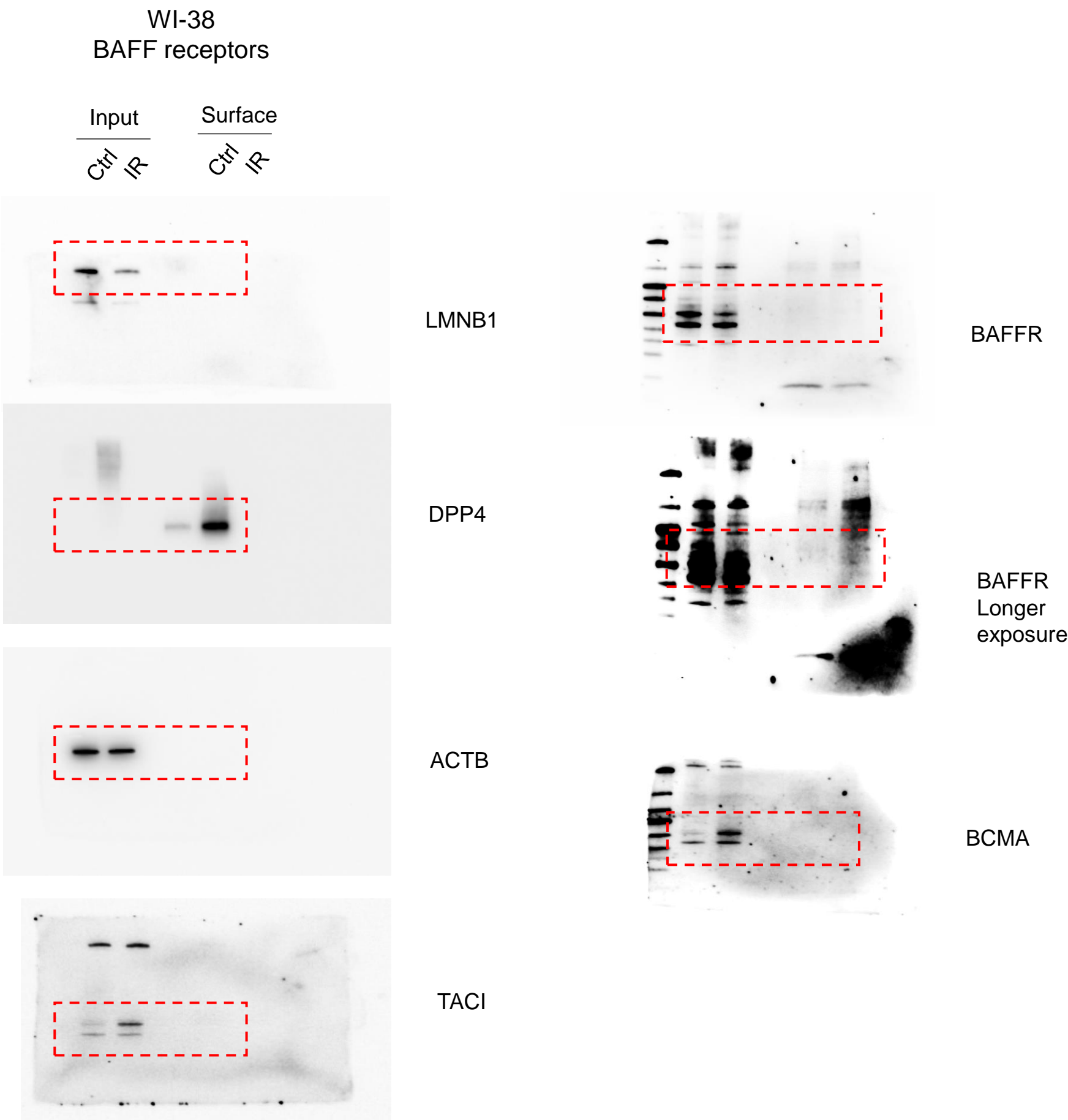

C

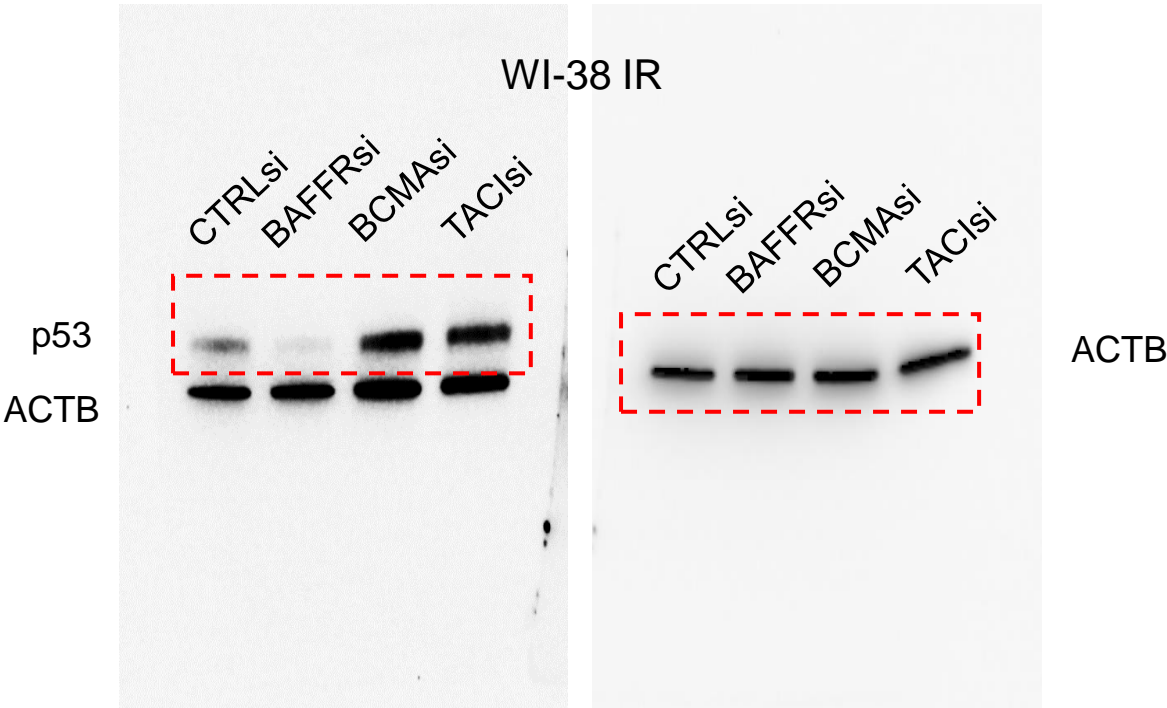

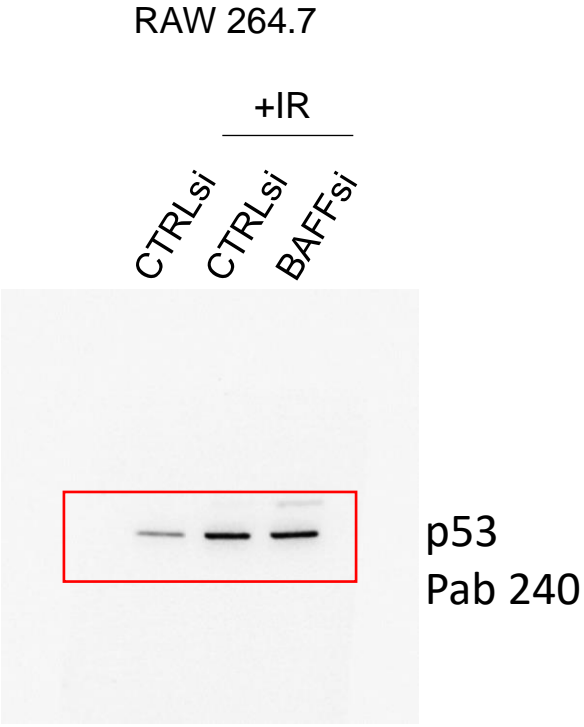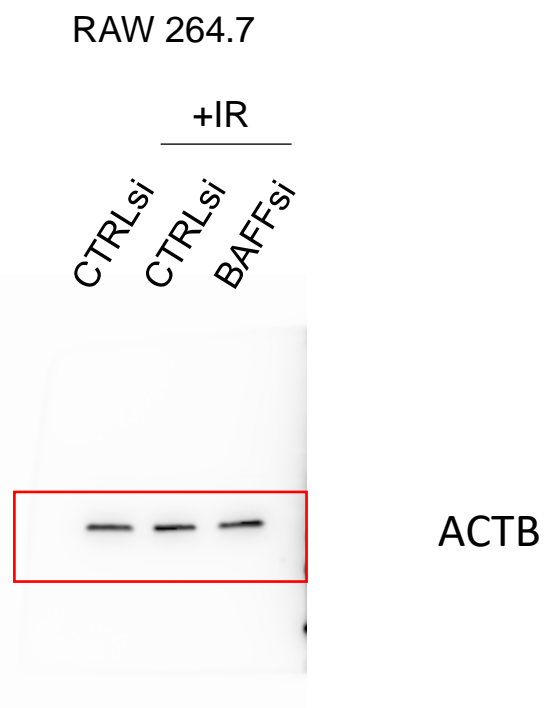

Supplement: Figure 7—figure supplement 1—source data 1. [file elife-84238-fig7-figsupp1-data1.zip › z Figure 7-Figure supplement 1- Source Data 1/Figure 7-Figure supplement 1- Source Data 1/blots figure S7 .pdf]

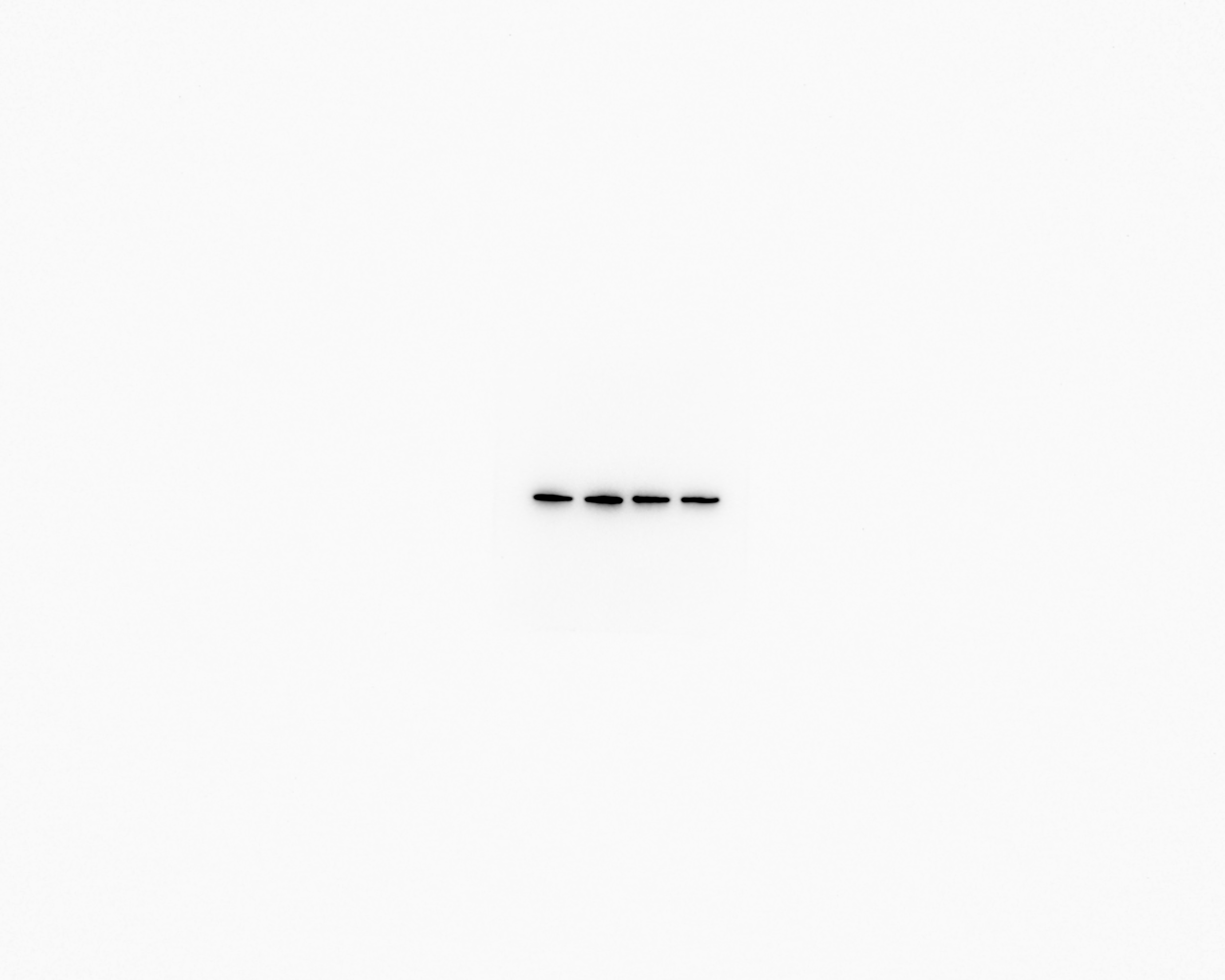

Supplement: Figure 7—figure supplement 1—source data 1. [file elife-84238-fig7-figsupp1-data1.zip › z Figure 7-Figure supplement 1- Source Data 1/Figure 7-Figure supplement 1- Source Data 1/original pics/A/ACTB.jpg]

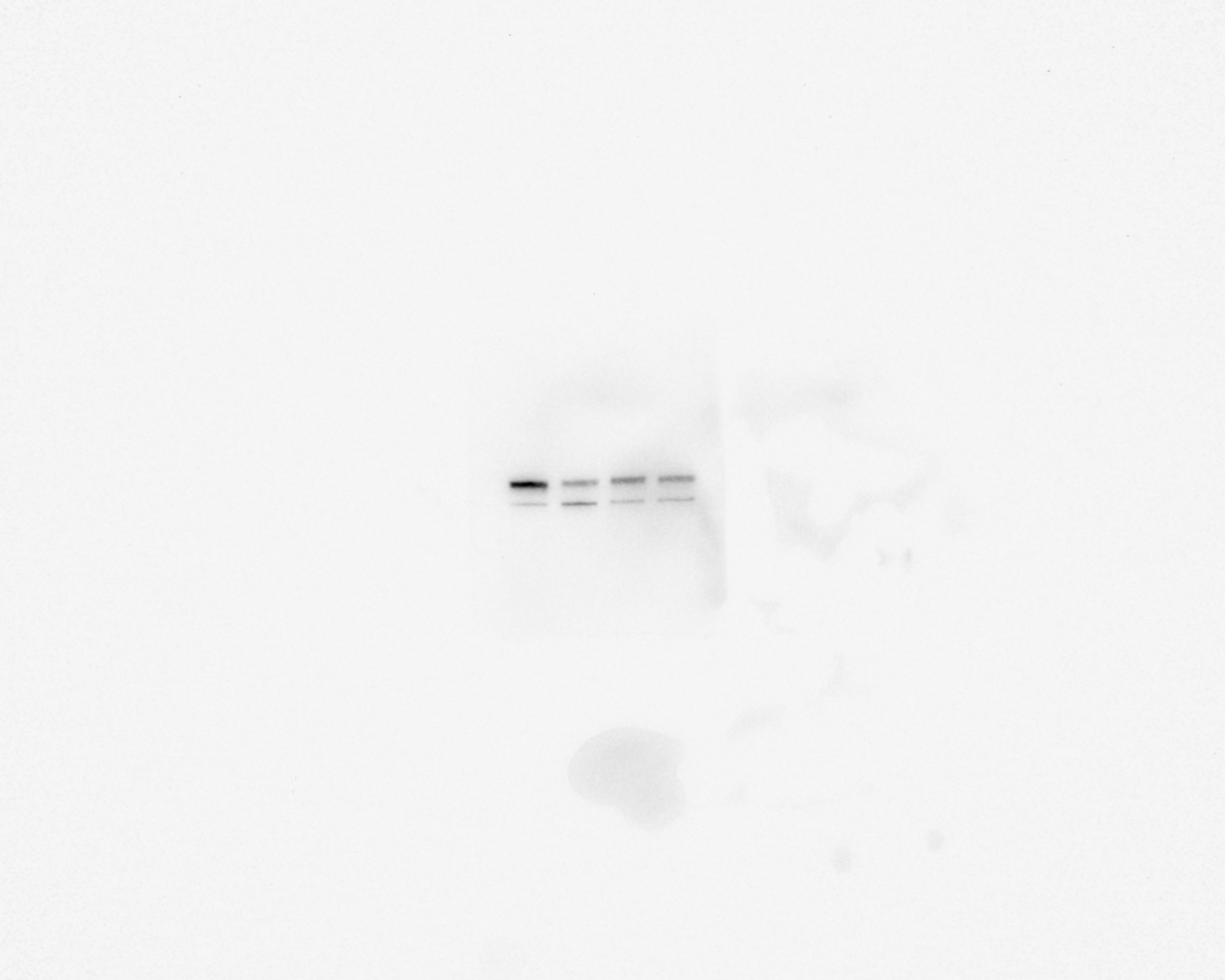

Supplement: Figure 7—figure supplement 1—source data 1. [file elife-84238-fig7-figsupp1-data1.zip › z Figure 7-Figure supplement 1- Source Data 1/Figure 7-Figure supplement 1- Source Data 1/original pics/A/p53.jpg]

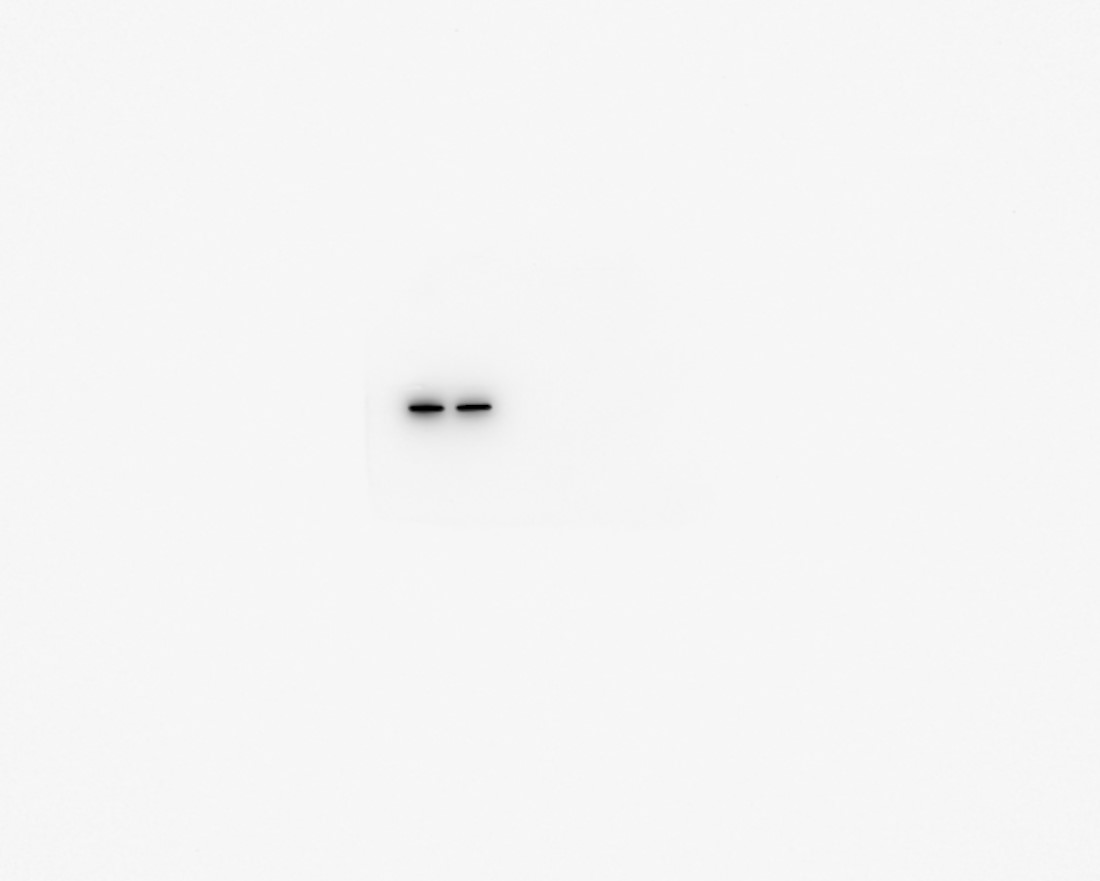

Supplement: Figure 7—figure supplement 1—source data 1. [file elife-84238-fig7-figsupp1-data1.zip › z Figure 7-Figure supplement 1- Source Data 1/Figure 7-Figure supplement 1- Source Data 1/original pics/B/ACTB.jpg]

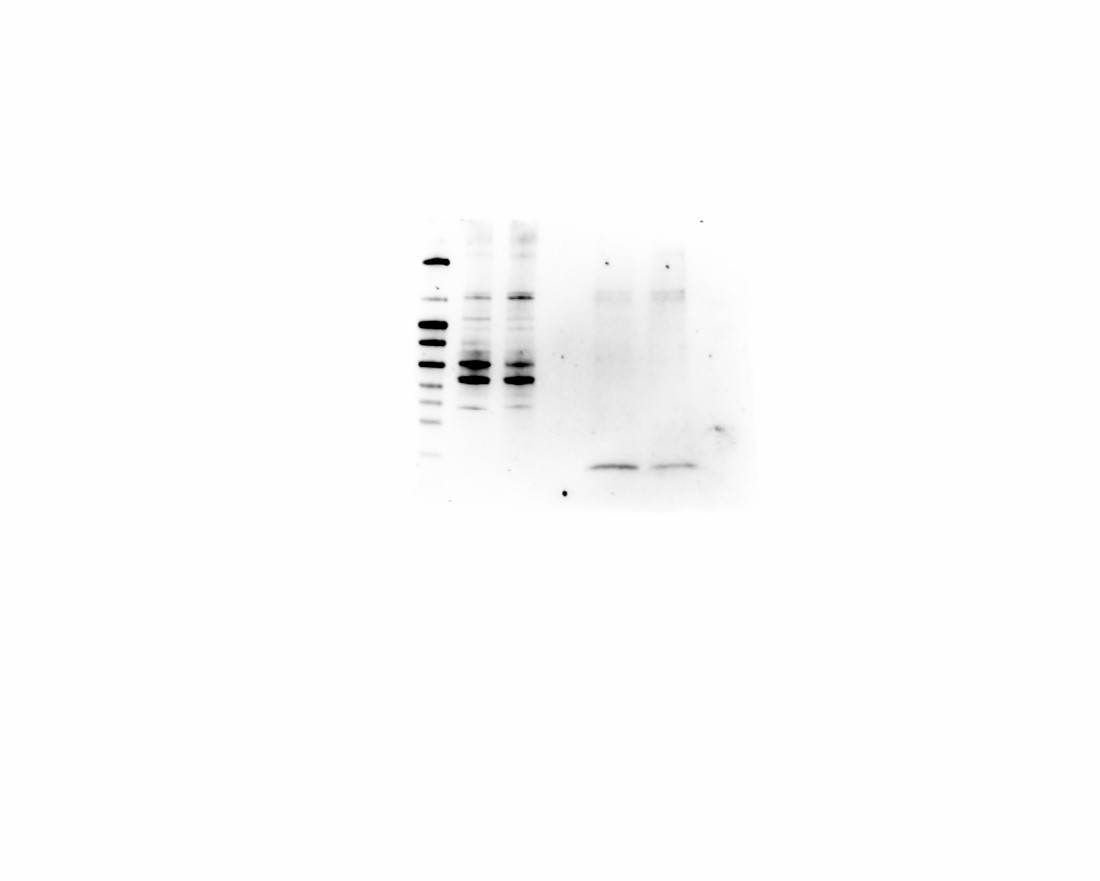

Supplement: Figure 7—figure supplement 1—source data 1. [file elife-84238-fig7-figsupp1-data1.zip › z Figure 7-Figure supplement 1- Source Data 1/Figure 7-Figure supplement 1- Source Data 1/original pics/B/BAFFR1.jpg]

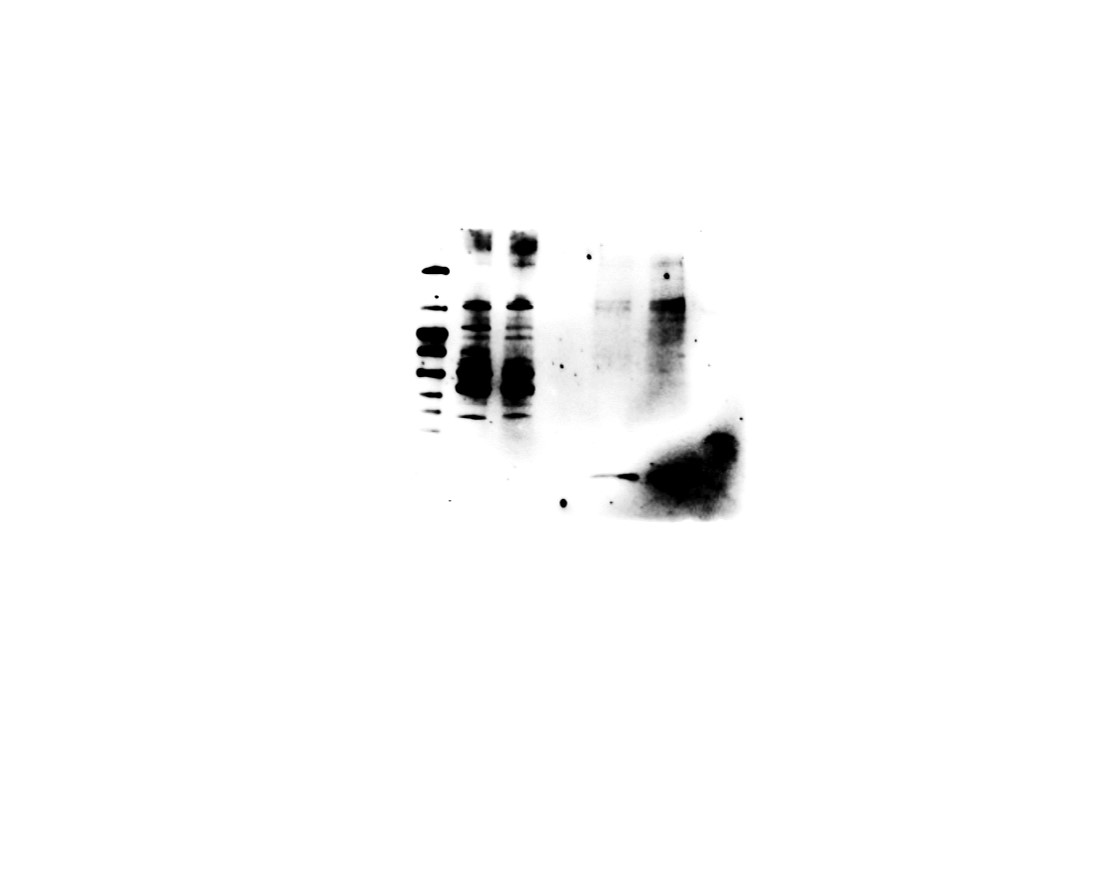

Supplement: Figure 7—figure supplement 1—source data 1. [file elife-84238-fig7-figsupp1-data1.zip › z Figure 7-Figure supplement 1- Source Data 1/Figure 7-Figure supplement 1- Source Data 1/original pics/B/BAFFR2.jpg]

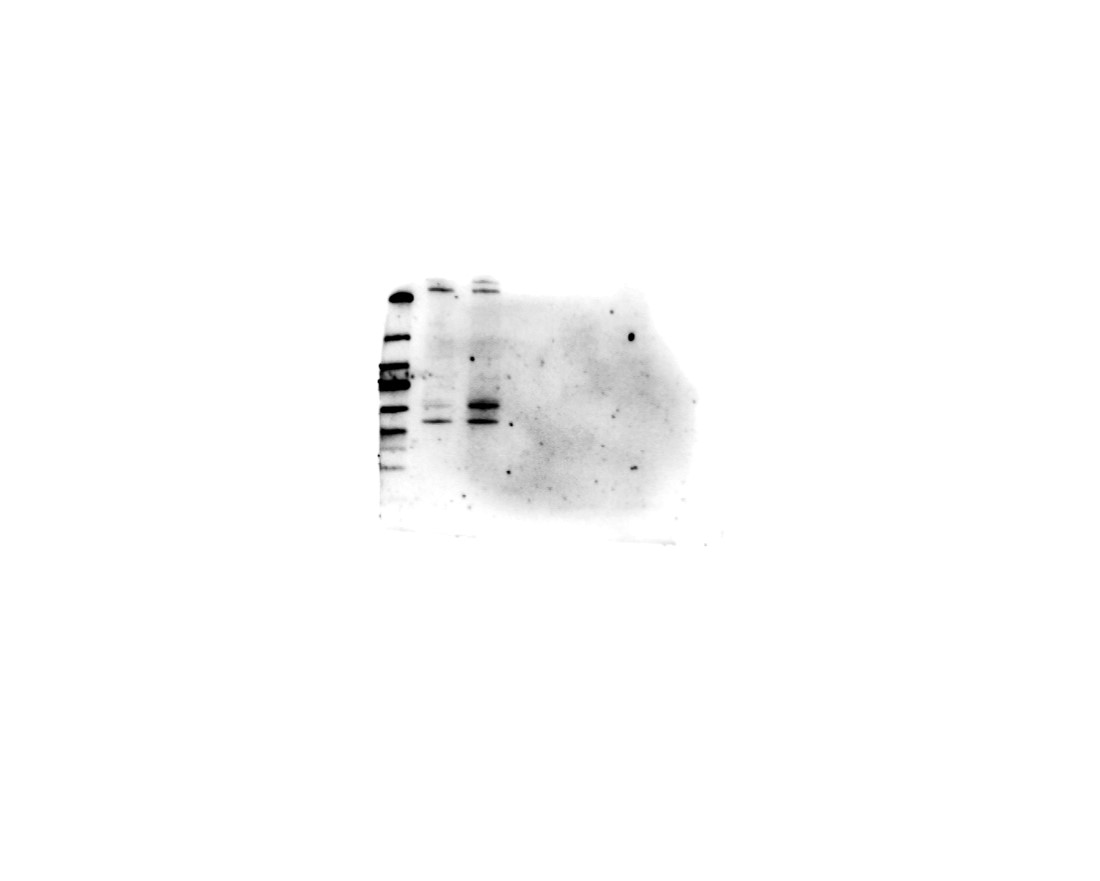

Supplement: Figure 7—figure supplement 1—source data 1. [file elife-84238-fig7-figsupp1-data1.zip › z Figure 7-Figure supplement 1- Source Data 1/Figure 7-Figure supplement 1- Source Data 1/original pics/B/BCMA.jpg]

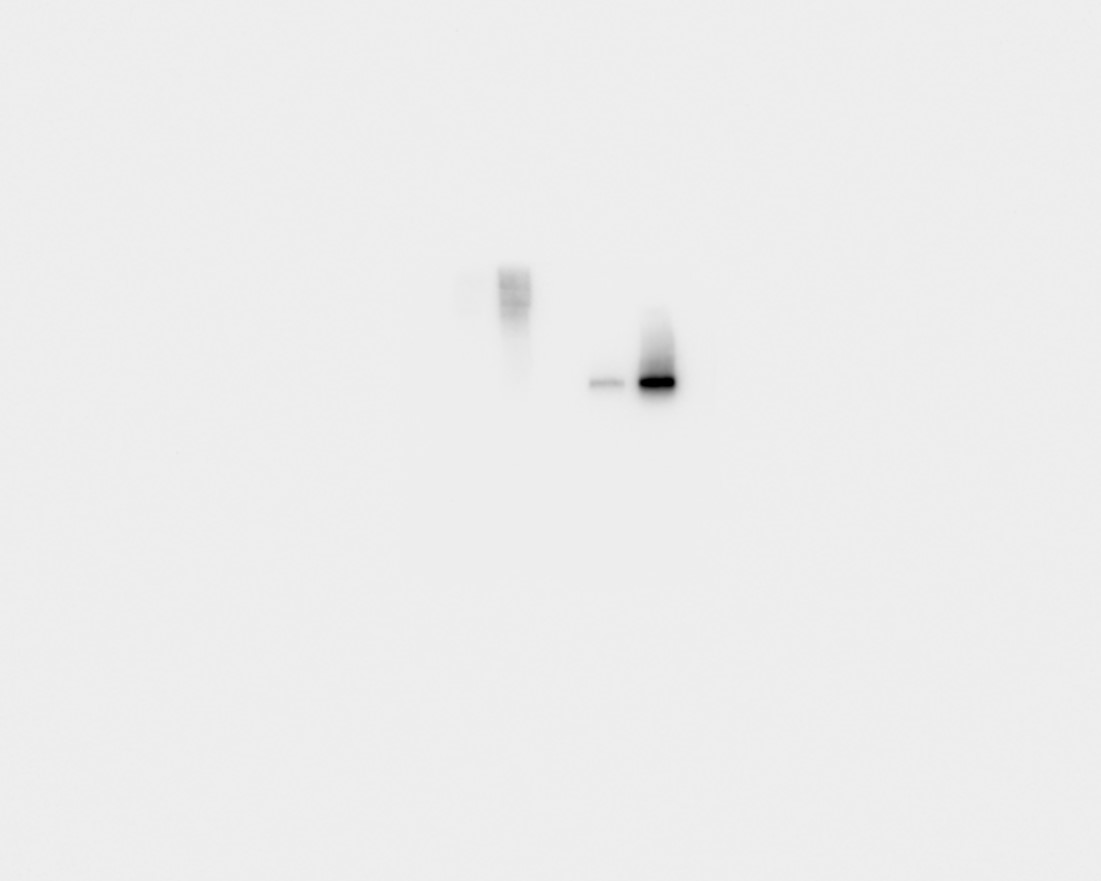

Supplement: Figure 7—figure supplement 1—source data 1. [file elife-84238-fig7-figsupp1-data1.zip › z Figure 7-Figure supplement 1- Source Data 1/Figure 7-Figure supplement 1- Source Data 1/original pics/B/DPP4.jpg]

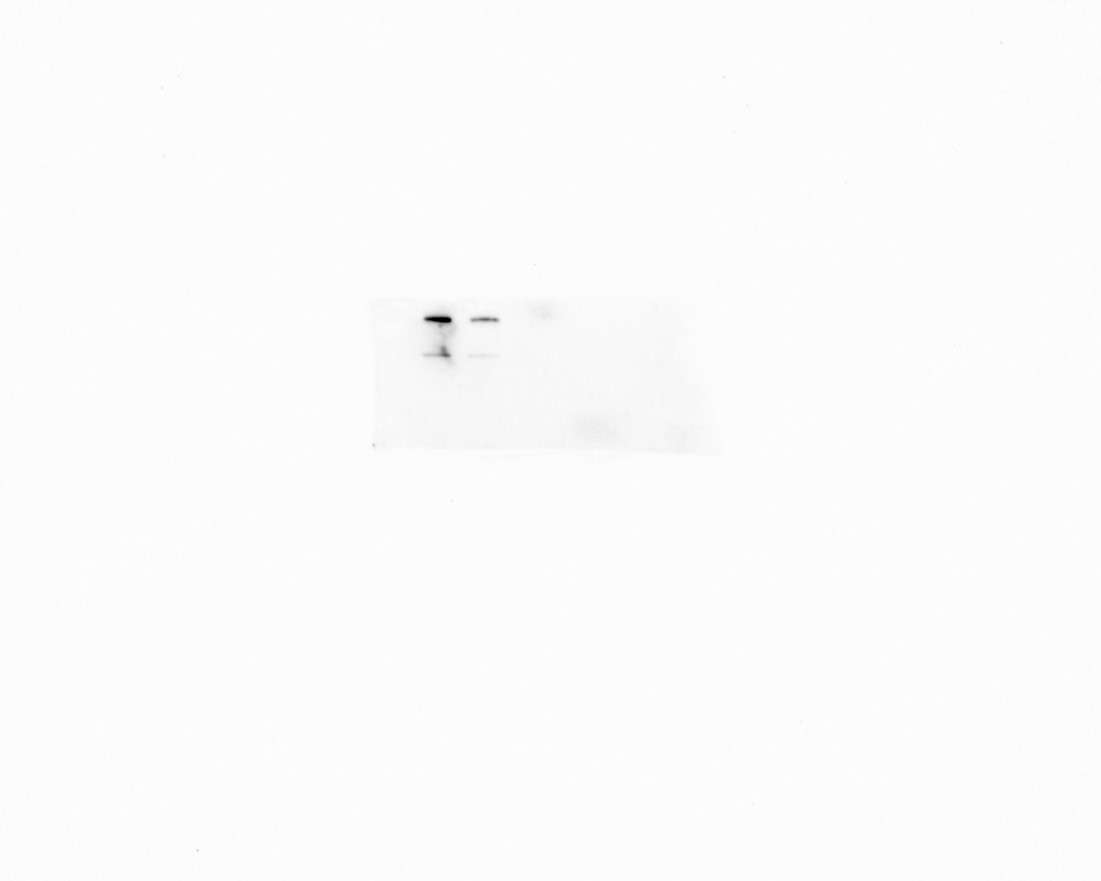

Supplement: Figure 7—figure supplement 1—source data 1. [file elife-84238-fig7-figsupp1-data1.zip › z Figure 7-Figure supplement 1- Source Data 1/Figure 7-Figure supplement 1- Source Data 1/original pics/B/LMNB1.jpg]

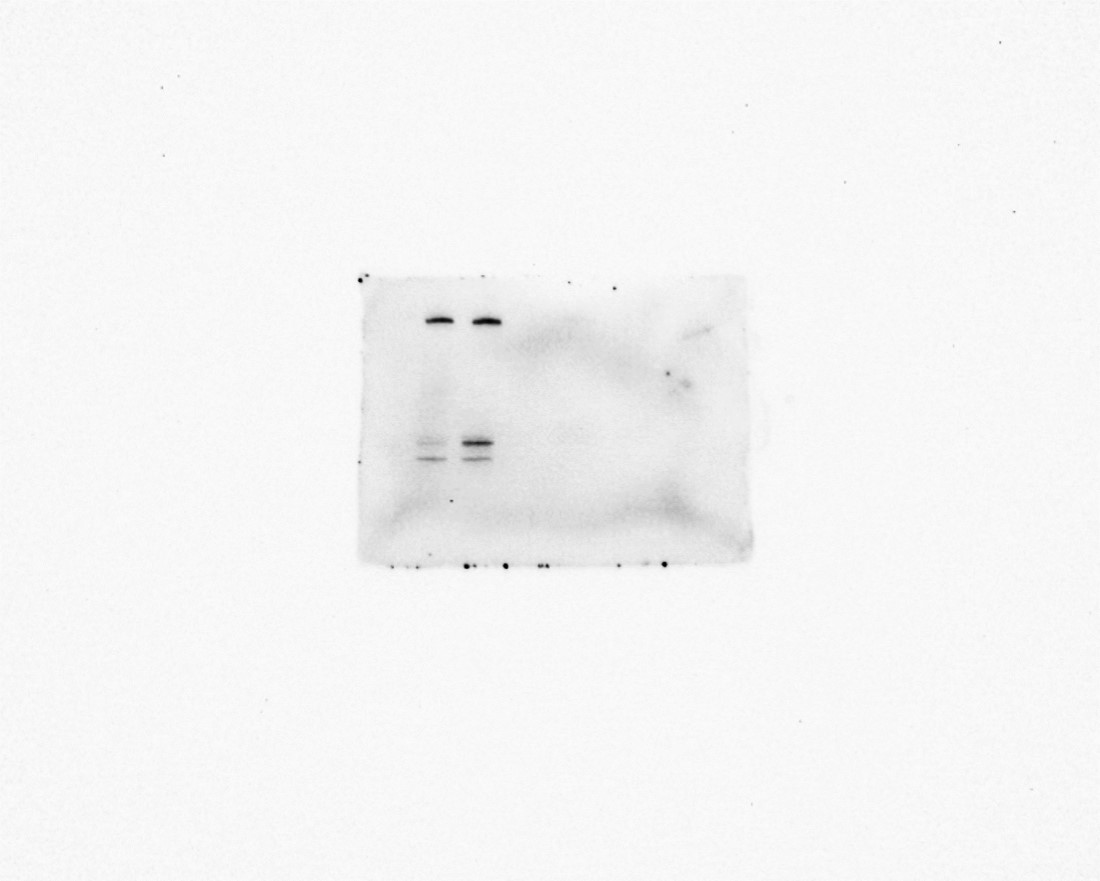

Supplement: Figure 7—figure supplement 1—source data 1. [file elife-84238-fig7-figsupp1-data1.zip › z Figure 7-Figure supplement 1- Source Data 1/Figure 7-Figure supplement 1- Source Data 1/original pics/B/TACI.jpg]

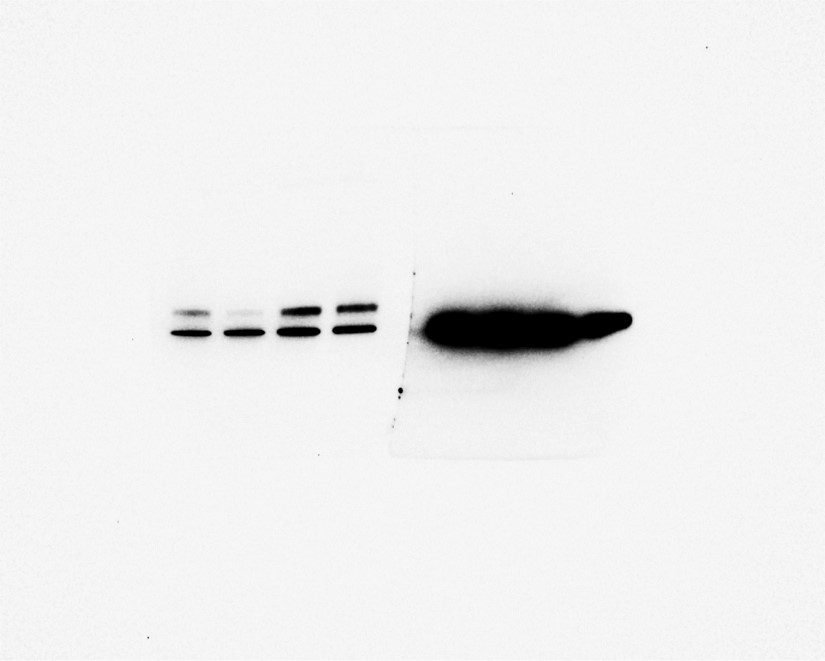

Supplement: Figure 7—figure supplement 1—source data 1. [file elife-84238-fig7-figsupp1-data1.zip › z Figure 7-Figure supplement 1- Source Data 1/Figure 7-Figure supplement 1- Source Data 1/original pics/C/ACTB and p53.jpg]

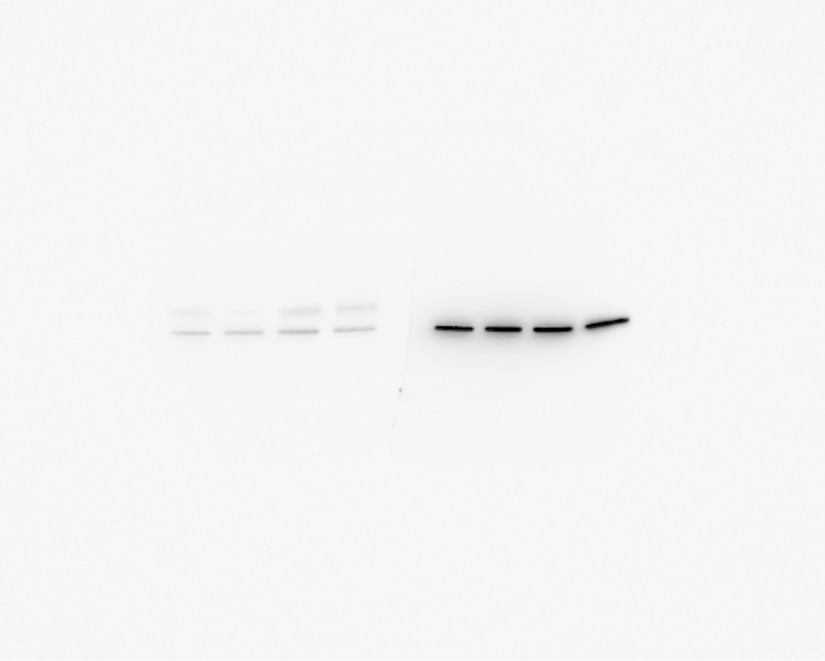

Supplement: Figure 7—figure supplement 1—source data 1. [file elife-84238-fig7-figsupp1-data1.zip › z Figure 7-Figure supplement 1- Source Data 1/Figure 7-Figure supplement 1- Source Data 1/original pics/C/ACTB.jpg]

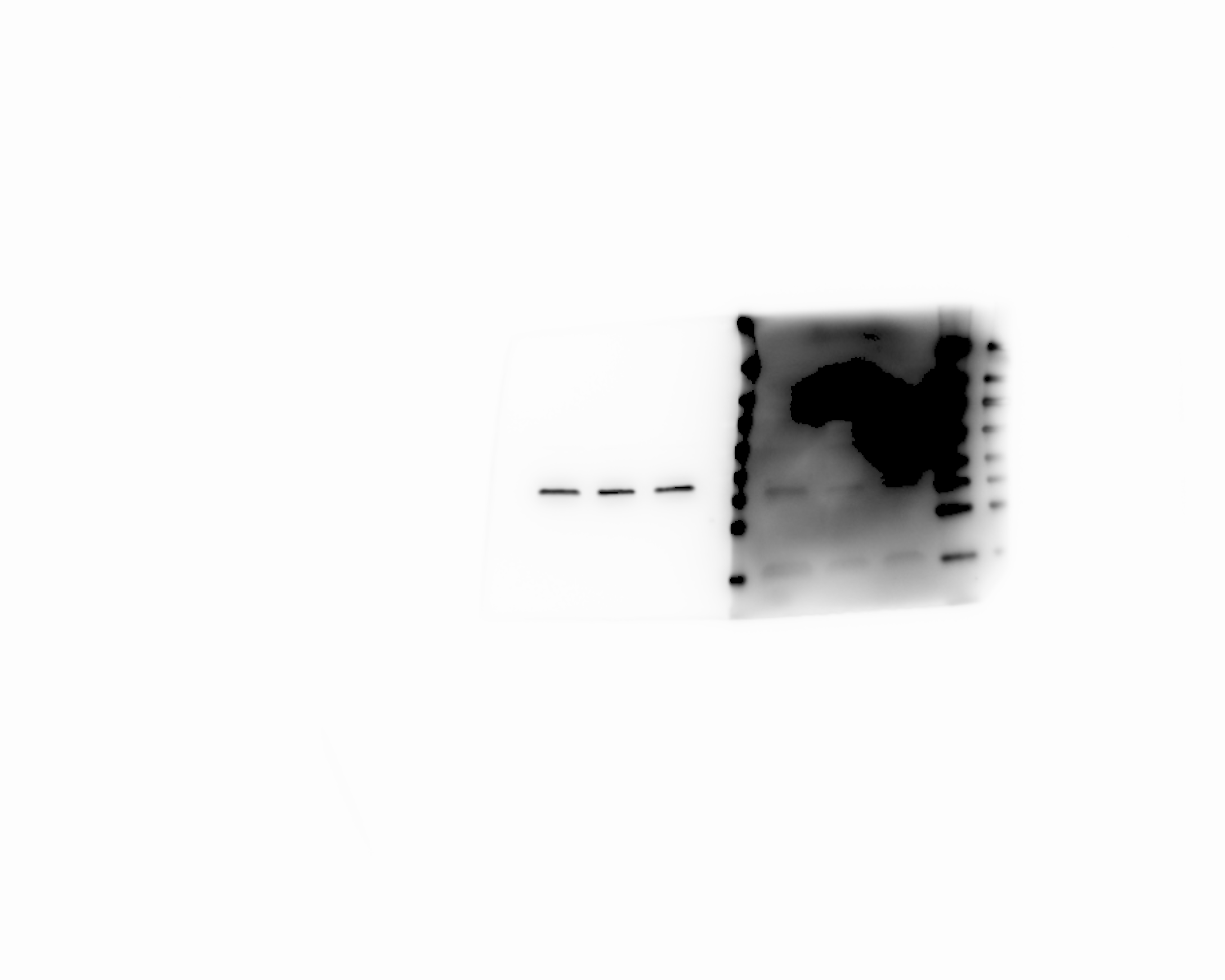

Supplement: Figure 7—figure supplement 1—source data 1. [file elife-84238-fig7-figsupp1-data1.zip › z Figure 7-Figure supplement 1- Source Data 1/Figure 7-Figure supplement 1- Source Data 1/original pics/D/ACTB.jpeg]

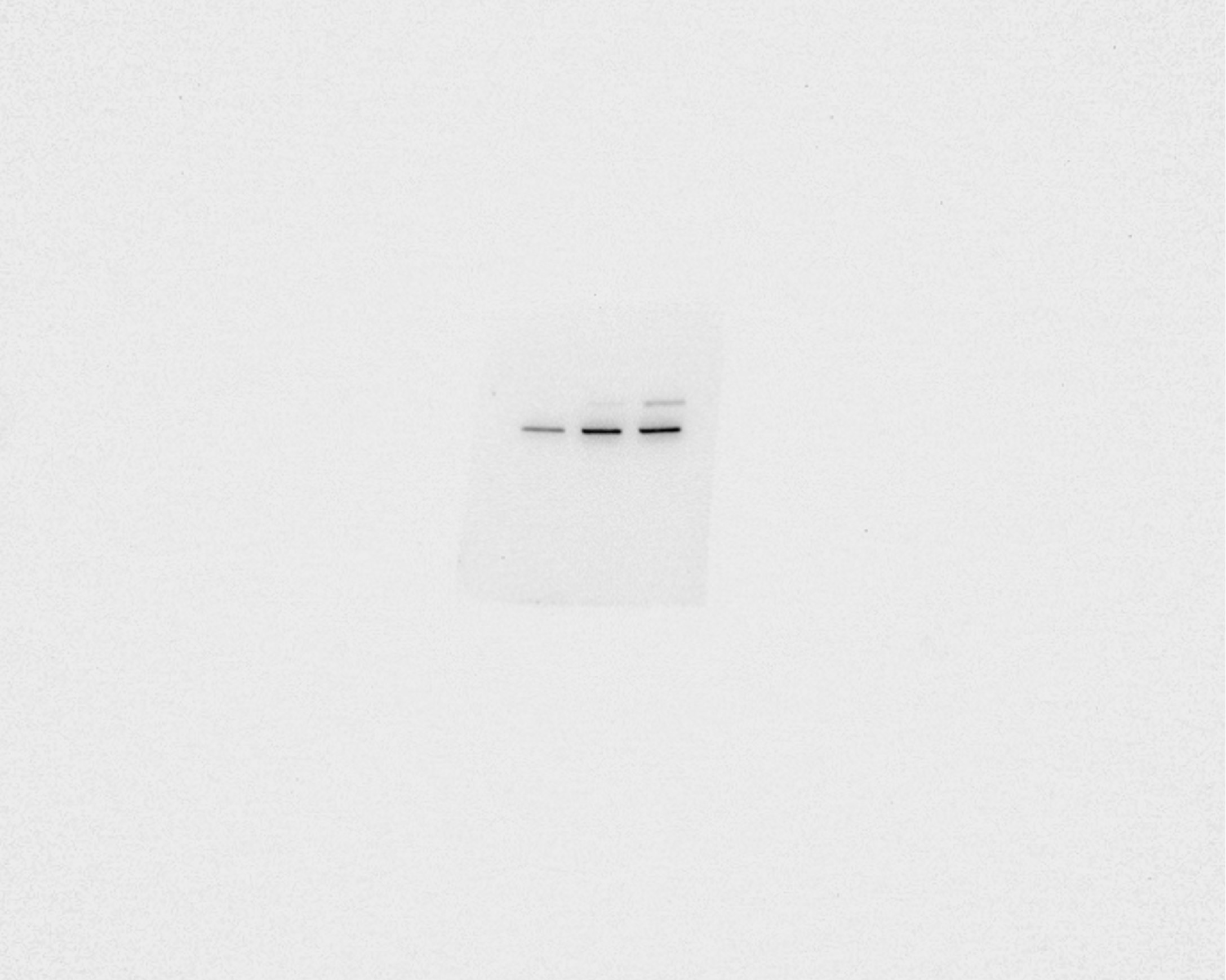

Supplement: Figure 7—figure supplement 1—source data 1. [file elife-84238-fig7-figsupp1-data1.zip › z Figure 7-Figure supplement 1- Source Data 1/Figure 7-Figure supplement 1- Source Data 1/original pics/D/p53 Pab 240.jpeg]
