# Supplementary material for: Pleiotropic effects of BAFF on the senescence-associated secretome and growth arrest
Source: eLife. 2023 Apr 21;12:e84238. doi: 10.7554/eLife.84238 (PMC10121226; doi:10.7554/eLife.84238)

Rossi et al, uncropped blots for Fig. 2G

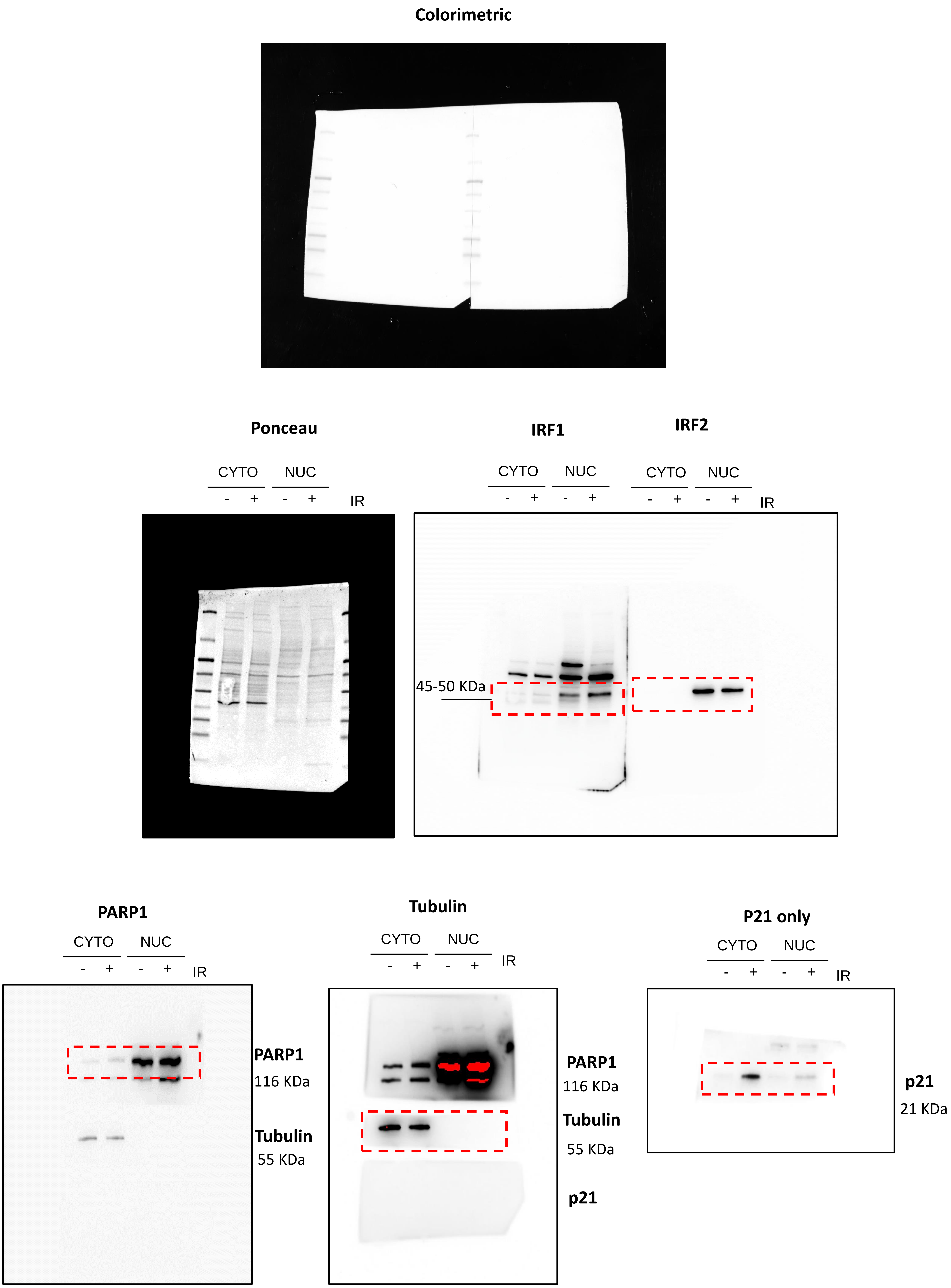

Supplement: Figure 2—source data 2. [file elife-84238-fig2-data2.zip › z Figure 2-Source Data 2/Figure 2-Source Data 2/Figure 2 uncropped blots labeled.pdf]
